# Performance of the Framingham risk models and pooled cohort equations for predicting 10-year risk of cardiovascular disease: a systematic review and meta-analysis

**DOI:** 10.1186/s12916-019-1340-7

**Published:** 2019-06-13

**Authors:** Johanna A. Damen, Romin Pajouheshnia, Pauline Heus, Karel G. M. Moons, Johannes B. Reitsma, Rob J. P. M. Scholten, Lotty Hooft, Thomas P. A. Debray

**Affiliations:** 1 Cochrane Netherlands, University Medical Center Utrecht, Utrecht University, Utrecht, The Netherlands; 2 Julius Center for Health Sciences and Primary Care, University Medical Center Utrecht, Utrecht University, P.O. Box 85500, Str. 6.131, 3508 GA Utrecht, The Netherlands

**Keywords:** Cardiovascular disease, Prediction models, Prognosis, Systematic review, Meta-analysis

## Abstract

**Background:**

The Framingham risk models and pooled cohort equations (PCE) are widely used and advocated in guidelines for predicting 10-year risk of developing coronary heart disease (CHD) and cardiovascular disease (CVD) in the general population. Over the past few decades, these models have been extensively validated within different populations, which provided mounting evidence that local tailoring is often necessary to obtain accurate predictions. The objective is to systematically review and summarize the predictive performance of three widely advocated cardiovascular risk prediction models (Framingham Wilson 1998, Framingham ATP III 2002 and PCE 2013) in men and women separately, to assess the generalizability of performance across different subgroups and geographical regions, and to determine sources of heterogeneity in the findings across studies.

**Methods:**

A search was performed in October 2017 to identify studies investigating the predictive performance of the aforementioned models. Studies were included if they externally validated one or more of the original models in the general population for the same outcome as the original model. We assessed risk of bias for each validation and extracted data on population characteristics and model performance. Performance estimates (observed versus expected (OE) ratio and c-statistic) were summarized using a random effects models and sources of heterogeneity were explored with meta-regression.

**Results:**

The search identified 1585 studies, of which 38 were included, describing a total of 112 external validations. Results indicate that, on average, all models overestimate the 10-year risk of CHD and CVD (pooled OE ratio ranged from 0.58 (95% CI 0.43–0.73; Wilson men) to 0.79 (95% CI 0.60–0.97; ATP III women)). Overestimation was most pronounced for high-risk individuals and European populations. Further, discriminative performance was better in women for all models. There was considerable heterogeneity in the c-statistic between studies, likely due to differences in population characteristics.

**Conclusions:**

The Framingham Wilson, ATP III and PCE discriminate comparably well but all overestimate the risk of developing CVD, especially in higher risk populations. Because the extent of miscalibration substantially varied across settings, we highly recommend that researchers further explore reasons for overprediction and that the models be updated for specific populations.

**Electronic supplementary material:**

The online version of this article (10.1186/s12916-019-1340-7) contains supplementary material, which is available to authorized users.

## Background

Cardiovascular disease (CVD) is a major health burden, accounting for 17.5 million deaths worldwide in 2012 [1]. To effectively and efficiently implement preventive measures such as lifestyle advice and lipid-lowering drugs, early identification of high-risk individuals for targeted intervention using so-called CVD risk prediction models or risk scores is widely advocated [2]. Evidently, it is crucial that predictions of CVD risk provided by these models are sufficiently accurate. Inappropriate risk-based management may lead to overtreatment or undertreatment. Clinical guidelines from the National Cholesterol Education Program and the American College of Cardiology and American Heart Association (AHA) advise using the Framingham Adult Treatment Panel (ATP) III model [3] or the pooled cohort equations (PCE) to predict 10-year risk of CVD for all individuals 40 years or older [2]. Also, most clinical research focused on studying the Framingham Wilson model [4, 5].

All three models have been externally validated numerous times across different settings and populations, with most studies showing that their predicted risks are too high (i.e. poor calibration, see Table 1) [6–9], while other reports found adequate calibration for these same models [10, 11]. Previous reviews have summarized existing models for cardiovascular risk prediction without undertaking any formal comparison or quantitative synthesis [5, 12–14] or focussed solely on the performance of the PCE [15]. Systematic reviews, followed by a quantitative synthesis, have become a vital tool in the evaluation of a prediction model’s performance across different settings and populations, and thus to better understand how the implementation of a developed model may affect clinical practice [16]. It may therefore help researchers, policy makers and clinicians to evaluate which models can be advocated in (new) guidelines for use in daily practice, and to what extent they should be updated prior to implementation.

In this review, we focus on the ATP III and PCE models as these are advocated in the clinical guidelines in the USA [2, 3]. Although Framingham Wilson is not mentioned in the clinical guidelines, it is relevant to review this prediction model, since many studies in the field of CVD risk prediction have externally validated this prediction model and have used it to assess the incremental value of new predictors or for comparison with newly developed prediction models [5]. We did not include other prediction models such as the SCORE [21] or QRISK [22] models in this review, as these were developed on European populations and we wanted to focus on truly competing models for the American population.

We, therefore, compared the predictive performance of the originally developed Framingham Wilson, Framingham ATP III and PCE models (see Additional file 1 for details on these prediction models and our review question). We conducted a systematic review, including critical appraisal, of all published studies that externally validated one or more of these three models, followed by a formal meta-analysis to summarize and compare the overall predictive performance of these models and the predictive performance across pre-defined subgroups. We explicitly did not intend to review all existing CVD risk prediction models but focused on these three most widely advocated and studied models in the USA.

## Methods

We conducted our review based on the steps described in the CHecklist for critical Appraisal and data extraction for systematic Reviews of prediction Modelling Studies (CHARMS) [23] and in a guidance paper on the systematic review and meta-analysis of prediction models [16]. A review protocol is available in Additional file 2. This review is reported according to the MOOSE reporting checklist (Additional file 3).

### Search and selection

We started with studies published before June 2013 that were already identified in two previously published systematic reviews [5, 12]. Studies published after June 2013 were identified according to the following strategy, developed by an information specialist working for Cochrane. First, a search was performed in MEDLINE and Embase (October 25, 2017, Additional file 4). In addition, a citation search in Scopus and Web of Science was performed to find all studies published between 2013 and 2017 that cited the studies in which the development of one of the original models was described (Additional file 4). All studies that were identified both by the search in MEDLINE and Embase, and the citation search were screened for eligibility, first on title and abstract by one reviewer and subsequently on full text by two independent reviewers. Disagreements were solved in group discussions. The reference lists of systematic reviews identified by our search were screened to identify additional studies.

### Eligibility criteria

Studies were eligible for inclusion if they described the external validation of Framingham Wilson 1998 [4], Framingham ATP III 2002 [3] and/or PCE 2013 [10]. Studies were included if they externally validated these models for fatal or nonfatal coronary heart disease (CHD) in the case of Framingham Wilson and ATP III, and hard atherosclerotic CVD (here referred to as fatal or nonfatal CVD) in the case of PCE, separately for men and women, in a general (unselected) population setting. For Framingham Wilson and ATP III, we thus excluded external validation studies that used the combination of CHD and stroke as an outcome, while we included studies with so called ‘hard CHD’ (myocardial infarction + fatal CHD). For PCE, we excluded studies that used CHD as outcome. Studies regarding specific patient populations (e.g. patients with diabetes) were excluded. Studies in which the model was updated or altered (e.g. recalibration or model revision [24, 25], see Table 1) before external validation were excluded if they did not provide any information on the original model’s performance. Studies in which the models for men and women were combined in one validation (with one performance measure reported for men and women together instead of two separate performance measures) were excluded. Studies that assessed the incremental value of an additional predictor on top of the original model were also excluded, unless the authors explicitly reported on the external validity of the original model before adding the extra predictor. When a study population was used multiple times to validate the same model (i.e. multiple publications describing a certain study cohort), the external validation with eligibility criteria and predicted outcome that most closely resembled our review question was included, to avoid introducing bias because of duplicate data [26].

**Table 1:** Terminology

	Definition
Case-mix/patient spectrum	Characteristics of the study population (e.g. age, gender distribution)
Prediction horizon	Time frame in which the model predicts the outcome (e.g. predicting 10-year risk of developing a CVD event).
External validation	Estimating the predictive performance of an existing prediction model in a dataset or study population other than the dataset from which the model was developed.
Predictive performance	Accuracy of the predictions made by a prediction model, often expressed in terms of discrimination or calibration.
Discrimination	Ability of the model to distinguish between people who did and did not develop the event of interest, often quantified by the c-statistic.
Concordance (c)-statistic	Statistic that quantifies the chance that for any two individuals of which one developed the outcome and the other did not, the former has a higher predicted probability according to the model than the latter. A c-statistic of 1 means perfect discriminative ability, whereas a model with a c-statistic of 0.5 is not better than flipping a coin [17].
Calibration	Agreement between observed event risks and event risks predicted by the model.
Observed versus expected (OE) ratio	The ratio of the total number of outcome events that occurred (e.g. in 10 years) and the total number of events predicted by the model. The OE ratio can be calculated for the entire study population (further referred to as ‘total OE ratio’), or in categories of predicted risks.
Calibration slope	Measure that gives an indication of the strength of the predictor effects. The calibration slope ideally equals 1. A calibration slope < 1 indicates that predictions are too extreme (low-risk individuals have a predicted risk that is too low, and high-risk individuals are given a predicted risk that is too high). Conversely, a slope > 1 indicates that predictions are too moderate [18, 19].
Model updating/recalibration	When externally validating a prediction model, adjusting the model to the dataset in which the model is validated, to improve the predictive performance of the model.
Updating the baseline hazard or risk	When externally validating a prediction model, adapting the original baseline hazard or intercept of the prediction model to the dataset in which the model is validated. This updating method corrects for differences in observed outcome incidence between the original development and external validation dataset.
Updating the common slope	When externally validating a prediction model, adapting the beta coefficients of the model using a single correction factor, to proportionally adjust for changes in predictor outcome associations [20].
Model revision	Taking the predictors of an existing previously developed model and fitting these in the external dataset by estimating the new predictor-outcome associations (e.g. regression coefficients).

### Data extraction and critical appraisal

For each included study, data were extracted on study design, population characteristics, participant enrolment, study dates, prediction horizon, predicted outcomes, predictors, sample size, model updating methods and model performance (Additional file 5). Risk of bias was assessed based on a combination of the CHARMS checklist [23] and a preliminary version of the Cochrane Prediction study Risk Of Bias Assessment Tool (PROBAST) [27, 28] (Additional file 5). Risk of bias was assessed for each validation, across five domains: participant selection (e.g. study design, in- and exclusions), predictors (e.g. differences in predictor definitions), outcome (e.g. same definition and assessment for every participant), sample size and participant flow (e.g. handling of missing data), analyses (e.g. handling of censoring). After several rounds of piloting and adjusting the data extraction form in a team of three reviewers, data were extracted by one of the three reviewers. Risk of bias was independently assessed by pairs of reviewers. Disagreements were solved after discussion or by a third reviewer.

Information was extracted on model discrimination and calibration, before and, if reported, after model updating, in terms of the reported concordance (c)-statistic and total observed versus expected (OE) ratio. If relevant information was missing (e.g. standard error of performance measure or population characteristics), we contacted the authors of the corresponding study. If no additional information could be obtained, we approximated missing information using formulas described by Debray et al. [16] (Additional file 6). If reported, calibration was also extracted for different risk categories. If the OE ratio was reported for shorter time intervals (e.g. 5 years), we extrapolated this to 10 years by assuming a Poisson distribution (Additional file 6).

### Statistical analyses

We performed meta-analyses of the OE ratio and the c-statistic for 10-year risk predictions. Based on previous recommendations [16, 29], we pooled the log OE ratio and logit c-statistic using random-effects meta-analysis. Further, we stratified the meta-analysis by model and gender, resulting in six main groups: Wilson men, Wilson women, ATP III men, ATP III women, PCE men, PCE women. We calculated 95% confidence intervals (CI) and (approximate) 95% prediction intervals (PI) to quantify uncertainty and the presence of between-study heterogeneity. The CI indicates the precision of the summary performance estimate, and the PI provides boundaries on the likely performance in future model validation studies that are comparable to the studies included in the meta-analysis, and can thus be seen as an indication of model generalizability (Additional file 7) [30]. The observed and predicted probabilities were plotted in risk categories against each other and combined into a summary estimate of the calibration slope using mixed effects models (Additional file 7).

Since between-study heterogeneity in estimates of predictive performance is expected due to differences in the design and execution of validation studies [16], we investigated whether the c-statistic differed between validation studies with different eligibility criteria or actual case-mix. Furthermore, we performed univariable random effects meta-regression analyses to investigate the influence of case-mix differences (e.g. due to differences in eligibility criteria) on the OE ratio and c-statistic (Additional file 7). Several pre-specified sensitivity analyses were performed in which we studied the influence of risk of bias and alternative weighting methods in the meta-analysis on our findings (Additional file 7). All analyses were performed in R version 3.3.2 [31] using the packages metafor [32], mvmeta [33], metamisc [34] and lme4 [35].

## Results

### Identification and selection of studies

**Fig. 1:**
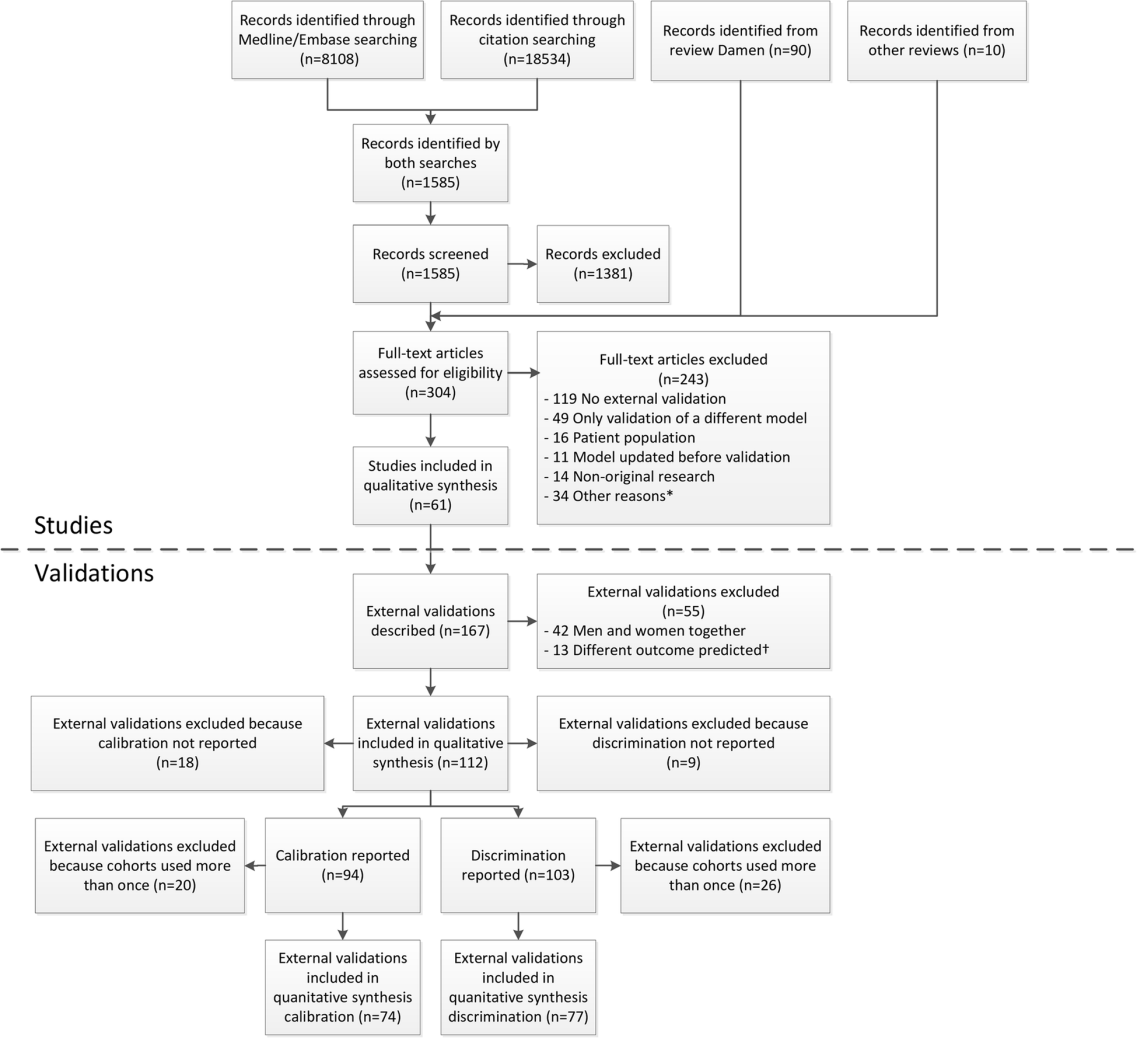
Flow diagram of selected studies. Two searches were performed; one in MEDLINE and Embase and one in Scopus and Web of Science. Only studies identified by both searches were screened for eligibility, supplemented with records identified from previous systematic reviews. One study could describe more than one external validation (e.g. one for men and one for women); therefore, 61 studies described 167 external validations. Calibration was reported in 94 validations (41 directly reported, 19 provided by the authors on request, 34 estimated from calibration tables and calibration plots), and discrimination in 103 validations (91 c-statistics directly reported, 12 provided by the authors on request. Precision of c-statistic: 45 directly reported, 24 provided by the authors, 32 estimated from the sample size and 2 not reported). Some external validations were excluded because cohorts were used more than once to validate the same model (Additional file 9). *For example, no cardiovascular outcome and not written in English. ^†^The Framingham Wilson and ATP III models were developed to predict the risk of fatal or nonfatal coronary heart disease, and the PCE model was developed to predict the risk of fatal or nonfatal cardiovascular disease. External validations that used a different outcome were excluded from the analyses (Additional file 8)

We first identified 100 potentially eligible studies from previously conducted systematic reviews. An additional search identified 1585 studies since June 2013 (Fig. 1). Of these 1685 studies, 304 studies were screened on full-text and data were extracted for 61 studies, describing 167 validations of the performance of one or more of the three models. Finally, 38 studies (112 validations) met our eligibility criteria [6–11, 36–67].

### Description of included validations

In 112 validations (Additional file 10), the Framingham Wilson model was validated 38 times (men 23, women 15), Framingham ATP III 13 times (men 7, women 6) and PCE 61 times (men 30, women 31). One study performed a direct (head-to-head) comparison of all six prediction models [7] and one other study performed a direct comparison of the ATP III and PCE models [6]. Study participants were recruited between 1965 and 2008 and originated from North America (56), Europe (29), Asia (25) and Australia (2). All outcome definitions are described in Additional file 8. The median event rate across the included validations was 4.4% (IQR 2.8%–7.9%) and ranged between 0.5 and 29.4%. We excluded 18 and 9 external validations because the OE ratio and c-statistic, respectively, were not available, and subsequently excluded 20 and 26 external validations for the OE ratio and c-statistic, respectively, because cohorts were used multiple times to validate the same model (Additional file 9). This resulted in the inclusion of 74 validations in the analyses of the OE ratio and 77 validations in the analyses of the c-statistic (Fig. 1, Additional file 14).

### Risk of bias

**Fig. 2:**
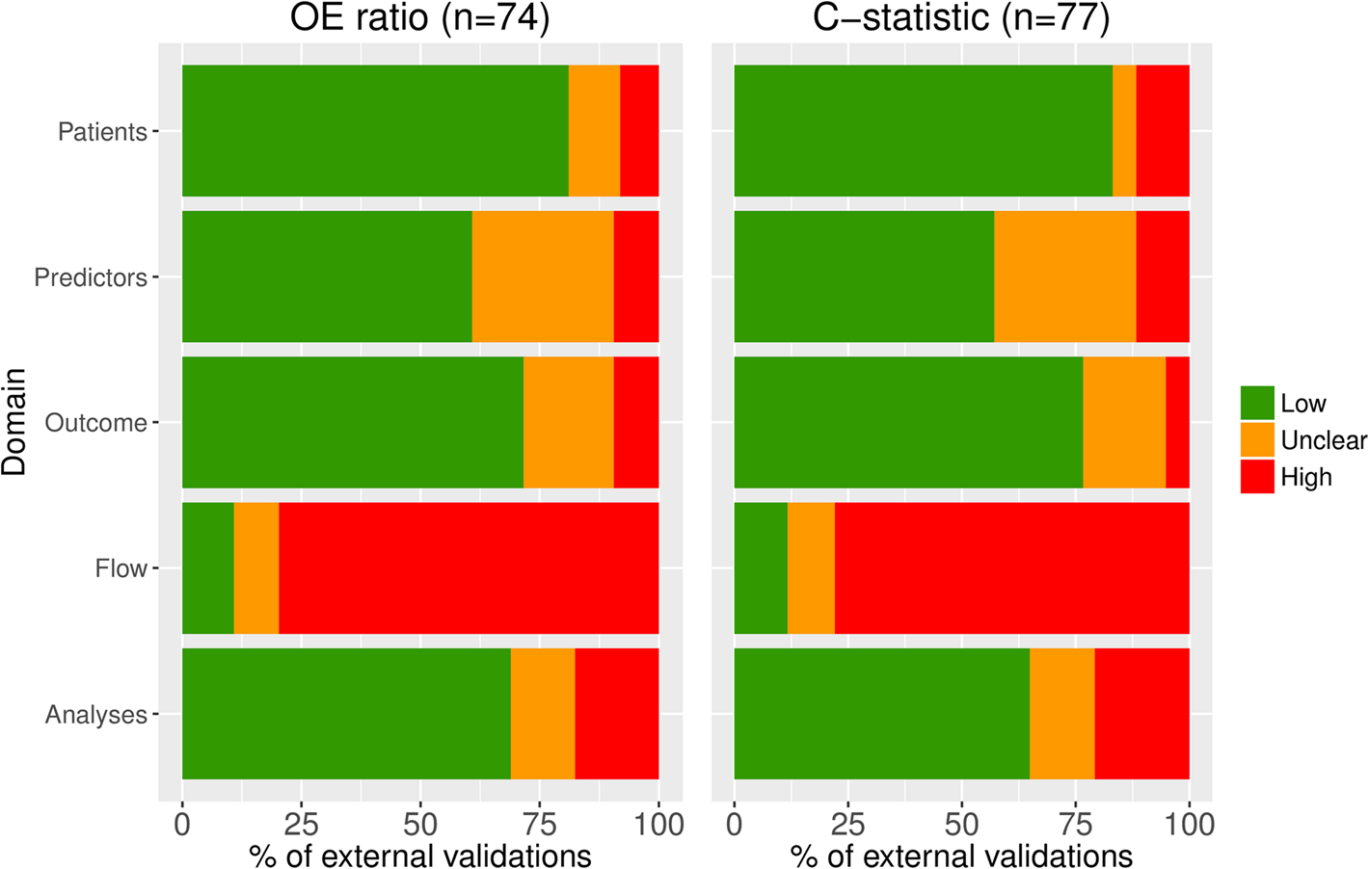
Risk of bias assessment. Summary of risk of bias assessments for validations included in the meta-analyses of OE ratio (74 validations) and c-statistic (77 validations)

For participant selection, most validations scored low risk of bias (*n* = 60 (81%) and *n* = 64 (83%) for validations reporting OE ratio and c-statistic, respectively, Fig. 2). Risk of bias for predictors was often unclear (*n* = 22 (30%) and *n* = 24 (31%) for OE ratio and c-statistic) due to poor reporting of predictor definitions and measurement methods. Most validations scored low risk of bias on outcome (*n* = 53 (72%), *n* = 59 (77%)). More than three quarters of the validations scored high risk of bias for sample size and participant flow (*n* = 59 (80%) and *n* = 60 (78%)), often due to inadequate handling of missing data (i.e. simply ignoring). Low risk of bias was scored for the analysis in 51 (70%) and 50 (65%) validations, for OE ratio and c-statistic respectively. In total, 62 (84%) and 63 (82%) validations scored high risk of bias for at least one domain, and 4 (5%) and 6 (8%) validations scored low risk of bias for all five domains, for OE ratio and c-statistic, respectively.

### Calibration

**Fig. 3:**
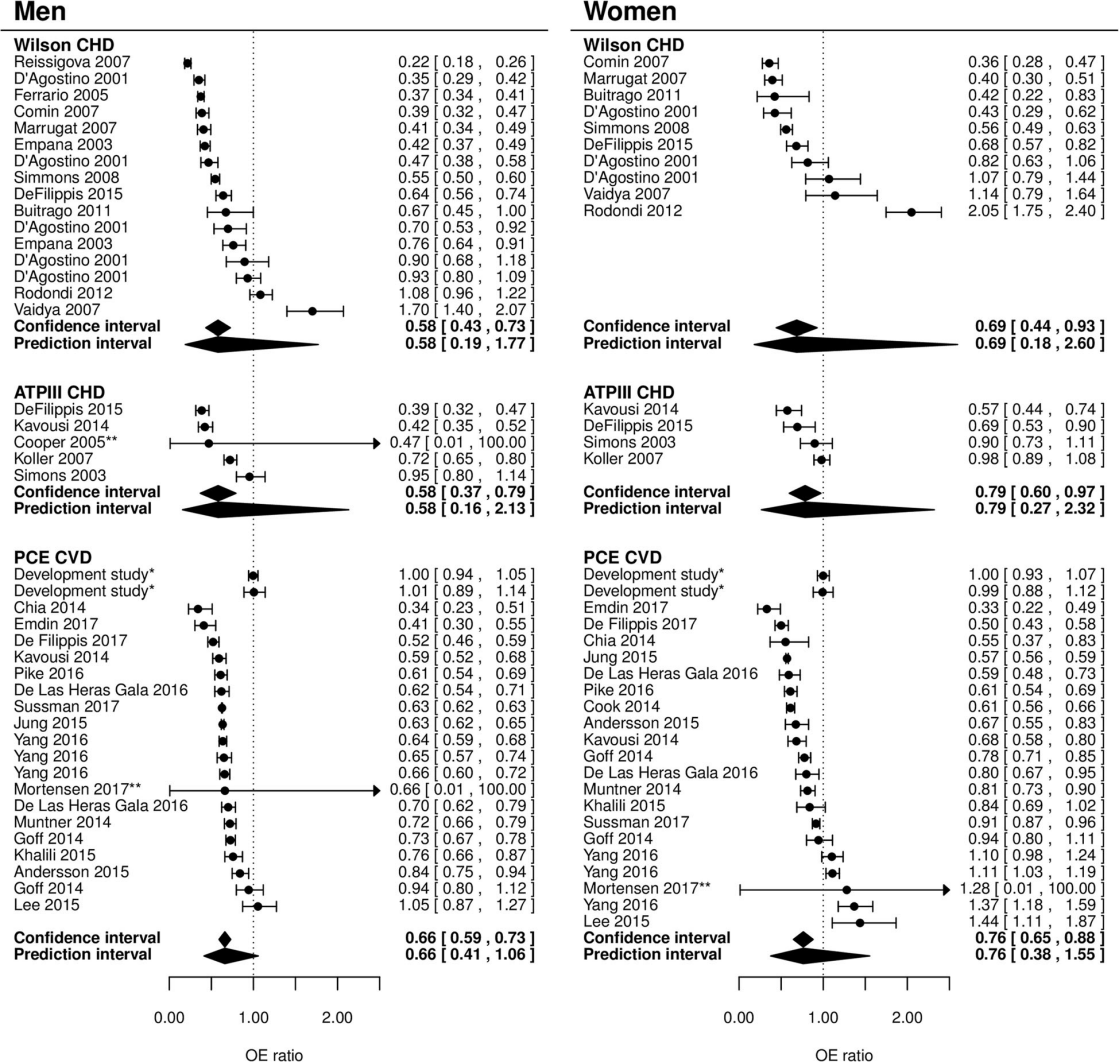
Forest plots of the OE ratio in external validations. Ninety-five percent confidence intervals and 95% prediction intervals per model are indicated. The performance of the model in the development study is shown in the first rows (only reported for PCE). This estimate is not included in calculating the pooled estimate of performance. *Performance of the model in the development population after internal validation. The first row contains the performance of the model for Whites, the second for African Americans. **Standard error was not available. CHD: Coronary heart disease, CVD: cardiovascular disease

Figure 3 shows the calibration of the six main models, as depicted by their 10-year total OE ratio. For 24 out of 74 validations (32%), maximum follow-up was shorter than 10 years. For 20 out of these 24 (83%), information was available to extrapolate the OE ratio to 10 years. Most studies showed overprediction, indicating that 10-year risk predictions provided by the models were typically higher than observed in the validation datasets. For the Wilson model, the number of events predicted by the model was lower than the actual number of events in two studies (one in healthy siblings of patients with premature coronary artery disease [66], and one in community-dwelling individuals aged 70–79 [60]). For the PCE, underestimation of the number of events occurred in Chinese [51] and Korean [47] populations.

**Fig. 4:**
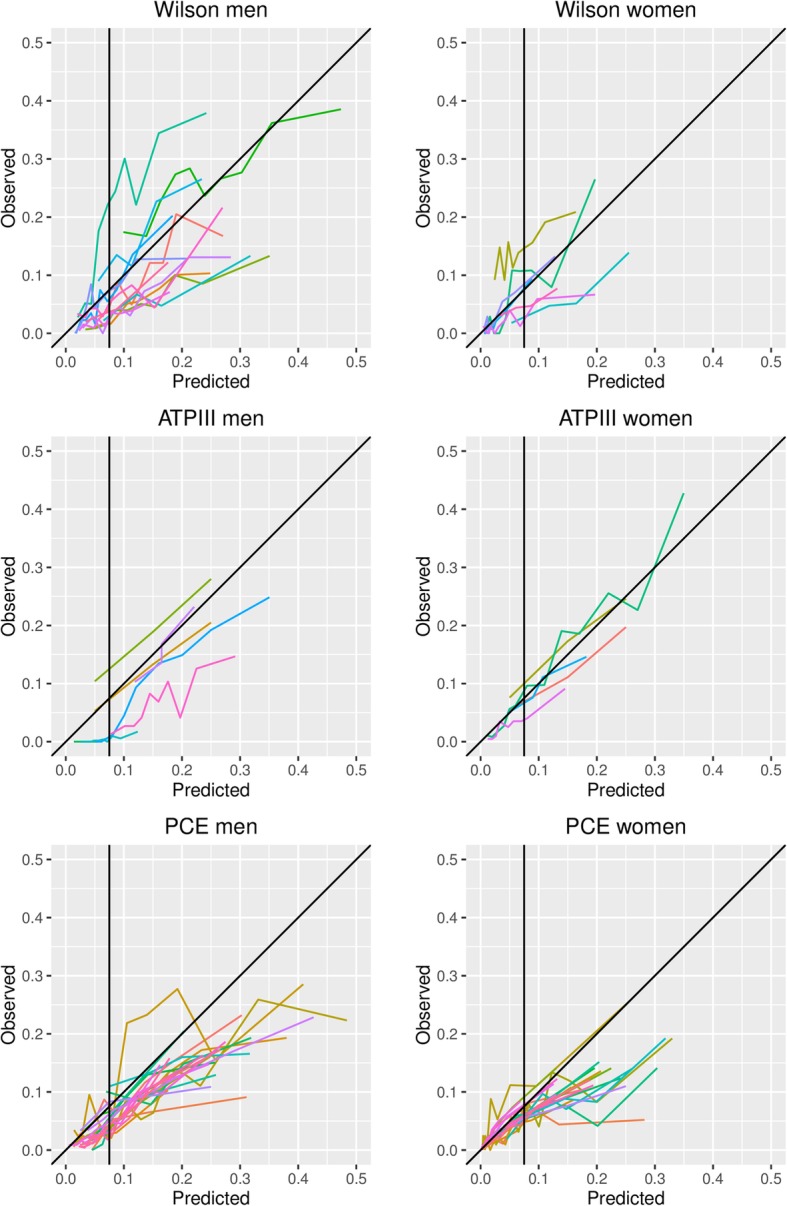
Calibration plots of the Framingham Wilson, ATP III and PCE models. Each line represents one external validation. The diagonal line represents perfect agreement between observed and predicted risks. All points below that line indicate that more events were predicted than observed (overprediction) and points above the line indicate fewer events were predicted than observed (underprediction). The vertical black line represents a treatment threshold of 7.5% [68].

Meta-analysis revealed a considerable degree of between-study heterogeneity in OE ratios (Fig. 3), but with clear overprediction, as summary OE ratios ranged from 0.58 (Wilson men and ATP III men) to 0.79 (ATP III women). Additional analyses revealed that overprediction is more pronounced in high-risk patients for all models (Fig. 4). The results of the summary calibration slope suggest that miscalibration of the Framingham Wilson and ATP III models, and PCE men model was mostly related to heterogeneity in baseline risk (as the summary calibration slope is close to 1), while for PCE women we found a slope around 0.8, suggesting that this model was overfitted or does not transport well to new populations (Additional file 11).

### Discrimination

**Fig. 5:**
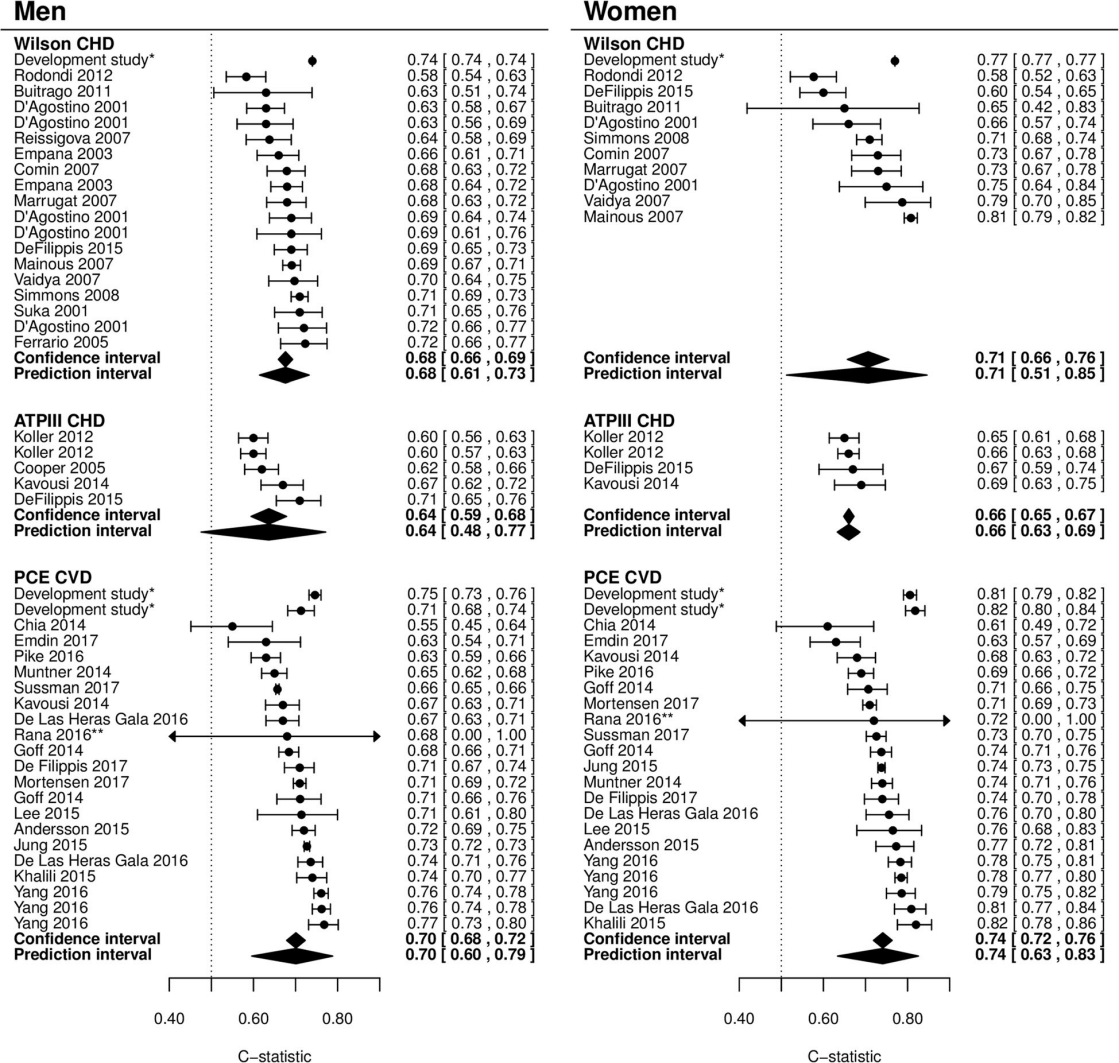
Forest plots of c-statistic in external validations. Ninety-five percent confidence intervals and 95% prediction intervals per model are indicated. The performance of the model in the development study is shown in the first row(s) (not reported for the ATP III model) and is not included in the pooled estimate of performance. *Performance of the model in the development population (Wilson (no standard error reported)) and after 10 × 10 cross-validation (PCE). For the PCE, the first row contains the performance of the White model and the second the African American model. **Standard error was not available. CHD: coronary heart disease, CVD: cardiovascular disease

For all models, discriminative performance was slightly better for women than for men, although there was considerable variation between studies (Fig. 5). One head-to-head comparison of all three models showed worse discriminative performance for the Wilson model compared to the ATP III and PCE models [7].

### Sensitivity analyses

Sensitivity analyses revealed no effect of study quality and different weighting strategies on the pooled performance of the models, both for calibration and discrimination (Additional file 12).

### Factors that influence performance of the models

**Fig. 6:**
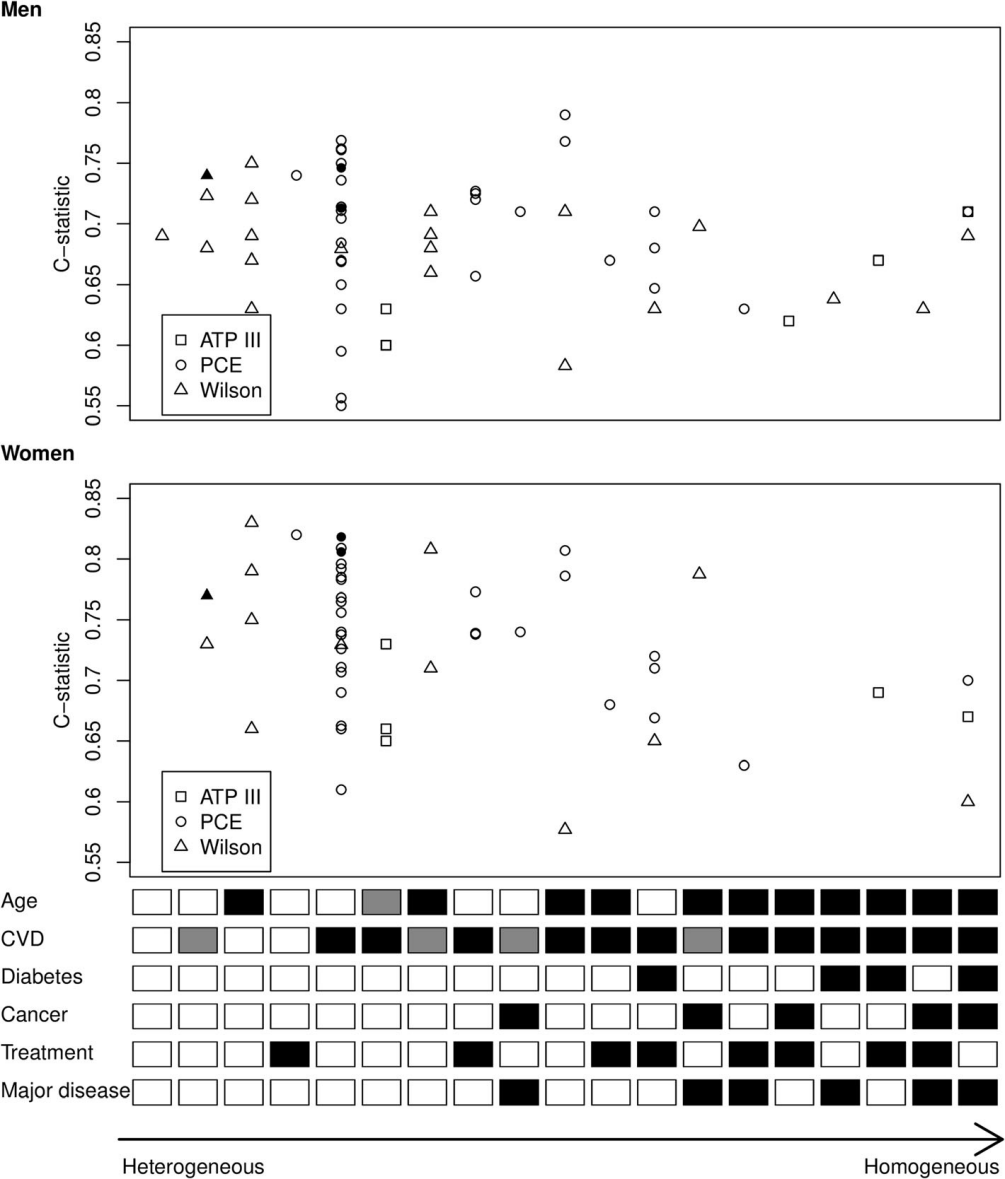
C-statistic for different combinations of eligibility criteria. The open squares, circles and triangles represent validations of the ATP III, PCE and Wilson model, respectively. The black circles and triangles represent the performance of the PCE models for Whites and African-Americans, and Wilson models, in the development populations. Lower part: for age, white means a broad age range was included (difference between upper and lower age limit > 30 years), black means a narrow age range was included (difference between upper and lower age limit ≤ 30 years) and grey means age was not reported. For CVD, white means no exclusion of people with CHD or CVD, grey means people with previous CHD events were excluded from the study and black means people with previous CVD events were excluded from the study. For diabetes, cancer and major disease, white means that no restrictions were reported and black means that people with these conditions were excluded. For treatment, white means no restrictions and black means people who were receiving any treatment to lower their risk of CVD (e.g. anti-hypertensives) were excluded from the study

For women, the highest c-statistics were reported in studies with a large variety in case-mix. For men, such a trend was not visible (Fig. 6). The OE ratio for the Wilson model in the USA was closer to 1 compared to Europe, but the number of external validations per subgroup was very small (Additional file 13). Furthermore, the OE ratio appeared to decrease (further away from 1, i.e. more overprediction) with increasing mean total cholesterol. No evidence was found of an association between the OE ratio and other case-mix variables or the start date of participant recruitment. The c-statistic appeared to decrease with increasing mean age, mean systolic blood pressure and standard deviation of HDL cholesterol, and to increase with increasing standard deviation of age and total cholesterol (Additional file 13). No statistically significant associations were found between the c-statistic and other variables.

### Performance after updating

**Fig. 7:**
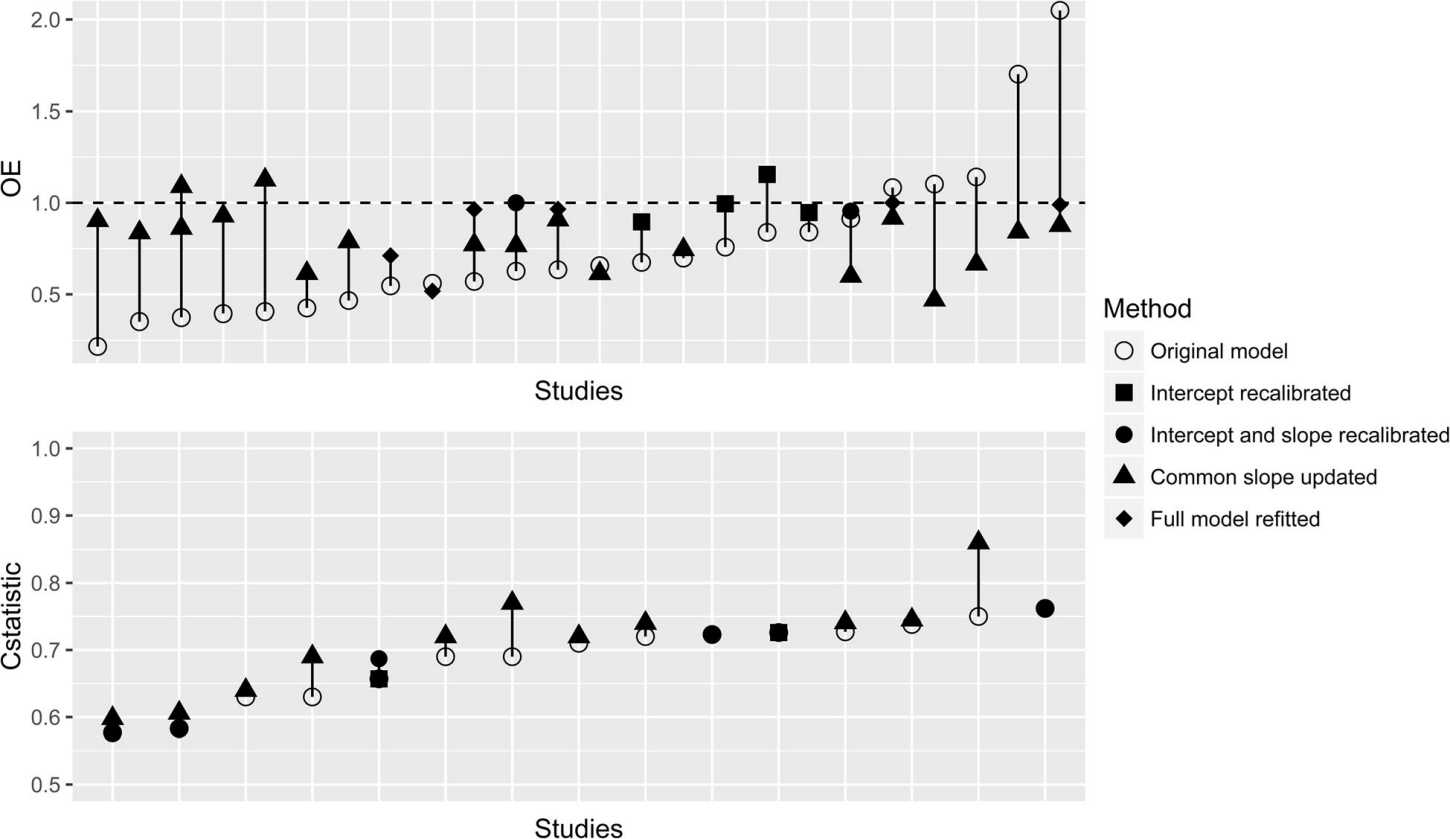
Performance of models before and after update. The *x*-axis is sorted by performance before updating. The lines connect performance of models in the same cohort before and after updating

For 40 validations, the model was subsequently updated, of which 24 reported the OE ratio and 15 reported the c-statistic after updating (Fig. 7). We found that substantial improvements in OE ratio were often obtained by simply re-estimating the baseline hazard or the common slope. More advanced methods of updating (e.g. entire revision of the model) did not offer much additional improvements. For the c-statistic, only advanced methods of updating resulted in limited improvement.

## Discussion

### Summary of findings

We systematically reviewed the performance of the Framingham Wilson, Framingham ATP III and PCE models for predicting 10-year risk of CHD or CVD for men and women separately in the general population. We found only small differences in pooled performance between the three models, but large differences in performance between validations of the same model across different populations. Although we mostly had to rely on indirect comparisons of the models, we found that performance of all three models was consistently better in women than in men for both discrimination and calibration. We found that all models overestimated the risk of CHD or CVD if they were not updated locally prior to implementation. This overestimation was more pronounced in European populations compared to the USA. Overprediction clearly declined when the validated models were adjusted (e.g. via updating the baseline hazard) to the validation setting at hand. This indicates that although the Framingham models and PCE have the potential to be effective tools for patient management in clinical care, their use should be preceded by local adjustments of the baseline hazard. If aforementioned models are implemented as originally developed to guide treatment decisions, over- or undertreatment of individuals may occur and therefore introduce unnecessary costs or even harm. Unfortunately, current guidelines do not recommend local tailoring of Framingham and PCE and may therefore lead to suboptimal decision making. Although it was not possible to identify key sources of heterogeneity, we found that discriminative performance tends to increase as populations become more diverse, i.e. with a wider case-mix. This effect has previously been explained [69–71].

### Comparison with previous literature

Our findings are in agreement with previous studies, which also found that the Framingham prediction models overestimate the risk of CHD in the general population [12, 13] and that (overall) calibration is better in the USA than in European populations [14]. Furthermore, the overestimation of risk was more pronounced in more recent populations than in earlier study populations [12, 13]. A recent study found that the PCE overestimate the risk of CVD [15], and in another study, the better discrimination seen in women was attributed to a stronger association between risk factors and CVD in women compared to men [72]. In addition to these previous reviews, we compared the predictive performance of the three prediction models that are currently in the medical guideline and that are most often evaluated in external validation studies, and found that these models have similar performance. Further, we did an extensive evaluation of factors possibly associated with heterogeneity in predictive performance and we found that predictive performance improved if these existing models were being updated.

### Reasons for overprediction

There could be several reasons for the observed overprediction. These reasons have also been extensively discussed previously with regard to the PCE [15, 73]. First, differences in eligibility criteria (e.g. the exclusion of participants with previous CVD events) across validation studies may have affected calibration. Second, the three prediction models have been (partly) developed using data from the 1970s, and since then, treatment of people at high risk for a CVD event has changed considerably, such as the introduction of statins in 1987 [74]. The increased use of effective treatments over time aimed at preventing CVD events will have lowered the observed number of events in more recent validation studies, resulting in overestimation of risk in these validation populations [39, 75, 76]. This would also explain why overprediction was most pronounced in high-risk individuals and why we found more overprediction in studies with increasing mean total cholesterol levels. We hypothesized that the degree of overprediction would increase over the years [12, 13, 77]; however, this could not be confirmed statistically. About one third of validations of the PCE excluded participants receiving treatment to lower CVD risk at baseline, but we found no difference in performance between validations that did or did not exclude these participants. However, as the use of risk-lowering medication during follow-up was rarely reported in these studies, we cannot rule out an effect of incident treatment use on model performance [76]. Following the recently issued Transparent Reporting of a multivariable prediction model for Individual Prognosis or Diagnosis (TRIPOD) guideline [78, 79] and the guidance on adjusting for treatment use in prediction modelling studies [75, 76, 80], we also strongly recommend investigators of future prediction model studies to record the use of treatment during follow-up. Third, in agreement with previously published reviews [12–15], we found more overestimation of risk in European populations compared to those of the USA whereas in some Asian populations an underestimation was seen. Both suggest that differences between these populations in, for example, unmeasured CVD risk factors and in the use of preventive CVD strategies (e.g. medical treatment or lifestyle programs) are responsible. Unfortunately, not enough information was available to study the role of ethnicity on the heterogeneity of the models’ predictive accuracies. Finally, rather than overprediction by the models, there could also be issues in the design of the external validation studies that give rise to a lower number of identified events. Underascertainment or misclassification of outcome events, unusually high rates of people receiving treatment, short follow-up duration, and inclusion of ethnicities not included in development of the models, have been mentioned as reasons for the overprediction we also observed [57, 81–84]. Also outcome definitions can be different between the development and validation studies. This seems particularly a problem for the Framingham Wilson model, as half of the validation studies did not include angina in their outcome definition. Researchers have however shown that overestimation can often not fully be explained by treatment use and misclassified outcome events [39, 85].

### Implications for practice and research

According to the ACC-AHA guidelines [2], risk-lowering treatment is considered in people 40–75 years old, without diabetes, with LDL cholesterol levels between 70 and 189 mg/dl and with 10-year predicted risk of CVD ≥ 7.5%. After a discussion between clinician and patient about adverse effects and patient preferences, it is decided whether risk-lowering treatment is initiated. The overprediction observed in this review is problematic as this might change the population eligible for risk-lowering treatment. Unfortunately, this is true for all three CVD risk prediction models. As the meta-analysis indicates that overprediction does not consistently occur across different settings and populations, there is no simple solution to address this problem. From the studies that provided data on calibration in subgroups, we found that overestimation was more pronounced in high-risk individuals. When the (over)estimation of the absolute risk is already beyond the treatment probability threshold, it will not influence treatment decisions. However, overestimation of CVD risk might still influence the intensity (dose and frequency) of administered treatments and affect the patient’s behaviour. Although excessive risk estimates could stimulate patients to adopt a more healthy lifestyle (similarly to patients with more risk factors [86]), it could also cause unnecessary anxiety for future cardiovascular events. For people at lower risk, this might, however, result in crossing the treatment probability boundary when, actually, they are at lower risk. If we assume that the observed risks reported in the validation studies are not influenced by factors that changed during follow-up such as treatment use, we can state that in 82% of individuals the average predicted and observed percentages were both below or both above the treatment boundary of 7.5% (i.e. concordant points); in 1% of individuals, the predicted risk was below 7.5% and the observed risk was above 7.5% which would, on average, lead to undertreatment in these groups of individuals; and in 17% of individuals, the average predicted risk was above 7.5% while the average observed risk was below 7.5% which would, on average, lead to overtreatment. Though this does not mean that 17% of individual will receive unnecessary treatment, it is important that doctors realize that some patients may receive unnecessary treatment, resulting in adverse effects, patient’s burden and extra costs of treatment and monitoring.

The clinical implications of a certain c-statistic are even more difficult to predict. A low c-statistic (i.e. close to 0.5) indicates that the model discriminates no better than a coin toss, while a high c-statistic (close to 1.0) indicates that the model can perfectly separate people who develop an event from people who do not develop an event. If we have four pairs of individuals visiting the doctor, of which one individual will develop CVD in future (case) and one not (noncase), a c-statistic of 0.75 means that for 3 of these pairs, the case would indeed receive a higher predicted risk (which could still be too low or too high), while in one pair, the case would receive a lower predicted risk compared to the noncase.

In general, the performance of prediction models tends to vary substantially across different settings and populations, due to differences in case-mix and health care systems [87]. Hence, one external validation may not be sufficient to claim adequate performance and multiple validations are necessary to get insight in the generalizability of prediction models [71]. Primary external validation studies already showed the need for recalibration to improve a model’s calibration and better tailor it to the setting at hand. In this systematic review, we now show that this also holds when all studies are pooled together in a meta-analysis. We found that none of the models offer reliable predictions unless (at least) their baseline risk or hazard (and, if applicable, population means of the predictors in the model) are recalibrated to the local setting. Studies that reported performance of the model before and after update showed that performance indeed improves after update; however, the necessary adjustments vary from setting to setting and thus need to be evaluated locally [8, 11, 36, 47, 48, 54]. As previously emphasized, more extensive revision methods are often not needed [24, 25, 88]. Hence, it appears that conventional predictors, such as age, smoking, diabetes, blood pressure and cholesterol, are still relevant indicators of 10-year CHD or CVD risk, and their associations with CVD events have largely remained stable. The need for updating CVD risk prediction models has already been discussed more than 15 years ago [11, 89], but still nothing has changed. We believe this should change now, especially since nowadays applying simple model updating is becoming increasingly possible, due to improvements in the storage of the information required to update a model. Clinical guidelines should advocate that the performance of the models is not appropriate for every patient population, and recalibration before using the models is necessary. Nice examples of the tailoring of CVD risk prediction models to specific populations are the Globorisk prediction model, which can easily be tailored to different countries using country-specific data on the population prevalence of outcomes and predictors [90], and the SCORE model, which has been tailored to many European countries using national mortality statistics [21, 91–93].

Furthermore, it is very important to gain insight into factors that cause the observed overestimation of events. This can be studied in empirical data, like the study by Cook et al. [38] where hypothetical situations are created in which a number of events are missed or prevented by risk-lowering drugs. Also simulation studies might give more insight into reasons for the overestimation of CVD risk.

These suggestions, however, offer no short-term solution for practitioners currently using the three reviewed prediction models. For now, we advise practitioners in the USA to continue using the PCE, though being aware of the potential miscalibration especially in high-risk individuals. The PCE are currently advised to be used by the ACC-AHA clinical guidelines, which is understandable because alternatives have not been studied in enough detail. However, the actual value of the PCE in clinical practice is limited by their calibration. We therefore advise to recalibrate the model in the near feature to specific populations. Fortunately, systematic reviews have shown that the prevalence of common CVD risk factors decreases (e.g. cholesterol levels drop) in populations where CVD risk prediction models and their corresponding treatment guidance are being used [94, 95]. Furthermore, statins have been proven effective with limited adverse events [96]. Finally, we advise practitioners to choose a model that predicts a clinically relevant outcome (for example, according to the AHA, CVD rather than only CHD, since stroke and CHD share pathophysiological mechanisms [10, 97]), consists of predictors available in their situation and is developed or updated in a setting that closely resembles their own setting.

### Limitations

This study has several limitations. First, we focused on the three most validated and used prediction models in the USA, while in Europe many more prediction models are currently used for predicting cardiovascular risk, such as QRISK3 [22] and SCORE [21]. The differences between all these models are however limited, as most models include the same core set of predictors. Therefore, we believe our results can be generalized to other prediction models. Second, we had to rely on what is reported by the authors of primary validation studies and we unfortunately had to exclude relevant validations from our meta-analyses because of unreported information which we could not obtain from the authors. Only 19 out of 61 authors were able to provide us with additional information, and we had to exclude 9 validations for the c-statistic and 18 for the OE ratio. Sometimes, incomplete reporting made it difficult to judge whether a study should be included in our analyses, for example when there was no explicit reference to the validated model and when it was not clear whether changes were made to the model before validation. In one specific study (D’Agostino et al. 2001 [14]), we decided to include the study because the validated model was similar in predictors and outcome definition. Although its eligibility could be debated, the exclusion of this study would not alter our conclusions regarding the performance of the Framingham Wilson model. Due to poor reporting, many validations scored unclear risk of bias, especially for the domain predictors and outcome. Many other validations scored high risk of bias which may hamper our conclusions. Unfortunately, evidence on the impact of these issues on model performance is still limited [98], which makes it difficult to argue in which direction the predictive performance of the models will change if all validations had low risk of bias. Third, the total OE ratio, while commonly reported, only provides an overall measure of calibration. To overcome this problem, we extracted information on the OE ratio in categories of predicted risk, which showed there was more overestimation of risk in the highest categories of predicted risk. Based on this information, we calculated the calibration slope, which suggested that miscalibration of the Framingham Wilson and ATP III models and PCE men model was mostly related to heterogeneity in baseline risk, while for PCE women the model is overfitted or does not transport well to new populations. In addition, more clinically relevant measures, such as net benefit, could not be considered in this meta-analysis due to the lack of reporting of these measures [5]. Fourth, because of the low number of external validation studies, we did not perform meta-regression analyses for the ATP III model. Unfortunately, the relatively small sample size makes it difficult to draw firm conclusions on the sources of observed heterogeneity. Fifth, the exclusion of non-English studies could have influenced the geographical representation. However, since only one full-text article was excluded for this reason, we believe the effect on our results is limited.

## Conclusions

The Framingham Wilson, Framingham ATP III and PCE prediction models perform equally well in predicting the risk of CHD or CVD, but there is large variation between validations and only few direct comparisons have been performed so far. All three prediction models overestimate the risk of CHD or CVD if no local adjustments are made, which could lead to overtreatment in clinical practice. Therefore, we recommend that future studies should investigate reasons for overprediction and that guidelines offer advise how to make better use of existing models and subsequently tailor or recalibrate them to the setting at hand.

## Additional files

Additional file 1: Review question and description of prediction models. Review question according to PICOTS format and a description of the three prediction models included in this systematic review and meta-analysis. (DOCX 26 kb)

Additional file 2: Review protocol. (DOCX 56 kb)

Additional file 3: MOOSE checklist. Reporting checklist for systematic reviews. (DOC 56 kb)

Additional file 4: Search strategy. Overview of search terms and databases searched. (DOCX 16 kb)

Additional file 5: Items for data extraction and risk of bias assessment. Overview and description of items for which data have been extracted and description of how risk of bias has been assessed. (DOCX 18 kb)

Additional file 6: Formulas used to estimate missing quantitative information. Overview of formulas used to estimate missing information on performance measures. (DOCX 23 kb)

Additional file 7: Statistical analyses. Details on the statistical analyses. (DOCX 24 kb)

Additional file 8: Description of outcomes. Overview showing which outcome definitions have been excluded and which definitions have been included. (DOCX 49 kb)

Additional file 9: Cohorts used multiple times to validate the same model. Overview of cohorts that were used more than once to validate the same model and which validation was subsequently chosen for which reason. (DOCX 310 kb)

Additional file 10: Characteristics of included validations. Table with an overview of the characteristics of included validations. (DOCX 315 kb)

Additional file 11: Summary calibration slope. Table with results from the pooled calibration slope. (DOCX 16 kb)

Additional file 12: Sensitivity analyses. Table with results from sensitivity analyses. (DOCX 18 kb)

Additional file 13: Meta-regression analyses. Figures with results from meta-regression analyses. (DOCX 141 kb)

Additional file 14: Dataset. Dataset used for analyses. (CSV 166 kb)

## Abbreviations

AHA: American Heart Association
ATP: Adult Treatment Panel
CHD: Coronary heart disease
CI: Confidence interval
CVD: Cardiovascular disease
IQR: Interquartile range
OE ratio: Observed versus expected ratio
PCE: Pooled cohort equations
PI: Prediction interval
