# Supplementary material for: Performance of the Framingham risk models and pooled cohort equations for predicting 10-year risk of cardiovascular disease: a systematic review and meta-analysis
Source: BMC Med. 2019 Jun 13;17:109. doi: 10.1186/s12916-019-1340-7 (PMC6563379; doi:10.1186/s12916-019-1340-7)
Supplement: Supplementary file 1 — Review question and description of prediction models. Review question according to PICOTS format and a description of the three prediction models included in this systematic review and meta-analysis. (DOCX 26 kb) [file 12916_2019_1340_MOESM1_ESM.docx]

Additional file 1. Review question and description of prediction models.

Review question and PICOTS components

Review question: “What is the predictive performance of the Framingham Wilson, ATP III and PCE models in men and women separately for predicting 10-year risk of coronary heart disease (CHD) or cardiovascular disease (CVD) in the general population?”

*Patients* - General population, divided by gender. Include population based and primary care cohorts; exclude cohorts in which specific patient populations were excluded

*Intervention and Comparators* - Framingham Wilson 1998, Framingham ATP III 2003, PCE 2013, for men and women separately

*Outcome* - Outcome for which the original models were developed (fatal or nonfatal CHD for ATP III and Wilson, fatal or nonfatal CVD for PCE)

*Timing/prediction horizon* - 10 years

*Setting* - Primary care and public health

Overview of Framingham prediction models and PCE

|  | **Framingham Wilson [1]** | **Framingham ATP III [2, 3]** | **PCE [4]** |
| --- | --- | --- | --- |
| **Development cohort(s)** | - Framingham Heart Study: 11th examination of the original Framingham cohort or initial examination of the Framingham Offspring Study | - Framingham Heart Study | - Framingham Heart Study: original and offspring cohorts.  - Atherosclerosis Risk in Communities (ARIC) study  - Cardiovascular Health Study (CHS)  - Coronary Artery Risk Development in Young Adults (CARDIA) study |
| **In/exclusion criteria** | People aged 30 to 74 years old at the time of their Framingham Heart Study examination in 1971 to 1974. Persons with overt CHD at the baseline examination were excluded. | People aged 20 to 79 without diabetes. | People aged 40 to 79, apparently healthy, African American or White, and free of a previous history of MI (recognized or unrecognized), stroke, congestive heart failure, percutaneous coronary intervention, coronary bypass surgery, or atrial fibrillation. |
| **Predictors** | Age  Smoking  Diabetes  Systolic blood pressure  Diastolic blood pressure  Total or LDL cholesterol  HDL cholesterol | Age  Smoking  Systolic blood pressure  Treatment of blood pressure  Total cholesterol  HDL cholesterol | Age  Smoking  Diabetes  Systolic blood pressure  Treatment of blood pressure  Total cholesterol  HDL cholesterol |
| **Predicted outcome** | Fatal or nonfatal CHD, defined as angina pectoris, recognized and unrecognized myocardial infarction, coronary insufficiency, and coronary heart disease death. | Fatal or nonfatal CHD, defined as myocardial infarction or CHD death. | Atherosclerotic CVD defined as nonfatal myocardial infarction or coronary heart disease death, or fatal or nonfatal stroke. |
| **Prediction horizon** | 10 years | 10 years | 10 years |

**References**

1. Wilson PW, D'Agostino RB, Levy D, Belanger AM, Silbershatz H, Kannel WB. Prediction of coronary heart disease using risk factor categories. Circulation. 1998;97(18):1837-47. Epub 1998/05/29. PubMed PMID: 9603539.

2. Third Report of the National Cholesterol Education Program (NCEP) Expert Panel on Detection, Evaluation, and Treatment of High Blood Cholesterol in Adults (Adult Treatment Panel III) final report. Circulation. 2002;106(25):3143-421. Epub 2002/12/18. PubMed PMID: 12485966.

3. Executive Summary of The Third Report of The National Cholesterol Education Program (NCEP) Expert Panel on Detection, Evaluation, And Treatment of High Blood Cholesterol In Adults (Adult Treatment Panel III). JAMA. 2001;285(19):2486-97. Epub 2001/05/23. PubMed PMID: 11368702.

4. Goff DC, Jr., Lloyd-Jones DM, Bennett G, Coady S, D'Agostino RB, Gibbons R, et al. 2013 ACC/AHA guideline on the assessment of cardiovascular risk: a report of the American College of Cardiology/American Heart Association Task Force on Practice Guidelines. Circulation. 2014;129(25 Suppl 2):S49-73. Epub 2013/11/14. doi: 10.1161/01.cir.0000437741.48606.98. PubMed PMID: 24222018.
