# Supplementary material for: Performance of the Framingham risk models and pooled cohort equations for predicting 10-year risk of cardiovascular disease: a systematic review and meta-analysis
Source: BMC Med. 2019 Jun 13;17:109. doi: 10.1186/s12916-019-1340-7 (PMC6563379; doi:10.1186/s12916-019-1340-7)
Supplement: Supplementary file 2 — Review protocol. (DOCX 56 kb) [file 12916_2019_1340_MOESM2_ESM.docx]

Additional file 2. Review protocol

Protocol

**Performance of the Framingham risk models and Pooled Cohort Equations for predicting 10-year risk of cardiovascular disease: a systematic review and meta-analysis**

Background

The implementation of the Framingham prediction models is currently recommended in the United States to decide who should receive treatment to lower the risk of cardiovascular disease (CVD). This has led to a vast amount of external validation studies in which the performance of these models was investigated. Incorrect predictions can have a large impact on patients because it can lead to over- or undertreatment. It is however unknown which factors cause heterogeneity in model performance. By conducting a meta-analysis of model performance statistics, we can determine which factors have a large influence on model performance. This information can ultimately help us in identifying populations and outcomes for which these models can reliably be used and identify populations and outcomes for which further research is necessary to improve risk prediction.

Objectives

Paper 1:

- To investigate the range of performance of three Framingham risk equations (Wilson 1998, ATP III 2002 guideline and Pooled Cohort Equations), in validation studies in which the original model is applied as it is developed (no recalibration or refitting).
- To establish prediction intervals for performance when the model is applied in new populations or settings.
- To determine which factors influence model performance. Factors that we will investigate in this study all relate to the study population (eligibility criteria and case-mix).

**P**: general population (NOT: population for which model is developed)

**I/C**: Wilson, ATPIII, PCE

**O**: Outcome for which model has been developed (fatal or nonfatal CHD for ATP III and Wilson, fatal or nonfatal CVD for PCE)

**T**: 10 year (no selection based on time)

**S**: primary care or general population

Design

Systematic review and meta-analysis of external validation papers of the Framingham models.

Articles published before June 2013 can be selected from a previous review [1].

The database will be updated with papers published after June 2013 using a citation search (Appendix 1) and the search strategy used in the previous review [1]. Articles identified by both searches will be screened for eligibility on title and abstract by one reviewer (JD) (in doubt = inclusion), and subsequently on full text independent by two reviewers (JD will screen all papers, and RP and PH will screen half of the papers).

Reference lists of systematic reviews identified by our search will be screened for additional articles.

Selection criteria

Inclusion criteria:

- Prognostic studies
- Framingham model (Wilson, ATP III, PCE)
- External validation papers
- Model aimed for individual risk predictions
- Original model is validated (i.e. Original beta-coefficients baseline hazard)
- Predicted outcome: CHD or CVD
- Primary prevention
- Domain: general population or primary care setting

Exclusion criteria:

- No validation of individual outcomes (e.g. cross-sectional study with link to population statistics)
- Not human
- Comments, editorials, conference abstracts etc.
- SRs: to identify additional references
- Patient populations
- Impact studies
- Methodological studies
- Predictor finding studies
- Model updated before validation, e.g. intercept recalibration, slope recalibration, refitting, etc.

Note: If c-statistic or calibration is not reported, we still include this paper.

Note 2: if a model is first validated as it was originally developed, and subsequently recalibrated, the results of that first validation will be included.

If the model is only validated for men and women together (instead of stratified analyses) these papers will be excluded during data-extraction.

If one model is validated multiple times in the same cohort, this validation will be included in the qualitative synthesis, and excluded in the meta-analysis.

Data extraction

Data extraction will be done by one reviewer (JD or RP), risk of bias will be assessed independent by two reviewers (JD and RP) and discrepancies will be discussed. Data extraction will be based on PROBAST, TRIPOD and CHARMS. At the end of this file a list is included (Appendix 2).

Statistical analyses

Analyses will be split up by gender and by model, so six groups in total.

Pooled c-statistics and OE ratios will be calculated by accounting for the presence of between-study heterogeneity. This can be achieved by adopting regression models with random-effects. We will pool the logit c-statistic and log OE ratio. The meta-analysis will be performed in R, with the metafor package using the rma function with Paule-Mandel estimator and Hartung-Knapp-Sidik-Jonkman method for calculating 95% confidence intervals.[2] 95% prediction intervals will be calculated to determine what performance can be expected in new studies with predefined characteristics.

*Restore missing information*

Missing standard errors of c-statistic will be calculated using formulas by Hanley and Newcombe [3, 4]. Formulas to restore missing standard errors for OE ratio will be developed in parallel with this project and published as part of a general guidance paper on systematic review and meta-analysis of prediction model studies.

OE ratios are highly sensitive to duration of follow-up. We will explore ways to account for this.

*Calibration in risk groups*

During data extraction, we register whether information on calibration in risk groups is reported (e.g. in calibration plots or tables). If there is a sufficient number of studies reporting this information, exact numbers will be extracted and visualized in a calibration plot.

*Sensitivity analyses*

We will perform three sensitivity analyses:

- Exclude studies with high risk of bias for at least one domain

- Weigh studies by the inverse of the sample size (rather than inverse of the variance)

- Bivariate meta-analysis to jointly pool c-statistic and OE ratio

*Metaregression*

C-statistic and OE ratio depend on casemix (e.g. see Vergouwe 2010). More heterogeneity in casemix leads to a higher c-statistic because it is easier to distinguish between people who develop and who do not develop the disease. We have to correct for this. Therefore we extract information on every predictor in the model from every validation study. For categorical variable (like smoking) we extract the percentages for every category and for continuous variables we extract the mean and the sd (if not reported we extract other information like the median, range or interquartile range).

Using meta-regression we will explore the association between these case-mix variables and c-statistic and OE ratio. Furthermore, it will be explored whether eligibility criteria (ie exclusion of people with previous CVD, diabetes, and people receiving treatment that lowers the risk of CVD) are associated with variation in these performance measures. Other ways to visualize heterogeneity in eligibility criteria will be explored.

*Publication bias*

Publication bias will not be assessed as methods for this in systematic reviews of prognosis studies are not available yet.

**Appendix 1: Citation search**

Web of Science and Scopus will be searched for studies citing the following references:

*Wilson:*

- Wilson PW, D'Agostino RB, Levy D, Belanger AM, Silbershatz H, Kannel WB. Prediction of coronary heart disease using risk factor categories. *Circulation* 1998;97(18):1837-47.

*ATP III:*

- Third Report of the National Cholesterol Education Program (NCEP) Expert Panel on Detection, Evaluation, and Treatment of High Blood Cholesterol in Adults (Adult Treatment Panel III) final report. *Circulation* 2002;106(25):3143-421.

- Executive Summary of The Third Report of The National Cholesterol Education Program (NCEP) Expert Panel on Detection, Evaluation, And Treatment of High Blood Cholesterol In Adults (Adult Treatment Panel III). *JAMA* 2001;285(19):2486-97.

*PCE:*

- Goff DC, Jr., Lloyd-Jones DM, Bennett G, et al. 2013 ACC/AHA guideline on the assessment of cardiovascular risk: a report of the American College of Cardiology/American Heart Association Task Force on Practice Guidelines. *Circulation* 2014;129(25 Suppl 2):S49-73.

- Goff DC, Jr., Lloyd-Jones DM, Bennett G, et al. 2013 ACC/AHA guideline on the assessment of cardiovascular risk: a report of the American College of Cardiology/American Heart Association Task Force on Practice Guidelines. *J Am Coll Cardiol* 2014;63(25 Pt B):2935-59

**Appendix 2: Data extraction table**

| **General items** |  |
| --- | --- |
| Author |  |
| Year |  |
| Journal |  |
| ID |  |
| Reviewer |  |
| Applied version of Framingham | If race is not specified, enter "PCE men"/"PCE women"/"PCE men and women". |
| Type of study | Predesigned validation study: study is prospectively designed with the aim to validate the model |
| **Participant selection** |  |
| Study design |  |
|  | Comment on study design |
| *In- and exclusion criteria for the analyses* |  |
| Lower age limit | Enter number |
| Upper age limit | Enter number |
| Free from CVD | Only information on eligibility criteria for CVD |
| Free from other disease | Only information on eligibility criteria for diseases other than CVD |
| Treatment |  |
| Having disease |  |
| Race |  |
| Other | E.g. information on completeness of follow-up or predictors. |
| In case of RCT - which arm is used? |  |
| In case of RCT - which treatment(s) was/were studied? |  |
| *Casemix* | ***For continuous variables: if reported extract mean and SD (other information is not needed), if these are not reported, extract median and IQR. If these are not reported specify any other informationthat is reported (e.g. a plot).*** |
| Is casemix solely reported for 2 separate groups (e.g. for cases and noncases)? | ***If yes, extract numbers at the bottom of this DE table.  If casemix is reported for men and women together (but validation is performed seperately) add a third column to extract numbers. Also: if casemix is reported for men and women seperately and together, extract all information. We might use this for some sort of imputation.*** |
| Is casemix also reported for subgroups (e.g. risk groups, age groups etc) in addition to overall casemix details? |  |
|  | If yes, specify which subgroups |
| Age | Mean |
|  | SD |
|  | Median |
|  | IQR - 25th percentile |
|  | IQR - 75th percentile |
|  | If NR: other (specify) |
| Gender | % men |
| Smoking | % Never |
|  | % Past |
|  | % Current |
|  | % Ever |
|  | % not specified/other (specify) |
| Diabetes | % |
| Treated hypertension / use of antihypertensives | % |
| Use of lipid lowering medication | % |
| Hypertension | % |
| Hypercholesterolaemia | % |
| SBP | Mean |
|  | SD |
|  | Median |
|  | IQR - 25th percentile |
|  | IQR - 75th percentile |
|  | If NR: other (specify) |
| Treated SBP | Mean |
|  | SD |
|  | Median |
|  | IQR - 25th percentile |
|  | IQR - 75th percentile |
|  | If NR: other (specify) |
| Untreated SBP | Mean |
|  | SD |
|  | Median |
|  | IQR - 25th percentile |
|  | IQR - 75th percentile |
|  | If NR: other (specify) |
| DBP | Mean |
|  | SD |
|  | Median |
|  | IQR - 25th percentile |
|  | IQR - 75th percentile |
|  | If NR: other (specify) |
| LDL-C | Unity (e.g. mg/dl) |
|  | Mean |
|  | SD |
|  | Median |
|  | IQR - 25th percentile |
|  | IQR - 75th percentile |
|  | If NR: other (specify) |
| HDL-C | Unit (e.g. mg/dl) |
|  | Mean |
|  | SD |
|  | Median |
|  | IQR - 25th percentile |
|  | IQR - 75th percentile |
|  | If NR: other (specify) |
| Total cholesterol | Unit (e.g. mg/dl) |
|  | Mean |
|  | SD |
|  | Median |
|  | IQR - 25th percentile |
|  | IQR - 75th percentile |
|  | If NR: other (specify) |
| Linear predictor | Specify which information is reported e.g. average predicted risk, number of points etc. (so, information on predicted and not on observed) |
|  | Mean |
|  | SD |
|  | Median |
|  | IQR - 25th percentile |
|  | IQR - 75th percentile |
|  | If NR: other (specify) |
| Race |  |
| Any other treatment information |  |
| *Study dates* |  |
| Start date recruitment period (dd-mm-yyyy) | If day is not reported enter 00. So July 2010 is 00-07-2010 |
| End date recruitment period (dd-mm-yyyy) |  |
| End date of follow up (dd-mm-yyyy) |  |
| Follow-up time - median (years) | If follow-up is reported in months, just divide it by 12. If only max is reported: NR-10 |
| Follow-up time - range (years) min |  |
| Follow-up time - range (years) max |  |
| Follow-up time - other information (specify) |  |
| Prediction horizon (years) |  |
| *Location* |  |
| Number of centers |  |
| Location of centers - continent |  |
| Location of centers - country | Exception: Framingham. If model is validated in the Framingham heart study, enter Framingham instead of United States. |
| **Risk of bias introduced by selection of participants** | High / low / unclear |
| Justification of bias rating | Justification is not always necessary when you score LOW (although it might be helpful), but ís necessary when you score HIGH or UNCLEAR. |
| **Concern that the included participants and setting do not match the review question** | High / low / unclear  Studies might have reduced applicability to our review if they included a study population different from the original development study, e.g. if they included only young people (see seperate file). |
| Justification of applicability rating | Justification is not always necessary when you score LOW (although it might be helpful), but ís necessary when you score HIGH or UNCLEAR. |
| **Predictors** |  |
| Actions to blind assessment of predictors for the outcome |  |
| Actions to blind assessment of predictors for each other |  |
| Was there a general statement that predictor definitions were the same as in the development study? If not, answer the following question for every predictor. |  |
| For the following predictors: was the same definition used? If not, copy the definition in the box below. (if the same definition is used, you don't have to copy it) |  |
| Smoking Wilson: Persons who smoked regularly during the previous 12 months ATPIII: Any cigarette smoking in the past month PCE: current smoker | Yes/No/NR/NA |
|  | Definition |
| Diabetes Wilson: under treatment with insulin or oral hypoglycemic agents, if casual blood glucose determinations exceeded 150 mg/dL at two clinic visits in the original cohort, or if fasting blood glucose exceeded 140 mg/dL at the initial examination of the Offspring Study participants. ATPIII: NA. Not in model PCE: no definition reported | Yes/No/NR/NA/definition of original model not reported Score NA if predictor was not included in the model. Score 'Definition of original model not reported' in case of PCE |
|  | Definition (also extract this if the definition of the original model was not reported). |
| Treatment of blood pressure Wilson: not included in model ATPIII: taking antihypertensive medications PCE: no definition reported | Yes/No/NR/NA/definition of original model not reported Score NA if predictor was not included in the model. Score 'Definition of original model not reported' in case of PCE |
|  | Definition (also extract this if the definition of the original model was not reported). |
| Systolic blood pressure Wilson: Two blood pressure determinations were made after the participant had been sitting at least 5 minutes, and the average was used for analyses.  ATPIII: The average of several blood pressure measurements, as recommended by JNC VI, is needed for an accurate measure of baseline blood pressure. PCE: no definition reported | Yes/No/NR/NA/definition of original model not reported Score NA if predictor was not included in the model. Score 'Definition of original model not reported' in case of PCE |
|  | Definition (also extract this if the definition of the original model was not reported). |
| Diastolic blood pressure Wilson: Two blood pressure determinations were made after the participant had been sitting at least 5 minutes, and the average was used for analyses.  ATPIII: NA. Not in model. PCE: NA. Not in model | Yes/No/NR/NA |
|  | Definition |
| Hypertension Wilson: Hypertension was categorized according to blood pressure readings by JNC-V definitions: optimal (systolic <120 mm Hg and diastolic <80 mm Hg), normal blood pressure (systolic 120 to 129 mm Hg or diastolic 80 to 84 mm Hg), high normal blood pressure (systolic 130 to 139 mm Hg or diastolic 85 to 89 mm Hg), hypertension stage I (systolic 140 to 159 mm Hg or diastolic 90 to 99 mm Hg), and hypertension stage II–IV (systolic >160 or diastolic >100 mm Hg). When systolic and diastolic pressures fell into different categories, the higher category was selected for the purposes of classification. Blood pressure categorization was made without regard to the use of antihypertensive medication. ATPIII: NA. not in model. PCE: NA. not in model. | Yes/No/NR/NA |
|  | Definition |
| LDL Cholesterol Wilson: Blood was drawn at the baseline examination after an overnight fast, and EDTA plasma was used for all cholesterol and triglyceride measurements. When triglycerides were <400 mg/dL, the concentration of LDL-C was estimated indirectly by use of the Friedewald formula; for triglycerides>400 mg/dL, the LDL-C was estimated directly after ultracentrifugation of plasma and measurement of cholesterol in the bottom fraction (plasma density<1.006). Categories: (<130, 130 to 159, and >160 mg/dL). ATPIII: NA. Not in model. PCE: NA. Not in model | Yes/No/NR/NA |
|  | Definition |
| HDL cholesterol Wilson: Blood was drawn at the baseline examination after an overnight fast, and EDTA plasma was used for all cholesterol and triglyceride measurements. HDL-C was measured after precipitation of VLDL and LDL proteins with heparinmagnesium according to the Lipid Research Clinics Program protocol. Categories: (<35, 35 to 59, and >60 mg/dL) ATPIII: Assumed same laboratory technique as Wilson. Total cholesterol and HDL-cholesterol values should be the average of at least two measurements obtained from lipoprotein analysis. Low HDL: HDL cholesterol <40 mg/dL. PCE: No definition reported. | Yes/No/NR/NA/definition of original model not reported Score NA if predictor was not included in the model. Score 'Definition of original model not reported' in case of PCE |
|  | Definition (also extract this if the definition of the original model was not reported). |
| Total cholesterol Wilson: Blood was drawn at the baseline examination after an overnight fast, and EDTA plasma was used for all cholesterol and triglyceride measurements. Cholesterol was determined according to the Abell-Kendall technique. Categories: (<200, 200 to 239, 240 to 279, and >280 mg/dL) ATPIII: Assumed same laboratory technique as Wilson. Total cholesterol and HDL-cholesterol values should be the average of at least two measurements obtained from lipoprotein analysis.  PCE: No definition reported. | Yes/No/NR/NA/definition of original model not reported Score NA if predictor was not included in the model. Score 'Definition of original model not reported' in case of PCE |
|  | Definition (also extract this if the definition of the original model was not reported). |
| Were predictors deleted? |  |
| If yes, which ones? |  |
| **Risk of bias introduced by predictors or their assessment** | High / low / unclear |
| Justification of bias rating: | Justification is not always necessary when you score LOW (although it might be helpful), but ís necessary when you score HIGH or UNCLEAR. |
| **Concern that the definition, assessment or timing of assessment of predictors in the model do not match the review question** | High / low / unclear |
| Justification of applicability rating | Justification is not always necessary when you score LOW (although it might be helpful), but ís necessary when you score HIGH or UNCLEAR. |
| **Outcome** |  |
| Is the outcome definition the same as the development study? Wilson: CHD: angina pectoris, recognized and unrecognized myocardial infarction, coronary insufficiency, and coronary heart disease death. ATPIII: myocardial infarction + CHD death PCE: first ASCVD event, defined as nonfatal myocardial infarction or coronary heart disease death, or fatal or nonfatal stroke. |  |
| Outcome - main category *CHD: heart disease CVD: heart and brain disease* |  |
| Outcome - full definition | Copy/paste information |
| Outcome - measurement method | E.g. expert panel, death register |
| Outcome - ICD-codes | Copy/paste information |
| If a composite outcome was used, enter the relative or absolute frequency/distribution of each contributing outcome | Format: outcome number, outcome number. E.g. MI 250, stroke 302 |
| Actions to blind outcome assessment for the predictors | High / low / unclear |
| **Risk of bias introduced by the outcome or its determination** |  |
| Justification of bias rating | Justification is not always necessary when you score LOW (although it might be helpful), but ís necessary when you score HIGH or UNCLEAR. |
| **Concern that the outcome, its definition, timing or determination do not match the review question** | High / low / unclear  You might score HIGH if outcome definition does not match original definition (see above for original definitions) |
| Justification of applicability rating | Justification is not always necessary when you score LOW (although it might be helpful), but ís necessary when you score HIGH or UNCLEAR. |
| **Sample size and participant flow** |  |
| Number of participants included in the full cohort |  |
| Number of events in the full cohort |  |
| Number of participants included in the analysis | Enter number |
| Number of events included in the analysis | Enter number |
| Number of participants with any missing value | Enter number |
| Number of participants with missing data for outcome | Enter number |
| Number of participants with missing data for predictors | Enter number |
| Number of participants with missing data for each predictor: Default is NR because we expect it is often not reported. |  |
| Age | Enter number |
| Smoking | Enter number |
| Diabetes | Enter number |
| Treatment of blood pressure | Enter number |
| Hypertension | Enter number |
| Systolic blood pressure | Enter number |
| Diastolic blood pressure | Enter number |
| LDL cholesterol | Enter number |
| HDL cholesterol | Enter number |
| Total cholesterol | Enter number |
| Method used to account for missing data |  |
|  | Comment on missing data |
| **Risk of bias introduced by sample size or participant flow** | High / low / unclear  Score UNCLEAR if there is no information on missing data, but you might score LOW if there was no information on missing data, but dedicated data collection. |
| Justification of bias rating | Justification is not always necessary when you score LOW (although it might be helpful), but ís necessary when you score HIGH or UNCLEAR. |
| **Analysis** |  |
| How were predictors calculated |  |
|  | Comment on calculating predictors |
| Was the model also updated? | D'Agostino method (2001): taking beta's and baseline hazard/incidence rate of original model but standardize them with mean values from the validation cohort. |
|  | Comment on model updating |
| Type of validation - Geographical Is this a validation on a different geographical location or in a different cohort? *Wilson: developed on Framingham Heart Study ATPIII: developed on Framingham Heart Study PCE: developed on Framingham heart study, Framingham offspring cohort, ARIC study, Cardiovascular Health Study (CHS), CARDIA study.* | Score Yes if the validation is performed in a cohort other than the development cohort(s). |
| Type of validation - Temporal Is this a validation in a different time period? (i.e. no overlap in inclusion dates) *Wilson: inclusion between 1971-1974 ATPIII: inclusion between 1971-1974 PCE: inclusion between 1971-1974, 1984-1993* | Score Yes if there is no overlap in inclusion dates. |
| Type of validation - Domain/setting Is this a validation in a different domain or setting? | Default is no. You should only score Yes if there are large differences in eligibility criteria, e.g. the model was validated in a secondary care population. if they say we excluded participants with diabetes or with high cholesterol levels, this is not a problem. If you score Yes, we might have to discuss the paper for possible exclusion. |
| Type of validation - Investigators Is this a validation by different investigators? Is there NO overlap between the researchers of the validation study and the development study? | Score YES if there was NO overlap, score NO if there wás overlap between authors. |
| Type of validation - Other (specify) |  |
| **Risk of bias introduced by the analysis** | High / low / unclear |
| Justification of bias rating | Justification is not always necessary when you score LOW (although it might be helpful), but ís necessary when you score HIGH or UNCLEAR. |
| **Results** |  |
| Comparison of distribution of predictors for development and validation dataset (Reference is not sufficient) |  |
| *Performance before updating* | If performance is reported for men and women seperately and together, enter statistics for men and women together in a third column |
| C-statistic - type | Add NA |
| C-statistic |  |
| C-statistic - 95% CI Lower bound |  |
| C-statistic - 95% CI Upper bound |  |
| C-statistic - SE |  |
| C-statistic - other information | Specify |
| Observed rate | % |
| Observed rate - 95% CI Lower bound |  |
| Observed rate - 95% CI Upper bound |  |
| Expected rate | % |
| Expected rate - 95% CI Lower bound |  |
| Expected rate - 95% CI Upper bound |  |
| Observed/expected |  |
| Observed/expected - 95% CI Lower bound |  |
| Observed/expected - 95% CI Upper bound |  |
| Observed/expected - SE |  |
| Observed/expected - p-value |  |
| Observed/expected - IQR Lower bound |  |
| Observed/expected - IQR Upper bound |  |
| Expected/observed |  |
| Expected/observed - 95% CI Lower bound |  |
| Expected/observed - 95% CI Upper bound |  |
| Expected/observed - SE |  |
| Expected/observed - p-value |  |
| Expected/observed - IQR Lower bound |  |
| Expected/observed - IQR Upper bound |  |
| Observed/expected - other information | Specify any other form in which information on calibration is reported, e.g. a calibration table or plot. |
| *Performance after updating - if applicable* | If performance is reported for men and women seperately and together, enter statistics for men and women together in a third column |
| C-statistic - type |  |
| C-statistic |  |
| C-statistic - 95% CI Lower bound |  |
| C-statistic - 95% CI Upper bound |  |
| C-statistic - SE |  |
| C-statistic - other information | Specify |
| Observed rate | % |
| Observed rate - 95% CI Lower bound |  |
| Observed rate - 95% CI Upper bound |  |
| Expected rate | % |
| Expected rate - 95% CI Lower bound |  |
| Expected rate - 95% CI Upper bound |  |
| Observed/expected |  |
| Observed/expected - 95% CI Lower bound |  |
| Observed/expected - 95% CI Upper bound |  |
| Observed/expected - SE |  |
| Observed/expected - p-value |  |
| Observed/expected - IQR Lower bound |  |
| Observed/expected - IQR Upper bound |  |
| Expected/observed |  |
| Expected/observed - 95% CI Lower bound |  |
| Expected/observed - 95% CI Upper bound |  |
| Expected/observed - SE |  |
| Expected/observed - p-value |  |
| Expected/observed - IQR Lower bound |  |
| Expected/observed - IQR Upper bound |  |
| Observed/expected - other information | Specify any other form in which information on calibration is reported, e.g. a calibration table or plot. |
| *For incremental value studies:* |  |
| Which predictor(s) is/are added to the model? | If there are a lot of predictors added (e.g. 27 SNPs are seperately added) it is okay to just state "27 SNPs". |
| How many incremental values were assessed? | e.g. if they performed seperate analyses adding CRP, IMT and CRP+IMT the answer is 3. |
| C-statistic after adding predictor | Enter the range of c-statistics if multiple incremental values were assessed. |

**References**

1. Damen JA, Hooft L, Schuit E, Debray TP, Collins GS, Tzoulaki I, et al. Prediction models for cardiovascular disease risk in the general population: systematic review. BMJ. 2016;353:i2416. Epub 2016/05/18. doi: 10.1136/bmj.i2416. PubMed PMID: 27184143; PubMed Central PMCID: PMCPmc4868251.

2. IntHout J, Ioannidis JP, Borm GF. The Hartung-Knapp-Sidik-Jonkman method for random effects meta-analysis is straightforward and considerably outperforms the standard DerSimonian-Laird method. BMC Med Res Methodol. 2014;14:25. Epub 2014/02/20. doi: 10.1186/1471-2288-14-25. PubMed PMID: 24548571; PubMed Central PMCID: PMCPmc4015721.

3. Hanley JA, McNeil BJ. The meaning and use of the area under a receiver operating characteristic (ROC) curve. Radiology. 1982;143(1):29-36. Epub 1982/04/01. doi: 10.1148/radiology.143.1.7063747. PubMed PMID: 7063747.

4. Newcombe RG. Confidence intervals for an effect size measure based on the Mann-Whitney statistic. Part 2: asymptotic methods and evaluation. Stat Med. 2006;25(4):559-73. Epub 2005/10/12. doi: 10.1002/sim.2324. PubMed PMID: 16217835.
