# Supplementary material for: Performance of the Framingham risk models and pooled cohort equations for predicting 10-year risk of cardiovascular disease: a systematic review and meta-analysis
Source: BMC Med. 2019 Jun 13;17:109. doi: 10.1186/s12916-019-1340-7 (PMC6563379; doi:10.1186/s12916-019-1340-7)
Supplement: Supplementary file 3 — MOOSE checklist. Reporting checklist for systematic reviews. (DOC 56 kb) [file 12916_2019_1340_MOESM3_ESM.doc]

**MOOSE Statement - Reporting Checklist for Authors, Editors, and Reviewers of Meta-analyses of Observational Studies**

| **Reporting Criteria** | **Reported in section** |
| --- | --- |
| **Reporting of Background** |  |
| Problem definition | Introduction, all paragraphs |
| Hypothesis statement | Introduction, paragraph 2 |
| Description of Study Outcome(s) | Introduction, paragraph 4, Additional file (AF) 1 |
| Type of exposure or intervention used | Introduction, paragraph 4, AF 1 |
| Type of study design used | Methods ‘Eligibility criteria’, AF 1 |
| Study population | Methods ‘Eligibility criteria’, AF 1 |
| **Reporting of Search Strategy** |  |
| Qualifications of searchers (eg, librarians and investigators) | Methods ‘Search and selection’ |
| Search strategy, including time period included in the synthesis and keywords | Methods ‘Search and selection’, AF 4 |
| Effort to include all available studies, including contact with authors | Methods ‘Search and selection’ |
| Databases and registries searched | Methods ‘Search and selection’, AF 4 |
| Search software used, name and version, including special features used (eg, explosion) | Methods ‘Search and selection’, AF 4 |
| Use of hand searching (eg, reference lists of obtained articles) | Methods ‘Search and selection’, AF 4 |
| List of citations located and those excluded, including justification | Not provided, >18000 citations were identified |
| Method for addressing articles published in languages other than English | Methods ‘Eligibility criteria’. |
| Method of handling abstracts and unpublished studies | Methods ‘Eligibility criteria’. |
| Description of any contact with authors | Methods ‘Data extraction and critical appraisal’ |
| **Reporting of Methods** |  |
| Description of relevance or appropriateness of studies assembled for assessing the hypothesis to be tested | Methods ‘Search and selection’ and ‘Eligibility criteria’ |
| Rationale for the selection and coding of data (eg, sound clinical principles or convenience) | Methods ‘Data extraction and critical appraisal’ |
| Documentation of how data were classified and coded (eg, multiple raters, blinding, and interrater reliability) | Methods ‘Data extraction and critical appraisal’, AF 5 |
| Assessment of confounding (eg, comparability of cases and controls in studies where appropriate | Not applicable |
| Assessment of study quality, including blinding of quality assessors; stratification or regression on possible predictors of study results | Methods ‘Data extraction and critical appraisal’ and ‘Statistical analyses’, AF 7 |
| Assessment of heterogeneity | Methods ‘Statistical analyses’, AF 7 |
| Description of statistical methods (eg, complete description of fixed or random effects models, justification of whether the chosen models account for predictors of study results, dose-response models, or cumulative meta-analysis) in sufficient detail to be replicated | Methods ‘Statistical analyses’, AF 7 |
| Provision of appropriate tables and graphics | Results |
| **Reporting of Results** |  |
| Table giving descriptive information for each study included | AF 10 |
| Results of sensitivity testing (eg, subgroup analysis) | AF 12 |
| Indication of statistical uncertainty of findings | All figures |
| **Reporting of Discussion** |  |
| Quantitative assessment of bias (eg, publication bias) | Discussion ‘Limitations’ |
| Justification for exclusion (eg, exclusion of non–English-language citations) | Discussion ‘Limitations’ |
| Assessment of quality of included studies | Discussion ‘Limitations’ |
| **Reporting of Conclusions** |  |
| Consideration of alternative explanations for observed results | Discussion ‘Reasons for overprediction’ |
| Generalization of the conclusions (ie, appropriate for the data presented and within the domain of the literature review) | Discussion ‘Limitations’ |
| Guidelines for future research | Discussion ‘Implications for practice and research’ |
| Disclosure of funding source | Funding; Competing interests |
