# Supplementary material for: Performance of the Framingham risk models and pooled cohort equations for predicting 10-year risk of cardiovascular disease: a systematic review and meta-analysis
Source: BMC Med. 2019 Jun 13;17:109. doi: 10.1186/s12916-019-1340-7 (PMC6563379; doi:10.1186/s12916-019-1340-7)
Supplement: Supplementary file 4 — Search strategy. Overview of search terms and databases searched. (DOCX 16 kb) [file 12916_2019_1340_MOESM4_ESM.docx]

Additional file 4. Search strategy

**MEDLINE search strategy**

1 chd risk assessment$.mp.

2 cvd risk assessment$.mp.

3 heart disease risk assessment$.mp.

4 coronary disease risk assessment$.mp.

5 cardiovascular disease risk assessment$.mp.

6 cardiovascular risk assessment$.mp.

7 cv risk assessment$.mp.

8 cardiovascular disease$ risk assessment$.mp.

9 coronary risk assessment$.mp.

10 coronary risk scor$.mp.

11 heart disease risk scor$.mp.

12 chd risk scor$.mp.

13 cardiovascular risk scor$.mp.

14 cardiovascular disease$ risk scor$.mp.

15 cvd risk scor$.mp.

16 cv risk scor$.mp.

17 or/1-16

18 cardiovascular diseases/

19 coronary disease/

20 cardiovascular disease$.mp.

21 heart disease$.mp.

22 coronary disease$.mp.

23 cardiovascular risk?.mp.

24 coronary risk?.mp.

25 exp hypertension/

26 exp hyperlipidemia/

27 or/18-26

28 risk function.mp.

29 Risk Assessment/mt

30 risk functions.mp.

31 risk equation$.mp.

32 risk chart?.mp.

33 (risk adj3 tool$).mp.

34 risk assessment function?.mp.

35 risk assessor.mp.

36 risk appraisal$.mp.

37 risk calculation$.mp.

38 risk calculator$.mp.

39 risk factor$ calculator$.mp.

40 risk factor$ calculation$.mp.

41 risk engine$.mp.

42 risk equation$.mp.

43 risk table$.mp.

44 risk threshold$.mp.

45 risk disc?.mp.

46 risk disk?.mp.

47 risk scoring method?.mp.

48 scoring scheme?.mp.

49 risk scoring system?.mp.

50 risk prediction?.mp.

51 predictive instrument?.mp.

52 project$ risk?.mp.

53 cdss.mp.

54 or/28-53

55 27 and 54

56 17 or 55

57 new zealand chart$.mp.

58 sheffield table$.mp.

59 procam.mp.

60 General Rule to Enable Atheroma Treatment.mp.

61 dundee guideline$.mp.

62 shaper scor$.mp.

63 (brhs adj3 score$).mp.

64 (brhs adj3 risk$).mp.

65 copenhagen risk.mp.

66 precard.mp.

67 (framingham adj1 (function or functions)).mp.

68 (framingham adj2 risk).mp.

69 framingham equation.mp.

70 framingham model$.mp.

71 (busselton adj2 risk$).mp.

72 (busselton adj2 score$).mp.

73 erica risk score$.mp.

74 framingham scor$.mp.

75 dundee scor$.mp.

76 brhs scor$.mp.

77 British Regional Heart study risk scor$.mp.

78 brhs risk scor$.mp.

79 dundee risk scor$.mp.

80 framingham guideline$.mp.

81 framingham risk?.mp.

82 new zealand table$.mp.

83 ncep guideline?.mp.

84 smac guideline?.mp.

85 copenhagen risk?.mp.

86 or/57-85

87 56 or 86

88 exp decision support techniques/

89 Diagnosis, Computer-Assisted/

90 Decision Support Systems,Clinical/

91 algorithms/

92 algorithm?.mp.

93 algorythm?.mp.

94 decision support?.mp.

95 predictive model?.mp.

96 treatment decision?.mp.

97 scoring method$.mp.

98 (prediction$ adj3 method$).mp.

99 or/88-98

100 Risk Factors/

101 exp Risk Assessment/

102 (risk? adj1 assess$).mp.

103 risk factor?.mp.

104 or/100-103

105 27 and 99 and 104

106 87 or 105

107 stroke.mp.

108 exp Stroke/

109 cerebrovascular.mp. or exp Cerebrovascular Circulation/

110 limit 106 to ed=20040101-20130601

111 107 or 108 or 109

112 111 and 54

113 111 and 99 and 104

114 112 or 113

115 106 or 114

Citation search

Web of Science and Scopus were searched for studies citing the following references:

*Wilson:*

- Wilson PW, D'Agostino RB, Levy D, Belanger AM, Silbershatz H, Kannel WB. Prediction of coronary heart disease using risk factor categories. *Circulation* 1998;97(18):1837-47.

*ATP III:*

- Third Report of the National Cholesterol Education Program (NCEP) Expert Panel on Detection, Evaluation, and Treatment of High Blood Cholesterol in Adults (Adult Treatment Panel III) final report. *Circulation* 2002;106(25):3143-421.

- Executive Summary of The Third Report of The National Cholesterol Education Program (NCEP) Expert Panel on Detection, Evaluation, And Treatment of High Blood Cholesterol In Adults (Adult Treatment Panel III). *JAMA* 2001;285(19):2486-97.

*PCE:*

- Goff DC, Jr., Lloyd-Jones DM, Bennett G, et al. 2013 ACC/AHA guideline on the assessment of cardiovascular risk: a report of the American College of Cardiology/American Heart Association Task Force on Practice Guidelines. *Circulation* 2014;129(25 Suppl 2):S49-73.

- Goff DC, Jr., Lloyd-Jones DM, Bennett G, et al. 2013 ACC/AHA guideline on the assessment of cardiovascular risk: a report of the American College of Cardiology/American Heart Association Task Force on Practice Guidelines. *J Am Coll Cardiol* 2014;63(25 Pt B):2935-59
