# Supplementary material for: Performance of the Framingham risk models and pooled cohort equations for predicting 10-year risk of cardiovascular disease: a systematic review and meta-analysis
Source: BMC Med. 2019 Jun 13;17:109. doi: 10.1186/s12916-019-1340-7 (PMC6563379; doi:10.1186/s12916-019-1340-7)
Supplement: Supplementary file 5 — Items for data extraction and risk of bias assessment. Overview and description of items for which data have been extracted and description of how risk of bias has been assessed. (DOCX 18 kb) [file 12916_2019_1340_MOESM5_ESM.docx]

Additional file 5. Items for data extraction and risk of bias assessment

List of items for which data were extracted.

| **Item** | **Description / examples** |
| --- | --- |
| Validated model | Framingham Wilson, Framingham ATPIII, PCE; men or women; race (PCE); LDL or total cholesterol (Framingham Wilson). |
| Study type | Only external validation; external validation and development of a new model; external validation and incremental value assessment. |
| Study design | Cohort, randomized controlled trial |
| Eligibility criteria for participants | Age, (exclusion of) comorbidities, treatment, race. |
| Study dates | Inclusion dates, end of follow-up, follow-up time. |
| Prediction horizon | Time period for which predictions were made, e.g. 10 years. |
| Geographical location | Country and continent. |
| Case-mix | Information on the frequency, or mean/median and spread of the following population characteristics of the validation study: age, gender, smoking, diabetes, treatment, hypertension, systolic blood pressure, diastolic blood pressure, total cholesterol, LDL cholesterol, HDL cholesterol, race, other diseases, linear predictor, 10-year predicted survival probability. |
| Predictors | Full definition, measurement method, blinding of measurements. |
| Predicted outcome | Full definition, including ICD-codes. |
| Sample size | Number of participants, number of events, Kaplan-Meier 10-year survival probability. |
| Performance | C-statistic, 10-year total observed/expected ratio, standard error, 95% confidence intervals, calibration plot, calibration table. Performance of the original model and after updating the model were extracted. |

List of domains and signaling questions used for risk of bias assessment.

| **Domain** | **Signaling question** |
| --- | --- |
| Participant selection | 1. Were appropriate data sources used, e.g. cohort, RCT or nested case-control study data? |
|  | 2. Were all inclusions and exclusions based on characteristics of participants appropriate (e.g. comorbidities, treatment)? |
| Predictors | 1. Were predictors defined and assessed in a similar way for all participants? |
|  | 2. Were predictor assessments made without knowledge of outcome data? |
|  | 3. Are all predictors available at the time the model is used? |
|  | 4. Were predictors defined and assessed in the same way as in the original Framingham model? |
| Outcome | 1. Was a pre-specified outcome definition used? |
|  | 2. Were predictors excluded from the outcome definition? |
|  | 3. Was the outcome defined and determined in a similar way for all participants? |
|  | 4. Was the outcome determined without knowledge of predictor information? |
|  | 5. Are you confident that the outcome has been correctly measured for all patients (e.g. no outcomes are missed)? |
| Sample size and participant flow | 1. Were there a reasonable number of outcome events? |
|  | 2. Was the time interval between predictor assessment and outcome determination appropriate? |
|  | 3. Were all enrolled participants included in the analysis? |
|  | 4. Were participants with missing data handled appropriately? |
| Analysis | 1. Were any complexities in the data (e.g. censoring, competing risks) accounted for appropriately? |
|  | 2. Was the model *not* recalibrated before validation? |

This is a preliminary version of the PROBAST tool for risk of bias assessment of prediction model studies^1,2^.

**References**

1. Wolff RF, Moons KGM, Riley RD, Whiting PF, Westwood M, Collins GS, Reitsma JB, Kleijnen J, Mallett S: PROBAST: A Tool to Assess the Risk of Bias and Applicability of Prediction Model Studies. Ann Intern Med 2019, 170(1):51-58.
2. Moons KGM, Wolff RF, Riley RD, Whiting PF, Westwood M, Collins GS, Reitsma JB, Kleijnen J, Mallett S: PROBAST: A Tool to Assess Risk of Bias and Applicability of Prediction Model Studies: Explanation and Elaboration. Ann Intern Med 2019, 170(1):W1-w33.
