# Supplementary material for: Performance of the Framingham risk models and pooled cohort equations for predicting 10-year risk of cardiovascular disease: a systematic review and meta-analysis
Source: BMC Med. 2019 Jun 13;17:109. doi: 10.1186/s12916-019-1340-7 (PMC6563379; doi:10.1186/s12916-019-1340-7)
Supplement: Supplementary file 6 — Formulas used to estimate missing quantitative information. Overview of formulas used to estimate missing information on performance measures. (DOCX 23 kb) [file 12916_2019_1340_MOESM6_ESM.docx]

Additional file 6. Formulas used to estimate missing quantitative information

**Casemix variables**

For the casemix variables age, systolic blood pressure (SBP), HDL cholesterol and total cholesterol, we needed the mean and standard deviation (sd) for our analyses, however some studies only reported the median and 25^th^ and 75^th^ percentiles, or the minimum and maximum. If the median and percentiles were reported, we used equation 14 from a paper by Wan et al. to approximate the mean, and equation 16 to approximate the sd [1]. If only the range was reported, we used equation 5 from the same paper to approximate the sd. One study reported the number of participants in SBP, HDL cholesterol and total cholesterol categories [2]. To estimate the mean and sd, we took bootstrap samples from a uniform distribution per category, with sample size equal to the number of participants in the original categories, and calculated the mean and sd of this sample. This process was repeated 1000 times, and subsequently the overall (average) mean and sd were calculated.

**C-statistics**

If the precision of the c-statistic was not reported, we estimated this from the c-statistic and sample size of the study, using the formula described by Newcombe and Hanley [3, 4].

**OE ratio**

Various equations were used to estimate the standard error of the OE ratio, depending on which information was reported. All equations (as numbered) are described in the appendix of Debray et al [5]. If the SE of the OE ratio was reported, we used equation 16 to estimate the SE of ln(OE), if the observed event risk (Po), the expected event risk (Pe), and the SE of Po were reported, we used equation 51, and if only Po and Pe were reported we used equation 27.

If the OE ratio was reported for a prediction horizon shorter than 10 years, we extrapolated Po and Pe separately to 10 years using the following equation based on the Poisson distribution:

$$S_{KM,10}=\exp\left( \frac{{10 ln(S}_{KM,l})}{l} \right)$$

where $S_{KM,10}$ is the Kaplan Meier estimate of survival at 10 years, and $S_{KM,l}$ the Kaplan Meier survival estimate at time $l$. Po can be calculated by taking 1 - $S_{KM,10}$.

**References**

1. Wan X, Wang W, Liu J, Tong T. Estimating the sample mean and standard deviation from the sample size, median, range and/or interquartile range. BMC Med Res Methodol. 2014;14:135. Epub 2014/12/20. doi: 10.1186/1471-2288-14-135. PubMed PMID: 25524443; PubMed Central PMCID: PMCPmc4383202.

2. Mainous AG, 3rd, Koopman RJ, Diaz VA, Everett CJ, Wilson PWF, Tilley BC. A coronary heart disease risk score based on patient-reported information. Am J Cardiol. 2007;99(9):1236-41. PubMed PMID: 17478150; PubMed Central PMCID: PMCNIHMS22503, PMC1931421.

3. Newcombe RG. Confidence intervals for an effect size measure based on the Mann-Whitney statistic. Part 2: asymptotic methods and evaluation. Stat Med. 2006;25(4):559-73. Epub 2005/10/12. doi: 10.1002/sim.2324. PubMed PMID: 16217835.

4. Hanley JA, McNeil BJ. The meaning and use of the area under a receiver operating characteristic (ROC) curve. Radiology. 1982;143(1):29-36. Epub 1982/04/01. doi: 10.1148/radiology.143.1.7063747. PubMed PMID: 7063747.

5. Debray TP, Damen JA, Snell KI, Ensor J, Hooft L, Reitsma JB, et al. A guide to systematic review and meta-analysis of prediction model performance. BMJ. 2017;356:i6460. Epub 2017/01/07. doi: 10.1136/bmj.i6460. PubMed PMID: 28057641.
