# Supplementary material for: Performance of the Framingham risk models and pooled cohort equations for predicting 10-year risk of cardiovascular disease: a systematic review and meta-analysis
Source: BMC Med. 2019 Jun 13;17:109. doi: 10.1186/s12916-019-1340-7 (PMC6563379; doi:10.1186/s12916-019-1340-7)
Supplement: Supplementary file 7 — Statistical analyses. Details on the statistical analyses. (DOCX 24 kb) [file 12916_2019_1340_MOESM7_ESM.docx]

Additional file 7. Statistical analyses

Meta-analysis

The logit c-statistic and log OE ratio were pooled using random-effects meta-analyses accounting for the presence of between-study heterogeneity, weighted by the inverse of the variance. The Hartung-Knapp-Sidik-Jonkman (HKSJ) method was used when calculating 95% confidence intervals [1]. The 95% prediction interval was calculated using the equation described by Debray et al [2].

Calibration slope

The calibration slope can be calculated as follows:

$$O_{ij} \sim Binom\left( N_{ij},p_{ij} \right)$$

$$logit\left( P_{ij} \right)= \alpha_{i}+ \beta_{i}logit\left( P_{E,ij} \right)$$

$$\beta_{i} \sim N\left( \mu_{cal.slope},\tau_{cal.slope}^{2} \right)$$

Where $O_{ij}$ is the number of observed events in subgroup $j$ of study $i$, modeled using a binomial distribution with event probability $p_{ij}$. The calibration slope is given by $\hat{\mu}_{cal.slope}$.

Meta-regression

To investigate if the performance of the six models was influenced by differences in, for example, study populations, we fitted meta-regression models with a single covariate. The following categorical covariates were considered:

- age range of included participants: comparable (if both the upper and lower limit were within 5 years of the age range in the development population), narrower (if the lower limit was more than 5 years higher and/or the upper limit was more than 5 years lower), younger (if the lower limit was more than 5 years lower), older (if the upper limit was more than 5 years higher) or not reported (NR),
- in- or exclusion of participants with diabetes at baseline,
- in- or exclusion of participants with CHD or CVD at baseline,
- continent,
- prediction horizon: <10 year, 10 year, >10 year or NR,
- type of model used: for Wilson LDL or total cholesterol, for PCE white and others, or African American.

The following continuous covariates were included: mean and standard deviation of age, systolic blood pressure, HDL and total cholesterol, year in which the recruitment of participants for the study started, and the prediction horizon.

Sensitivity analyses

We performed several sensitivity analyses. Firstly, we excluded all external validations with high risk of bias for at least one domain. Secondly, since almost all validations scored high risk of bias for either the domain sample size and participant flow or analysis, we performed a second analysis in which we only excluded external validations with high risk of bias for any of the three domains: participant selection, predictors, or outcome. Thirdly, we used the number of events rather than the inverse of the variance as weighting factor in the meta-analysis, as suggested by Pennells et al. to increase statistical power [3]. Fourthly, we fitted a bivariate model with both the c-statistic and the 10-year total OE ratio as outcomes [4]. Fifthly, we repeated the analyses with the original OE ratio without extrapolating it to 10 years.

**References**

1. IntHout J, Ioannidis JP, Borm GF. The Hartung-Knapp-Sidik-Jonkman method for random effects meta-analysis is straightforward and considerably outperforms the standard DerSimonian-Laird method. BMC Med Res Methodol. 2014;14:25. Epub 2014/02/20. doi: 10.1186/1471-2288-14-25. PubMed PMID: 24548571; PubMed Central PMCID: PMCPmc4015721.

2. Debray TP, Damen JA, Snell KI, Ensor J, Hooft L, Reitsma JB, et al. A guide to systematic review and meta-analysis of prediction model performance. BMJ. 2017;356:i6460. Epub 2017/01/07. doi: 10.1136/bmj.i6460. PubMed PMID: 28057641.

3. Pennells L, Kaptoge S, White IR, Thompson SG, Wood AM. Assessing risk prediction models using individual participant data from multiple studies. Am J Epidemiol. 2014;179(5):621-32. Epub 2013/12/25. doi: 10.1093/aje/kwt298. PubMed PMID: 24366051; PubMed Central PMCID: PMCPmc3927974.

4. Snell KI, Hua H, Debray TP, Ensor J, Look MP, Moons KG, et al. Multivariate meta-analysis of individual participant data helped externally validate the performance and implementation of a prediction model. J Clin Epidemiol. 2015. Epub 2015/07/05. doi: 10.1016/j.jclinepi.2015.05.009. PubMed PMID: 26142114.
