# Supplementary material for: Performance of the Framingham risk models and pooled cohort equations for predicting 10-year risk of cardiovascular disease: a systematic review and meta-analysis
Source: BMC Med. 2019 Jun 13;17:109. doi: 10.1186/s12916-019-1340-7 (PMC6563379; doi:10.1186/s12916-019-1340-7)
Supplement: Supplementary file 8 — Description of outcomes. Overview showing which outcome definitions have been excluded and which definitions have been included. (DOCX 49 kb) [file 12916_2019_1340_MOESM8_ESM.docx]

Additional file 8. Description of outcomes

The table below gives an overview of the validations that were excluded because the outcome definition differed too much from the definition used in model development.

| **Model** | **Reference** | **Outcome category** | **Outcome definition** |
| --- | --- | --- | --- |
| Wilson men | Lee 2008 [1] | Fatal CVD | All deaths due to ischaemic heart disease (ICD-9 410-414) and cerebrovascular accidents (ICD-9 430-438). |
|  | Stork 2006 [2] | Fatal CVD | Not reported |
|  | Barroso 2010 [3] | Fatal or nonfatal CVD | Angina and myocardial infarction (fatal and non-fatal), and fatal cardiovascular disease (cardiac death of coronary and non-coronary origin, death of cerebrovascular origin, and deaths from other cardiovascular causes). |
| Wilson women | Lee 2008 [1] | Fatal CVD | All deaths due to ischaemic heart disease (ICD-9 410-414) and cerebrovascular accidents (ICD-9 430-438). |
|  | Barroso 2010 [3] | Fatal or nonfatal CVD | Angina and myocardial infarction (fatal and non-fatal), and fatal cardiovascular disease (cardiac death of coronary and non-coronary origin, death of cerebrovascular origin, and deaths from other cardiovascular causes). |
|  | Ridker 2007 [4] | Fatal or nonfatal CVD | Myocardial infarction, ischemic stroke, coronary revascularization, and cardiovascular deaths |
|  | Ridker 2007 [4] | Fatal or nonfatal CVD | Myocardial infarction, ischemic stroke, coronary revascularization, and cardiovascular deaths |
| ATP III men | Berry 2007 [5] | Fatal CHD | Coronary heart disease mortality |
|  | Berry 2007 [5] | Fatal CHD | Coronary heart disease mortality |
|  | Berry 2007 [5] | Fatal CHD | Coronary heart disease mortality |
|  | Berry 2007 [5] | Fatal CHD | Coronary heart disease mortality |
|  | Dunder 2004 [6] | Fatal or nonfatal MI | Hospitalization or death due to myocardial infarction (ICD 410/I 21). |
| ATP III women | Ridker 2007 [4] | Fatal or nonfatal CVD | Myocardial infarction, ischemic stroke, coronary revascularization, and cardiovascular deaths |

CVD: Cardiovascular disease, ICD: International Classification of Diseases, CHD: coronary heart disease, ATP: Adult treatment panel, MI: myocardial infarction

The following table gives an overview of the outcome definitions used in the validations that were included in the review (N=112), and their frequencies.

| **Model** | **Outcome definition** | **Frequency** |
| --- | --- | --- |
| Wilson men | "For this study, total events included all CAD events as defined in the Framingham Heart Study, using the same classifications and definitions." | 1 |
|  | angina and myocardial infarction, fatal and nonfatal | 1 |
|  | Angina pectoris, coronary insufficiency, MI, death due to CHD. | 1 |
|  | Angina pectoris, myocardial infarction or coronary insufficiency diagnosed and treated in a hospital | 1 |
|  | angina pectoris, recognized and unrecognized myocardial infarction, coronary insufficiency and CHD death including fatal myocardial infarction and sudden cardiac death | 1 |
|  | angina, fatal/non-fatal MI | 1 |
|  | CHD defined by ICD codes below. "These codes encompass the clinical spectrum of CHD (eg, unstable angina, stable angina, and myocardial infarction)." | 1 |
|  | CHD: myocardial infarction, fatal CHD, or cardiac procedure | 1 |
|  | Coronary death (MI, sudden cardiac death), nonfatal MI, coronary revascularization, silent MI | 1 |
|  | Coronary death, myocardial infarction and angina pectoris. Definite coronary death was defined as death with a documented coronary event. Sudden death was defined as death occurring within one hour following symptoms without explanation. | 2 |
|  | Fatal or non-fatal AMI, angina pectoris, unrecognised AMI | 1 |
|  | Hard CHD: acute myocardial infarction, sudden death and other coronary deaths | 1 |
|  | Hard CHD: coronary death or myocardial infarction | 7 |
|  | myocardial infarction or any coronary artery procedure leading to intervention (e.g., coronary artery bypass, stenting, or angioplasty) | 1 |
|  | Myocardial infarction, death from CHD, and angina | 1 |
|  | nonfatal myocardial infarction or coronary death (corresponding to hard events, as defined in the current FRS [5]), and hospitalization for angina or revascularization (coronary angioplasty or surgery) | 1 |
| Wilson women | "For this study, total events included all CAD events as defined in the Framingham Heart Study, using the same classifications and definitions." | 1 |
|  | angina and myocardial infarction, fatal and nonfatal | 1 |
|  | Angina pectoris, coronary insufficiency, MI, death due to CHD. | 1 |
|  | angina, fatal/non-fatal MI | 1 |
|  | CHD defined by ICD codes below. "These codes encompass the clinical spectrum of CHD (eg, unstable angina, stable angina, and myocardial infarction)." | 1 |
|  | CHD: myocardial infarction, fatal CHD, or cardiac procedure | 1 |
|  | Fatal or non-fatal AMI, angina pectoris, unrecognised AMI | 1 |
|  | Hard CHD: acute myocardial infarction, sudden death and other coronary deaths | 1 |
|  | Hard CHD: coronary death or myocardial infarction | 4 |
|  | myocardial infarction or any coronary artery procedure leading to intervention (e.g., coronary artery bypass, stenting, or angioplasty) | 1 |
|  | Myocardial infarction, death from CHD, and angina | 1 |
|  | nonfatal myocardial infarction or coronary death (corresponding to hard events, as defined in the current FRS [5]), and hospitalization for angina or revascularization (coronary angioplasty or surgery) | 1 |
| ATP III men | Fatal and nonfatal MI, atherosclerotic CHD death. | 2 |
|  | hard CHD: fatal and nonfatal MI and CHD mortality | 1 |
|  | Myocardial infarction and death from CHD | 1 |
|  | myocardial infarction or coronary death. | 1 |
|  | nonfatal MI and fatal CHD. The latter consisted of fatal MI (I21, I24), sudden cardiac death (I46, I49, R96), death from chronic ischemic heart disease (I25), and death due to heart failure (I50) other than hypertensive (I11) or nonrheumatic valve disorder | 1 |
|  | sudden coronary death, fatal acute myocardial infarction, and nonfatal acute myocardial infarction, coronary artery revascularization | 1 |
| ATP III women | Fatal and nonfatal MI, atherosclerotic CHD death. | 2 |
|  | hard CHD: fatal and nonfatal MI and CHD mortality | 1 |
|  | Myocardial infarction and death from CHD | 1 |
|  | myocardial infarction or coronary death. | 1 |
|  | nonfatal MI and fatal CHD. The latter consisted of fatal MI (I21, I24), sudden cardiac death (I46, I49, R96), death from chronic ischemic heart disease (I25), and death due to heart failure (I50) other than hypertensive (I11) or nonrheumatic valve disorder | 1 |
| PCE men | ASCVD event is defined as nonfatal myocardial infarction (MI), CHD death, nonfatal and fatal stroke. | 1 |
|  | death from CHD or fatal stroke or the first occurrence of nonfatal myocardial infarction or stroke | 2 |
|  | Fatal/non-fatal stroke, fatal/non-fatal MI | 1 |
|  | first ASCVD event, defined as nonfatal myocardial infarction or coronary heart disease death, or fatal or nonfatal stroke. | 2 |
|  | Hard ASCVD was defined as all-fatal or nonfatal myocardial infarction, stroke, and CHD or stroke-related mortality. | 1 |
|  | In the current study, first occurrence of CVD during the follow-up period was the targeted event. Cardiovascular diseases are defined as any coronary heart disease (CHD) (myocardial infarction, unstable angina, angiographic proven CHD and CHD death) and cerebrovascular events (ischemic or hemorrhagic stroke, and cerebrovascular death). Hard CVD event considered as first nonfatal MI, CHD death and fatal or nonfatal stroke | 1 |
|  | Incident myocardial infarction, nonfatal or fatal ischemic stroke (excluding transient ischemic attack), or death due to coronary artery disease | 1 |
|  | myocardial infarction, sudden cardiac death and fatal coronary heart disease, fatal/non-fatal stroke | 1 |
|  | nonfatal acute MI or CHD death or fatal or nonfatal stroke. Acute MI was identified as a change in biochemical markers of myocardial necrosis accompanied by ischemic symptoms, pathological Q waves, ST-segment elevation or depression, or coronary intervent | 6 |
|  | acute myocardial infarction, coronary heart disease (CHD) death, or fatal or nonfatal ischemic stroke | 1 |
|  | Angina pectoris, acute MI, CHD, chronic ischemic cerebral infarction, stroke, and transient ischemic attack | 1 |
|  | cardiovascular death, myocardial infarction, and stroke | 1 |
|  | Consistent with the definition used to derive the Pooled Cohort risk equations, the outcome for our primary analyses was defined as the first atherosclerotic cardiovascular disease event, which included a nonfatal or fatal stroke, a nonfatal MI, or CHD death. | 1 |
|  | first hard ASCVD event, including nonfatal myocardial infarction, coronary death, and stroke | 2 |
|  | hard ASCVD: fatal and nonfatal myocardial infarction (MI), other CHD mortality, and stroke | 1 |
|  | Hard CVD events included the first occurrence of nonfatal myocardial infarction, Coronary Heart Disease death, fatal or nonfatal stroke | 1 |
|  | myocardial infarction (MI), definite or probable angina, resuscitated cardiac arrest, coronary heart disease (CHD) death, stroke (not transient ischaemic attack), stroke death, other atherosclerotic death, or other CVD death. | 1 |
|  | Myocardial infarction, death from CHD, and stroke | 1 |
|  | Not reported | 4 |
| PCE women | acute myocardial infarction, coronary heart disease (CHD) death, or fatal or nonfatal ischemic stroke | 1 |
|  | Angina pectoris, acute MI, CHD, chronic ischemic cerebral infarction, stroke, and transient ischemic attack | 1 |
|  | ASCVD event is defined as nonfatal myocardial infarction (MI), CHD death, nonfatal and fatal stroke. | 1 |
|  | cardiovascular death, myocardial infarction, and stroke | 1 |
|  | Consistent with the definition used to derive the Pooled Cohort risk equations, the outcome for our primary analyses was defined as the first atherosclerotic cardiovascular disease event, which included a nonfatal or fatal stroke, a nonfatal MI, or CHD death. | 1 |
|  | death from CHD or fatal stroke or the first occurrence of nonfatal myocardial infarction or stroke | 2 |
|  | Fatal/non-fatal stroke, Data/non-fatal MI | 1 |
|  | first ASCVD event, defined as nonfatal myocardial infarction or coronary heart disease death, or fatal or nonfatal stroke. | 2 |
|  | first hard ASCVD event, including nonfatal myocardial infarction, coronary death, and stroke | 2 |
|  | Hard ASCVD was defined as all-fatal or nonfatal myocardial infarction, stroke, and CHD or stroke-related mortality. | 1 |
|  | hard ASCVD: fatal and nonfatal myocardial infarction (MI), other CHD mortality, and stroke | 1 |
|  | Hard CVD events included the first occurrence of nonfatal myocardial infarction, Coronary Heart Disease death, fatal or nonfatal stroke | 1 |
|  | In the current study, first occurrence of CVD during the follow-up period was the targeted event. Cardiovascular diseases are defined as any coronary heart disease (CHD) (myocardial infarction, unstable angina, angiographic proven CHD and CHD death) and cerebrovascular events (ischemic or hemorrhagic stroke, and cerebrovascular death). Hard CVD event considered as first nonfatal MI, CHD death and fatal or nonfatal stroke | 1 |
|  | Incident MI, stroke, and cardiovascular deaths. | 1 |
|  | Incident myocardial infarction, nonfatal or fatal ischemic stroke (excluding transient ischemic attack), or death due to coronary artery disease | 1 |
|  | myocardial infarction (MI), definite or probable angina, resuscitated cardiac arrest, coronary heart disease (CHD) death, stroke (not transient ischaemic attack), stroke death, other atherosclerotic death, or other CVD death. | 1 |
|  | Myocardial infarction, death from CHD, and stroke | 1 |
|  | myocardial infarction, sudden cardiac death and fatal coronary heart disease, fatal/non-fatal stroke | 1 |
|  | nonfatal acute MI or CHD death or fatal or nonfatal stroke. Acute MI was identified as a change in biochemical markers of myocardial necrosis accompanied by ischemic symptoms, pathological Q waves, ST-segment elevation or depression, or coronary intervent | 6 |
|  | Not reported | 4 |

**References**

1. Lee J, Heng D, Ma S, Chew S-K, Hughes K, Tai ES. The metabolic syndrome and mortality: the Singapore Cardiovascular Cohort Study. Clin Endocrinol (Oxf). 2008;69(2):225-30. doi: <http://dx.doi.org/10.1111/j.1365-2265.2008.03174.x>. PubMed PMID: 18208579.

2. Stork S, Feelders RA, van den Beld AW, Steyerberg EW, Savelkoul HFJ, Lamberts SWJ, et al. Prediction of mortality risk in the elderly. Am J Med. 2006;119(6):519-25. PubMed PMID: 16750966.

3. Barroso LC, Muro EC, Herrera ND, Ochoa GF, Hueros JIC, Buitrago F. Performance of the Framingham and SCORE cardiovascular risk prediction functions in a non-diabetic population of a Spanish health care centre: a validation study. Scand J Prim Health Care. 2010;28(4):242-8. doi: <http://dx.doi.org/10.3109/02813432.2010.518407>. PubMed PMID: 20873973; PubMed Central PMCID: PMCPMC3444797.

4. Ridker PM, Buring JE, Rifai N, Cook NR. Development and validation of improved algorithms for the assessment of global cardiovascular risk in women: the Reynolds Risk Score. JAMA. 2007;297(6):611-9. PubMed PMID: 17299196.

5. Berry JD, Lloyd-Jones DM, Garside DB, Greenland P. Framingham risk score and prediction of coronary heart disease death in young men. Am Heart J. 2007;154(1):80-6. PubMed PMID: 17584558; PubMed Central PMCID: PMCNIHMS26256 PMC2279177.

6. Dunder K, Lind L, Zethelius B, Berglund L, Lithell H. Evaluation of a scoring scheme, including proinsulin and the apolipoprotein B/apolipoprotein A1 ratio, for the risk of acute coronary events in middle-aged men: Uppsala Longitudinal Study of Adult Men (ULSAM). Am Heart J. 2004;148(4):596-601. PubMed PMID: 15459588.
