# Supplementary material for: Performance of the Framingham risk models and pooled cohort equations for predicting 10-year risk of cardiovascular disease: a systematic review and meta-analysis
Source: BMC Med. 2019 Jun 13;17:109. doi: 10.1186/s12916-019-1340-7 (PMC6563379; doi:10.1186/s12916-019-1340-7)
Supplement: Supplementary file 9 — Cohorts used multiple times to validate the same model. Overview of cohorts that were used more than once to validate the same model and which validation was subsequently chosen for which reason. (DOCX 310 kb) [file 12916_2019_1340_MOESM9_ESM.docx]

Additional file 9. Cohorts used multiple times to validate the same model

Below an overview is given of the cohorts that were used more than once to validate the same model, with rationale for the choice of cohort that was kept in the analyses, separately for validations included in the meta-analyses of calibration and discrimination.

OE ratio:

| **Reference** | **Cohort** | **Model** | **Excluded** | **Explanation** |
| --- | --- | --- | --- | --- |
| Jung 2015 [1] | Korean Heart Study | PCE men African American | Excluded | AHA guidelines advice to use the white model for this group of people |
| Jung 2015 [1] |  | PCE men white | Included |  |
| Jung 2015 [1] | Korean Heart Study | PCE women African American | Excluded | AHA guidelines advice to use the white model for this group of people |
| Jung 2015 [1] |  | PCE women white | Included |  |
| De Filippis 2015 [2] | MESA study | PCE men | Excluded | Most general population, fits review question best, most up-to-date population |
| De Filippis 2017 [3] |  | PCE men | Included |  |
| Goff 2014 [4] |  | PCE men African American | Excluded |  |
| Goff 2014 [4] |  | PCE men white | Excluded |  |
| De Filippis 2015 [2] | MESA study | PCE women | Excluded | Most general population, fits review question best, most up-to-date population |
| De Filippis 2017 [3] |  | PCE women | Included |  |
| Goff 2014 [4] |  | PCE women African American | Excluded |  |
| Goff 2014 [4] |  | PCE women white | Excluded |  |
| Muntner 2014 [5] | REGARDS study | PCE men | Included | Most general population, fits review question best |
| Goff 2014 [4] |  | PCE men African American | Excluded |  |
| Goff 2014 [4] |  | PCE men white | Excluded |  |
| Muntner 2014 [5] | REGARDS study | PCE women | Included | Most general population, fits review question best |
| Goff 2014 [4] |  | PCE women African American | Excluded |  |
| Goff 2014 [4] |  | PCE women white | Excluded |  |
| Yang 2016 [6] | China MUCA (1992) | PCE men African American | Excluded | AHA guidelines advice to use the white model for this group of people |
| Yang 2016 [6] |  | PCE men white | Included |  |
| Yang 2016 [6] | China MUCA (1992) | PCE women African American | Excluded | AHA guidelines advice to use the white model for this group of people |
| Yang 2016 [6] |  | PCE women white | Included |  |
| Yang 2016 [6] | CIMIC | PCE men African American | Excluded | AHA guidelines advice to use the white model for this group of people |
| Yang 2016 [6] |  | PCE men white | Included |  |
| Yang 2016 [6] | CIMIC | PCE women African American | Excluded | AHA guidelines advice to use the white model for this group of people |
| Yang 2016 [6] |  | PCE women white | Included |  |
| Yang 2016 [6] | InterASIA and China MUCA (1998) | PCE men African American | Excluded | AHA guidelines advice to use the white model for this group of people |
| Yang 2016 [6] |  | PCE men white | Included |  |
| Yang 2016 [6] | InterASIA and China MUCA (1998) | PCE women African American | Excluded | AHA guidelines advice to use the white model for this group of people |
| Yang 2016 [6] |  | PCE women white | Included |  |
| Mortensen 2015 [7] | Copenhagen General Population Study | PCE men | Excluded | Most recent data |
| Mortensen 2017 [8] |  | PCE men | Included |  |
| Mortensen 2015 [7] | Copenhagen General Population Study | PCE women | Excluded | Most recent data |
| Mortensen 2017 [8] |  | PCE women | Included |  |

C-statistic:

| **Reference** | **Cohort** | **Model** | **Excluded** | **Explanation for decision** |
| --- | --- | --- | --- | --- |
| Mainous 2007 [9] | ARIC study | Wilson men Total cholesterol | Included | Most general population, fits review question best |
| D'Agostino 2001 [10] |  | Wilson men Total cholesterol | Excluded |  |
| D'Agostino 2001 [10] |  | Wilson men Total cholesterol | Excluded |  |
| Mainous 2007 [9] | ARIC study | Wilson women Total cholesterol | Included | Most general population, fits review question best |
| D'Agostino 2001 [10] |  | Wilson women Total cholesterol | Excluded |  |
| D'Agostino 2001 [10] |  | Wilson women Total cholesterol | Excluded |  |
| Jung 2015 [1] | Korean Heart Study | PCE men African American | Excluded | AHA guidelines advice to use the white model for this group of people |
| Jung 2015 [1] |  | PCE men white | Included |  |
| Jung 2015 [1] | Korean Heart Study | PCE women African American | Excluded | AHA guidelines advice to use the white model for this group of people |
| Jung 2015 [1] |  | PCE women white | Included |  |
| DeFilippis 2015 [2] | MESA study | PCE men | Excluded | Most general population, fits review question best, most up-to-date population |
| De Filippis 2017 [3] |  | PCE men | Included |  |
| Goff 2014 [4] |  | PCE men African American | Excluded |  |
| Goff 2014 [4] |  | PCE men white | Excluded |  |
| DeFilippis 2015 [2] | MESA study | PCE women | Excluded | Most general population, fits review question best, most up-to-date population |
| De Filippis 2017 [3] |  | PCE women | Included |  |
| Goff 2014 [4] |  | PCE women African American | Excluded |  |
| Goff 2014 [4] |  | PCE women white | Excluded |  |
| Muntner 2014 [5] | REGARDS study | PCE men | Included | Most general population, fits review question best |
| Goff 2014 [4] |  | PCE men African American | Excluded |  |
| Goff 2014 [4] |  | PCE men white | Excluded |  |
| Muntner 2014 [5] | REGARDS study | PCE women | Included | Most general population, fits review question best |
| Goff 2014 [4] |  | PCE women African American | Excluded |  |
| Goff 2014 [4] |  | PCE women white | Excluded |  |
| Koller 2012 [11] | Rotterdam Study | ATP III men | Included | Most recent publication |
| Koller 2007 [12] |  | ATP III men | Excluded |  |
| Koller 2012 [11] | Rotterdam Study | ATP III women | Included | Most recent publication |
| Koller 2007 [12] |  | ATP III women | Excluded |  |
| Yang 2016 [6] | China MUCA (1992) | PCE men African American | Excluded | AHA guidelines advice to use the white model for this group of people |
| Yang 2016 [6] |  | PCE men white | Included |  |
| Yang 2016 [6] | China MUCA (1992) | PCE women African American | Excluded | AHA guidelines advice to use the white model for this group of people |
| Yang 2016 [6] |  | PCE women white | Included |  |
| Yang 2016 [6] | CIMIC | PCE men African American | Excluded | AHA guidelines advice to use the white model for this group of people |
| Yang 2016 [6] |  | PCE men white | Included |  |
| Yang 2016 [6] | CIMIC | PCE women African American | Excluded | AHA guidelines advice to use the white model for this group of people |
| Yang 2016 [6] |  | PCE women white | Included |  |
| Yang 2016 [6] | InterASIA and China MUCA (1998) | PCE men African American | Excluded | AHA guidelines advice to use the white model for this group of people |
| Yang 2016 [6] |  | PCE men white | Included |  |
| Yang 2016 [6] | InterASIA and China MUCA (1998) | PCE women African American | Excluded | AHA guidelines advice to use the white model for this group of people |
| Yang 2016 [6] |  | PCE women white | Included |  |
| Mortensen 2015 [7] | Copenhagen General Population Study | PCE men | Excluded | Most recent data |
| Mortensen 2017 [8] |  | PCE men | Included |  |
| Mortensen 2015 [7] | Copenhagen General Population Study | PCE women | Excluded | Most recent data |
| Mortensen 2017 [8] |  | PCE women | Included |  |

**References**

1. Jung KJ, Jang Y, Oh DJ, Oh BH, Lee SH, Park SW, et al. The ACC/AHA 2013 pooled cohort equations compared to a Korean Risk Prediction Model for atherosclerotic cardiovascular disease. Atherosclerosis. 2015;242(1):367-75. doi: <http://dx.doi.org/10.1016/j.atherosclerosis.2015.07.033>. PubMed PMID: 2015257443.

2. DeFilippis AP, Young R, Carrubba CJ, McEvoy JW, Budoff MJ, Blumenthal RS, et al. An analysis of calibration and discrimination among multiple cardiovascular risk scores in a modern multiethnic cohort. Ann Intern Med. 2015;162(4):266-75. doi: <http://dx.doi.org/10.7326/M14-1281>. PubMed PMID: 25686167.

3. De Filippis AP, Young R, McEvoy JW, Michos ED, Sandfort V, Kronmal RA, et al. Risk score overestimation: The impact of individual cardiovascular risk factors and preventive therapies on the performance of the American Heart Association-American College of Cardiology-Atherosclerotic Cardiovascular Disease risk score in a modern multi-ethnic cohort. Eur Heart J. 2017;38(8):598-608. doi: <http://dx.doi.org/10.1093/eurheartj/ehw301>. PubMed PMID: 615073852.

4. Goff DC, Jr., Lloyd-Jones DM, Bennett G, Coady S, D'Agostino RB, Gibbons R, et al. 2013 ACC/AHA guideline on the assessment of cardiovascular risk: a report of the American College of Cardiology/American Heart Association Task Force on Practice Guidelines. Circulation. 2014;129(25 Suppl 2):S49-73. Epub 2013/11/14. doi: 10.1161/01.cir.0000437741.48606.98. PubMed PMID: 24222018.

5. Muntner P, Colantonio LD, Cushman M, Goff DC, Jr., Howard G, Howard VJ, et al. Validation of the atherosclerotic cardiovascular disease Pooled Cohort risk equations. JAMA. 2014;311(14):1406-15. Epub 2014/04/01. doi: 10.1001/jama.2014.2630. PubMed PMID: 24682252; PubMed Central PMCID: PMCPmc4189930.

6. Yang X, Li J, Hu D, Chen J, Li Y, Huang J, et al. Predicting the 10-Year Risks of Atherosclerotic Cardiovascular Disease in Chinese Population: The China-PAR Project (Prediction for ASCVD Risk in China). Circulation. 2016;134(19):1430-40. doi: <http://dx.doi.org/10.1161/circulationaha.116.022367>. PubMed PMID: 27682885.

7. Mortensen MB, Afzal S, Nordestgaard BG, Falk E. Primary Prevention With Statins: ACC/AHA Risk-Based Approach Versus Trial-Based Approaches to Guide Statin Therapy. J Am Coll Cardiol. 2015;66(24):2699-709. doi: <http://dx.doi.org/10.1016/j.jacc.2015.09.089>. PubMed PMID: 26700832.

8. Mortensen MB, Nordestgaard BG, Afzal S, Falk E. ACC/AHA guidelines superior to ESC/EAS guidelines for primary prevention with statins in non-diabetic Europeans: the Copenhagen General Population Study. Eur Heart J. 2017;38(8):586-94. doi: <http://dx.doi.org/10.1093/eurheartj/ehw426>. PubMed PMID: 28363217.

9. Mainous AG, 3rd, Koopman RJ, Diaz VA, Everett CJ, Wilson PWF, Tilley BC. A coronary heart disease risk score based on patient-reported information. Am J Cardiol. 2007;99(9):1236-41. PubMed PMID: 17478150; PubMed Central PMCID: PMCNIHMS22503

PMC1931421.

10. D'Agostino RB, Sr., Grundy S, Sullivan LM, Wilson P. Validation of the Framingham coronary heart disease prediction scores: results of a multiple ethnic groups investigation. JAMA. 2001;286(2):180-7. Epub 2001/07/13. PubMed PMID: 11448281.

11. Koller MT, Leening MJG, Wolbers M, Steyerberg EW, Hunink MGM, Schoop R, et al. Development and validation of a coronary risk prediction model for older U.S. and European persons in the cardiovascular health study and the Rotterdam Study. Ann Intern Med. 2012;157(6):389-97. doi: <http://dx.doi.org/10.7326/0003-4819-157-6-201209180-00002>. PubMed PMID: 22986376.

12. Koller MT, Steyerberg EW, Wolbers M, Stijnen T, Bucher HC, Hunink MGM, et al. Validity of the Framingham point scores in the elderly: results from the Rotterdam study. Am Heart J. 2007;154(1):87-93. PubMed PMID: 17584559.
