# Supplementary material for: Performance of the Framingham risk models and pooled cohort equations for predicting 10-year risk of cardiovascular disease: a systematic review and meta-analysis
Source: BMC Med. 2019 Jun 13;17:109. doi: 10.1186/s12916-019-1340-7 (PMC6563379; doi:10.1186/s12916-019-1340-7)
Supplement: Supplementary file 10 — Characteristics of included validations. Table with an overview of the characteristics of included validations. (DOCX 315 kb) [file 12916_2019_1340_MOESM10_ESM.docx]

Additional file 10. Characteristics of included validations

| **Reference** | **Validated model** | **Recruitment years** | **Median FU time / Prediction horizon** | **Cohort** | **Country** | **Predicted outcome** | **N events / n participants (%)** | **Mean age (range)** | **C (SE)** | **OE (SE)** |
| --- | --- | --- | --- | --- | --- | --- | --- | --- | --- | --- |
| Andersson 2015 [1] | PCE men white | 1971-1998 | 10/10 | Framingham Heart Study Offspring Cohort | Framingham | Fatal or nonfatal CVD | 284/3396 (8.4%) | 53.3 (40-75) | 0.720 (0.014) | 0.840 (0.050) |
| Andersson 2015 [1] | PCE women white | 1971-1998 | 10/10 | Framingham Heart Study Offspring Cohort | Framingham | Fatal or nonfatal CVD | 112/3838 (2.9%) | 53.1 (40-75) | 0.773 (0.023) | 0.674 (0.070) |
| Buitrago 2011 [2] | Wilson men Total cholesterol | 1994-2004 | NR/10 | Patients ascribed to the La Paz healthcare centre in Badajoz, Spain | Spain | Fatal or nonfatal CHD | 22/201 (10.9%) | 50.9 (35-74) | 0.630 (0.061) | 0.673 (0.136) |
| Buitrago 2011 [2] | Wilson women Total cholesterol | 1994-2004 | NR/10 | Patients ascribed to the La Paz healthcare centre in Badajoz, Spain | Spain | Fatal or nonfatal CHD | 8/246 (3.3%) | 53.6 (35-74) | 0.650 (0.110) | 0.423 (0.146) |
| Chia 2014 [3] | PCE men white | 1998-1998 | NR/10 | Patients registered with an outpatient primary care clinic of University Malaya Medical Centre | Malaysia | Fatal or nonfatal CVD | 22/307 (7.2%) | 58.7 (40-79) | 0.550 (0.050) | 0.341 (0.070) |
| Chia 2014 [3] | PCE women white | 1998-1998 | NR/10 | Patients registered with an outpatient primary care clinic of University Malaya Medical Centre | Malaysia | Fatal or nonfatal CVD | 23/615 (3.7%) | 56.9 (40-79) | 0.610 (0.060) | 0.552 (0.114) |
| Comin 2007 [4] | Wilson men Total cholesterol | 1995-1998 | 5/5 | Patients from 67 health centers in autonomous Spanish regions | Spain | Fatal or nonfatal CHD | 137/3285 (5.6%) | 55.7 (35-74) | 0.679 (0.023) | 0.387 (0.038)** |
| Comin 2007 [4] | Wilson women Total cholesterol | 1995-1998 | 5/5 | Patients from 67 health centers in autonomous Spanish regions | Spain | Fatal or nonfatal CHD | 86/3285 (2.6%) | 56.8 (35-74) | 0.729 (0.030) | 0.359 (0.048)** |
| Cook 2014 [5] | PCE women | 1992-1995 | 10.2/10 | Womens Health Study | United States | Fatal or nonfatal CVD | 632/27542 (2.3%) | 54.2 (45-79) | NR | 0.611 (0.025) |
| Cooper 2005 [6] | ATP III men | 1989-NR | 10.8/10 | Second Northwick Park Heart Study | United Kingdom | Fatal or nonfatal CHD | 219/2732 (8%) | NR (50-64) | 0.620 (0.020) | 0.470 (NR) |
| D'Agostino 2001 [7]† | Wilson men Total cholesterol | 1987-1988 | NR/5 | ARIC study | United States | Fatal or nonfatal CHD | 149/4705 (3.2%) | 54.6 (44-66) | 0.750 (0.020) | 0.931 (0.074)* |
| D'Agostino 2001 [7]† | Wilson men Total cholesterol | 1987-1988 | NR/5 | ARIC study | United States | Fatal or nonfatal CHD | 46/1428 (3.2%) | 53.7 (44-66) | 0.670 (0.040) | 0.895 (0.127)* |
| D'Agostino 2001 [7] | Wilson men Total cholesterol | 1982-1982 | NR/5 | Physicians Health Study | United States | Fatal or nonfatal CHD | 182/901 (20.2%) | 57.6 (40-74) | 0.630 (0.023) | NR |
| D'Agostino 2001 [7] | Wilson men Total cholesterol | 1980-1982 | NR/5 | Honolulu Heart Program | United States | Fatal or nonfatal CHD | 77/2755 (2.8%) | 61.9 (51-81) | 0.720 (0.029) | 0.466 (0.051)* |
| D'Agostino 2001 [7] | Wilson men Total cholesterol | 1965-1968 | NR/5 | Puerto Rico Heart Health Program | Puerto Rico | Fatal or nonfatal CHD | 107/8713 (1.2%) | 54.1 (35-74) | 0.690 (0.026) | 0.352 (0.033)* |
| D'Agostino 2001 [7] | Wilson men Total cholesterol | 1989-1991 | NR/5 | Strong Heart Study | United States | Fatal or nonfatal CHD | 46/1527 (3%) | 55.4 (45-75) | 0.690 (0.039) | 0.698 (0.097)* |
| D'Agostino 2001 [7] | Wilson men Total cholesterol | 1989-1990 | NR/5 | Cardiovascular Health Study | United States | Fatal or nonfatal CHD | 71/956 (7.4%) | 69.7 (65-74) | 0.630 (0.034) | NR |
| D'Agostino 2001 [7]† | Wilson women Total cholesterol | 1987-1988 | NR/5 | ARIC study | United States | Fatal or nonfatal CHD | 52/5712 (0.9%) | 53.9 (44-66) | 0.830 (0.029) | 0.816 (0.11)* |
| D'Agostino 2001 [7]† | Wilson women Total cholesterol | 1987-1988 | NR/5 | ARIC study | United States | Fatal or nonfatal CHD | 38/2333 (1.6%) | 53.3 (44-66) | 0.790 (0.037) | 1.069 (0.163)* |
| D'Agostino 2001 [7] | Wilson women Total cholesterol | 1989-1991 | NR/5 | Strong Heart Study | United States | Fatal or nonfatal CHD | 23/2255 (1%) | 56.5 (45-75) | 0.750 (0.051) | 0.425 (0.082)* |
| D'Agostino 2001 [7] | Wilson women Total cholesterol | 1989-1990 | NR/5 | Cardiovascular Health Study | United States | Fatal or nonfatal CHD | 44/1601 (2.7%) | 69.3 (65-74) | 0.660 (0.041) | NR |
| De Filippis 2015 [8] | Wilson men Total cholesterol | 2000-2002 | NR/10 | MESA study | United States | Fatal or nonfatal CHD | 164/1961 (8.4%) | 61.5 (50-74) | 0.690 (0.020) | 0.640 (0.046) |
| De Filippis 2015 [8] | Wilson women Total cholesterol | 2000-2002 | NR/10 | MESA study | United States | Fatal or nonfatal CHD | 99/2266 (4.4%) | 61.5 (50-74) | 0.600 (0.028) | 0.680 (0.064) |
| De Filippis 2015 [8] | ATP III men | 2000-2002 | NR/10 | MESA study | United States | Fatal or nonfatal CHD | 86/1961 (4.4%) | 61.5 (50-74) | 0.710 (0.027) | 0.386 (0.04) |
| De Filippis 2015 [8] | ATP III women | 2000-2002 | NR/10 | MESA study | United States | Fatal or nonfatal CHD | 48/2266 (2.1%) | 61.5 (50-74) | 0.670 (0.039) | 0.693 (0.094) |
| De Filippis 2015 [8] | PCE men | 2000-2002 | NR/10 | MESA study | United States | Fatal or nonfatal CVD | 125/1961 (6.4%) | 61.5 (50-74) | 0.710 (0.021) | 0.531 (0.044) |
| De Filippis 2015 [8] | PCE women | 2000-2002 | NR/10 | MESA study | United States | Fatal or nonfatal CVD | 93/2266 (4.1%) | 61.5 (50-74) | 0.700 (0.027) | 0.599 (0.058) |
| De Filippis 2017 [9] | PCE men | 2000-2002 | NR/10 | MESA study | United States | Fatal or nonfatal CVD | 220/3053 (7.2%) | NR (45-79) | 0.710 (0.018) | 0.520 (0.034) |
| De Filippis 2017 [9] | PCE women | 2000-2002 | NR/10 | MESA study | United States | Fatal or nonfatal CVD | 149/3388 (4.4%) | NR (45-79) | 0.740 (0.021) | 0.500 (0.040) |
| De Las Heras Gala 2016 [10] | PCE men white | 1994-2001 | NR/10 | Kora study | Germany | Fatal or nonfatal CVD | 257/2584 (9.9%) | 56.4 (40-79) | 0.736 (0.015) | 0.700 (0.042) |
| De Las Heras Gala 2016 [10] | PCE women white | 1994-2001 | NR/10 | Kora study | Germany | Fatal or nonfatal CVD | 126/2654 (4.7%) | 55.5 (40-79) | 0.809 (0.019) | 0.800 (0.070) |
| De Las Heras Gala 2016 [10] | PCE men white | 2000-2003 | NR/10 | HNR study | Germany | Fatal or nonfatal CVD | 186/2005 (9.3%) | 58.8 (40-79) | 0.670 (0.020) | 0.620 (0.043) |
| De Las Heras Gala 2016 [10] | PCE women white | 2000-2003 | NR/10 | HNR study | Germany | Fatal or nonfatal CVD | 84/2203 (3.8%) | 59.1 (40-79) | 0.756 (0.026) | 0.590 (0.062) |
| Emdin 2017 [11] | PCE men | 2008-2009 | 2.7/10 | BioImage study | United States | Fatal or nonfatal CVD | 43/1635 (2.6%) | NR (55-80) | 0.630 (0.044) | 0.410 (0.062) |
| Emdin 2017 [11] | PCE women | 2008-2009 | 2.7/10 | BioImage study | United States | Fatal or nonfatal CVD | 31/2000 (1.6%) | NR (60-80) | 0.630 (0.031) | 0.330 (0.067) |
| Empana 2003 [12] | Wilson men LDL cholesterol | 1991-1993 | NR/5 | PRIME study | Northern Ireland | Fatal or nonfatal CHD | 120/2399 (5%) | NR (50-59) | 0.660 (0.025) | 0.761 (0.069)* |
| Empana 2003 [12] | Wilson men LDL cholesterol | 1991-1993 | NR/5 | PRIME study | France | Fatal or nonfatal CHD | 197/7359 (2.7%) | NR (50-59) | 0.680 (0.019) | 0.422 (0.030)* |
| Ferrario 2005 [13] | Wilson men Total cholesterol | 1983-1996 | 9.1/10 | CUORE study | Italy | Fatal or nonfatal CHD | 312/6865 (4.5%) | 50.8 (35-69) | 0.723 (0.028) | 0.374 (0.019) |
| Goff 2014 [14] | PCE men white | NR | NR/10 | ARIC study, Framingham Heart Study | Framingham | Fatal or nonfatal CVD | 539/5041 (10.7%) | NR (40-79) | 0.684 (0.012) | 0.727 (0.028) |
| Goff 2014 [14]†‡ | PCE men white | 2000-2002 | NR/6 | MESA study | United States | Fatal or nonfatal CVD | 57/1184 (4.8%) | NR (40-79) | 0.704 (0.035) | 0.636 (0.080)* |
| Goff 2014 [14]†‡ | PCE men white | 2003-2007 | NR/4 | REGARDS study | United States | Fatal or nonfatal CVD | 218/5296 (4.1%) | NR (40-79) | 0.595 (0.020) | 0.823 (0.051)* |
| Goff 2014 [14] | PCE men African American | NR | NR/10 | ARIC study, Framingham Heart Study | Framingham | Fatal or nonfatal CVD | 107/735 (14.6%) | NR (40-79) | 0.711 (0.027) | 0.944 (0.081) |
| Goff 2014 [14]†‡ | PCE men African American | 2000-2002 | NR/6 | MESA study | United States | Fatal or nonfatal CVD | 36/799 (4.5%) | NR (40-79) | 0.669 (0.046) | 0.538 (0.085)* |
| Goff 2014 [14]†‡ | PCE men African American | 2003-2007 | NR/4 | REGARDS study | United States | Fatal or nonfatal CVD | 136/2969 (4.6%) | NR (40-79) | 0.556 (0.025) | 0.904 (0.069)* |
| Goff 2014 [14] | PCE women white | NR | NR/10 | ARIC study, Framingham Heart Study | Framingham | Fatal or nonfatal CVD | 400/6509 (6.1%) | NR (40-79) | 0.738 (0.013) | 0.777 (0.036) |
| Goff 2014 [14]†‡ | PCE women white | 2000-2002 | NR/6 | MESA study | United States | Fatal or nonfatal CVD | 37/1273 (2.9%) | NR (40-79) | 0.711 (0.043) | 0.772 (0.123)* |
| Goff 2014 [14]†‡ | PCE women white | 2003-2007 | NR/4 | REGARDS study | United States | Fatal or nonfatal CVD | 101/6333 (1.6%) | NR (40-79) | 0.660 (0.027) | 0.787 (0.071)* |
| Goff 2014 [14] | PCE women African American | NR | NR/10 | ARIC study, Framingham Heart Study | Framingham | Fatal or nonfatal CVD | 127/1367 (9.3%) | NR (40-79) | 0.707 (0.024) | 0.944 (0.078) |
| Goff 2014 [14]†‡ | PCE women African American | 2000-2002 | NR/6 | MESA study | United States | Fatal or nonfatal CVD | 28/978 (2.8%) | NR (40-79) | 0.768 (0.045) | 0.512 (0.092)* |
| Goff 2014 [14]†‡ | PCE women African American | 2003-2007 | NR/4 | REGARDS study | United States | Fatal or nonfatal CVD | 126/5275 (2.4%) | NR (40-79) | 0.662 (0.024) | 0.683 (0.056)* |
| Jee 2014 [15] | Wilson men Total cholesterol | 1996-2001 | 11.6/10 | Korean Heart Study | South Korea | Fatal or nonfatal CHD | 2086/164005 (1.3%) | 45.8 (30-74) | NR | NR |
| Jee 2014 [15] | Wilson women Total cholesterol | 1996-2001 | 11.6/10 | Korean Heart Study | South Korea | Fatal or nonfatal CHD | 510/104310 (0.5%) | 47.6 (30-74) | NR | NR |
| Jung 2015 [16] | PCE men white | 1996-2001 | NR/10 | Korean Heart Study | South Korea | Fatal or nonfatal CVD | 7669/114622 (6.7%) | 50.1 (40-79) | 0.727 (0.003) | 0.634 (0.008) |
| Jung 2015 [16]†‡ | PCE men African American | 1996-2001 | NR/10 | Korean Heart Study | South Korea | Fatal or nonfatal CVD | 7669/114622 (6.7%) | 50.1 (40-79) | 0.725 (0.003) | 1.346 (0.023) |
| Jung 2015 [16] | PCE women white | 1996-2001 | NR/10 | Korean Heart Study | South Korea | Fatal or nonfatal CVD | 4658/77983 (6.0%) | 51.8 (40-79) | 0.738 (0.004) | 0.570 (0.007) |
| Jung 2015 [16]†‡ | PCE women African American | 1996-2001 | NR/10 | Korean Heart Study | South Korea | Fatal or nonfatal CVD | 4658/77983 (6.0%) | 51.8 (40-79) | 0.739 (0.004) | 0.754 (0.013) |
| Kavousi 2014 [17] | ATP III men | 1997-2001 | NR/10 | Rotterdam Study | Netherlands | Fatal or nonfatal CHD | 98/1431 (6.8%) | 64.9 (55-75) | 0.670 (0.026) | 0.422 (0.043) |
| Kavousi 2014 [17] | ATP III women | 1997-2001 | NR/10 | Rotterdam Study | Netherlands | Fatal or nonfatal CHD | 62/1976 (3.1%) | 65.1 (55-75) | 0.690 (0.031) | 0.574 (0.076) |
| Kavousi 2014 [17] | PCE men | 1997-2001 | NR/10 | Rotterdam Study | Netherlands | Fatal or nonfatal CVD | 192/1513 (12.7%) | 65.1 (55-75) | 0.670 (0.02) | 0.591 (0.04) |
| Kavousi 2014 [17] | PCE women | 1997-2001 | NR/10 | Rotterdam Study | Netherlands | Fatal or nonfatal CVD | 151/1920 (7.9%) | 65.2 (55-75) | 0.680 (0.023) | 0.681 (0.055) |
| Khalili 2015 [18] | PCE men white | 1999-2001 | 10.1/10 | Tehran Lipid and Glucose Study (TLGS) | Iran | Fatal or nonfatal CVD | 200/2353 (8.5%) | 54.6 (40-75) | 0.740 (0.018) | 0.758 (0.053) |
| Khalili 2015 [18] | PCE women white | 1999-2001 | 10.1/10 | Tehran Lipid and Glucose Study (TLGS) | Iran | Fatal or nonfatal CVD | 98/2749 (3.6%) | 52.5 (40-75) | 0.820 (0.021) | 0.839 (0.086) |
| Koller 2007 [19]† | ATP III men | 1990-1993 | 12.9/10 | Rotterdam Study | Netherlands | Fatal or nonfatal CHD | 351/2452 (14.3%) | 68.5 (55-NR) | 0.630 (0.057) | 0.722 (0.039) |
| Koller 2007 [19]† | ATP III women | 1990-1993 | 12.9/10 | Rotterdam Study | Netherlands | Fatal or nonfatal CHD | 448/4343 (10.3%) | 71.1 (55-NR) | 0.730 (0.049) | 0.980 (0.048) |
| Koller 2012 [20] | ATP III men | 1990-1993 | 14.9/10 | Rotterdam Study | Netherlands | Fatal or nonfatal CHD | 283/1454 (19.5%) | 73.3 (65-NR) | 0.600 (0.018) | NR |
| Koller 2012 [20] | ATP III men | 1989-1992 | 16.5/10 | Cardiovascular Health Study | United States | Fatal or nonfatal CHD | 563/1917 (29.4%) | 72.7 (65-NR) | 0.600 (0.015) | NR |
| Koller 2012 [20] | ATP III women | 1990-1993 | 14.9/10 | Rotterdam Study | Netherlands | Fatal or nonfatal CHD | 415/2849 (14.6%) | 76.3 (65-NR) | 0.650 (0.018) | NR |
| Koller 2012 [20] | ATP III women | 1989-1992 | 16.5/10 | Cardiovascular Health Study | United States | Fatal or nonfatal CHD | 603/3029 (19.9%) | 71.7 (65-NR) | 0.660 (0.013) | NR |
| Lee 2015 [21] | PCE men white | 1995-2004 | 10/10 | Hong Kong Cardiovascular Risk Factor Prevalence Study (CRISPS) cohort | ChiNR | Fatal or nonfatal CVD | 80/679 (11.8%) | 55.8 (40-74) | 0.714 (0.049) | 1.054 (0.102) |
| Lee 2015 [21] | PCE women white | 1995-2004 | 10/10 | Hong Kong Cardiovascular Risk Factor Prevalence Study (CRISPS) cohort | ChiNR | Fatal or nonfatal CVD | 42/797 (5.3%) | 53.4 (40-74) | 0.765 (0.039) | 1.438 (0.191) |
| Lloyd-Jones 2004 [22] | Wilson men Total cholesterol | 1971-NR | NR/10 | Framingham Heart Study | Framingham | Fatal or nonfatal CHD | NR/2716 (NR) | NR (40-94) | NR | NR |
| Lloyd-Jones 2004 [22] | Wilson women Total cholesterol | 1971-NR | NR/10 | Framingham Heart Study | Framingham | Fatal or nonfatal CHD | NR/3500 (NR) | NR (40-94) | NR | NR |
| Mainous 2007 [23] | Wilson men Total cholesterol | 1987-1989 | NR/10 | ARIC study | United States | Fatal or nonfatal CHD | NR/6239 (NR) | 54.4 (45-64) | 0.691 (0.011) | NR |
| Mainous 2007 [23] | Wilson women Total cholesterol | 1987-1989 | NR/10 | ARIC study | United States | Fatal or nonfatal CHD | NR/8104 (NR) | 53.8 (45-64) | 0.808 (0.008) | NR |
| Marrugat 2007 [24] | Wilson men Total cholesterol | 1995-1998 | NR/5 | VERIFICA study | Spain | Fatal or nonfatal CHD | 98/2447 (4%) | 55.7 (35-74) | 0.680 (0.024) | 0.407 (0.040)* |
| Marrugat 2007 [24] | Wilson women Total cholesterol | 1995-1998 | NR/5 | VERIFICA study | Spain | Fatal or nonfatal CHD | 56/3285 (1.7%) | 56.8 (35-74) | 0.730 (0.030) | 0.395 (0.053)* |
| Mortensen 2015 [25] | PCE men | 2003-2008 | NR/5 | Copenhagen General Population Study | Denmark | Fatal or nonfatal CVD | 467/16398 (2.8%) | 56 (40-75) | 0.647 (0.013) | 0.597 (0.027)* |
| Mortensen 2015 [25] | PCE women | 2003-2008 | NR/5 | Copenhagen General Population Study | Denmark | Fatal or nonfatal CVD | 367/21494 (1.7%) | 55.7 (40-75) | 0.669 (0.014) | 1.058 (0.055)* |
| Mortensen 2017 [26] | PCE men | 2003-2009 | NR/5 | Copenhagen General Population Study | Denmark | Fatal or nonfatal CVD | 1205/19383 (6.2%) | 56 (40-75) | 0.710 (0.008) | 0.661 (NR) |
| Mortensen 2017 [26] | PCE women | 2003-2009 | NR/5 | Copenhagen General Population Study | Denmark | Fatal or nonfatal CVD | 1012/25506 (4%) | 56 (40-75) | 0.710 (0.008) | 1.280 (NR) |
| Muntner 2014 [27] | PCE men | 2003-2007 | NR/5 | REGARDS study | United States | Fatal or nonfatal CVD | 376/NR (NR) | NR (45-79) | 0.650 (0.015) | 0.721 (0.035) |
| Muntner 2014 [27] | PCE women | 2003-2007 | NR/5 | REGARDS study | United States | Fatal or nonfatal CVD | 298/NR (NR) | NR (45-79) | 0.740 (0.013) | 0.813 (0.044) |
| Pike 2016 [28] | PCE men | 2005-2012 | NR/10 | Mayo Clinic Biobank | United States | Fatal or nonfatal CVD | 246/3093 (8%) | 59 (30-75) | 0.630 (0.018) | 0.610 (0.037) |
| Pike 2016 [28] | PCE women | 2005-2012 | NR/10 | Mayo Clinic Biobank | United States | Fatal or nonfatal CVD | 247/5690 (4.3%) | 56 (30-75) | 0.690 (0.015) | 0.610 (0.038) |
| Rana 2016 [29] | PCE men | 2008-2008 | NR/5 | Kaiser Permanente Northern California | United States | Fatal or nonfatal CVD | NR/118080 (NR) | NR (40-75) | 0.680 (NR) | NR |
| Rana 2016 [29] | PCE women | 2008-2008 | NR/5 | Kaiser Permanente Northern California | United States | Fatal or nonfatal CVD | NR/189511 (NR) | NR (40-75) | 0.720 (NR) | NR |
| Reissigova 2007 [30] | Wilson men Total cholesterol | 1975-1979 | NR/10 | Primary Prevention Study of Atherosclerotic Risk Factors (STULONG) | Czech Republic | Fatal or nonfatal CHD | 83/646 (12.8%) | 51.2 (38-49) | 0.638 (0.027) | 0.217 (0.019) |
| Rodondi 2012 [31] | Wilson men Total cholesterol | 1997-1998 | 8.3/7.5 | Health ABC Study | United States | Fatal or nonfatal CHD | 205/981 (20.9%) | 73.6 (70-79) | 0.583 (0.024) | 1.083 (0.068)* |
| Rodondi 2012 [31] | Wilson women Total cholesterol | 1997-1998 | 8.3/7.5 | Health ABC Study | United States | Fatal or nonfatal CHD | 146/1212 (12%) | 73.4 (70-79) | 0.577 (0.028) | 2.049 (0.167)* |
| Ryckman 2015 [32] | Wilson men Unclear | 2004-2005 | NR/NR | Series of adults undergoing colorectal cancer screening | United States | Fatal or nonfatal CHD | NR/NR (NR) | NR (NR) | NR | NR |
| Ryckman 2015 [32] | Wilson women Unclear | 2004-2005 | NR/NR | Series of adults undergoing colorectal cancer screening | United States | Fatal or nonfatal CHD | NR/NR (NR) | NR (NR) | NR | NR |
| Simmons 2008 [33] | Wilson men Total cholesterol | 1993-1998 | NR/10 | EPIC-Norfolk | United Kingdom | Fatal or nonfatal CHD | 430/4513 (9.5%) | 58.3 (40-79) | 0.710 (0.010) | 0.546 (0.025) |
| Simmons 2008 [33] | Wilson women Total cholesterol | 1993-1998 | NR/10 | EPIC-Norfolk | United Kingdom | Fatal or nonfatal CHD | 250/5782 (4.3%) | 57.6 (40-79) | 0.710 (0.015) | 0.560 (0.036) |
| Simons 2003 [34] | ATP III men | 1988-1989 | NR/10 | Dubbo Study | Australia | Fatal or nonfatal CHD | 105/755 (13.9%) | NR (60-79) | NR | 0.954 (0.086) |
| Simons 2003 [34] | ATP III women | 1988-1989 | NR/10 | Dubbo Study | Australia | Fatal or nonfatal CHD | 80/1045 (7.7%) | NR (60-79) | NR | 0.899 (0.096) |
| Suka 2001 [35] | Wilson men Total cholesterol | 1991-1993 | NR/NR | Employee health management center in a Japanese company | Japan | Fatal or nonfatal CHD | 80/5611 (1.4%) | 44.7 (30-59) | 0.710 (0.029) | NR |
| Sussman 2017 [36] | PCE men | 2007-NR | NR/5 | US Department of Veterans Affairs | United States | Fatal or nonfatal CVD | 80412/1435937 (5.6%) | 62 (45-80) | 0.657 (0.001) | 0.627 (0.002)* |
| Sussman 2017 [36] | PCE women | 2007-NR | NR/5 | US Department of Veterans Affairs | United States | Fatal or nonfatal CVD | 1599/76155 (2.1%) | 55.6 (45-80) | 0.726 (0.006) | 0.914 (0.023)* |
| Vaidya 2007 [37] | Wilson men Total cholesterol | 1983-1996 | NR/10 | 10 Baltimore area hospitals | United States | Fatal or nonfatal CHD | 81/404 (20%) | 45.2 (30-59) | 0.698 (0.03) | 1.701 (0.170) |
| Vaidya 2007 [37] | Wilson women Total cholesterol | 1983-1996 | NR/10 | 10 Baltimore area hospitals | United States | Fatal or nonfatal CHD | 27/380 (7.1%) | 46.1 (30-59) | 0.787 (0.04) | 1.141 (0.212) |
| Yang 2016 [38] | PCE men white | 1998-2001 | NR/10 | InterASIA and China MUCA (1998) | China | Fatal or nonfatal CVD | 451/10334 (4.4%) | 48.8 (35-74) | 0.762 (0.011) | 0.657 (0.030) |
| Yang 2016 [38] | PCE men white | 1992-1994 | NR/10 | China MUCA (1992) | China | Fatal or nonfatal CVD | 216/6565 (3.3%) | 46.5 (35-59) | 0.768 (0.018) | 0.649 (0.043) |
| Yang 2016 [38] | PCE men white | 2007-2008 | NR/5 | CIMIC | China | Fatal or nonfatal CVD | 755/26872 (2.8%) | 55.3 (16-99) | 0.761 (0.009) | 0.636 (0.023)* |
| Yang 2016 [38] | PCE women white | 1998-2001 | NR/10 | InterASIA and China MUCA (1998) | China | Fatal or nonfatal CVD | 285/10986 (2.6%) | 48.4 (35-74) | 0.783 (0.014) | 1.102 (0.064) |
| Yang 2016 [38] | PCE women white | 1992-1994 | NR/10 | China MUCA (1992) | China | Fatal or nonfatal CVD | 168/7558 (2.2%) | 46.6 (35-59) | 0.786 (0.017) | 1.368 (0.104) |
| Yang 2016 [38] | PCE women white | 2007-2008 | NR/5 | CIMIC | China | Fatal or nonfatal CVD | 738/43966 (1.7%) | 53.9 (16-99) | 0.785 (0.007) | 1.110 (0.041)* |
| Yang 2016 [38] | PCE men African American | 1998-2001 | NR/10 | InterASIA and China MUCA (1998) | China | Fatal or nonfatal CVD | 451/10334 (4.4%) | 48.8 (35-74) | 0.769 (0.011) | 0.562 (0.026) |
| Yang 2016 [38] | PCE men African American | 1992-1994 | NR/10 | China MUCA (1992) | China | Fatal or nonfatal CVD | 216/6565 (3.3%) | 46.5 (35-59) | 0.790 (0.017) | 0.482 (0.032) |
| Yang 2016 [38] | PCE men African American | 2007-2008 | NR/5 | CIMIC | China | Fatal or nonfatal CVD | 755/26872 (2.8%) | 55.3 (16-99) | 0.750 (0.008) | 0.600 (0.022)* |
| Yang 2016 [38] | PCE women African American | 1998-2001 | NR/10 | InterASIA and China MUCA (1998) | China | Fatal or nonfatal CVD | 285/10986 (2.6%) | 48.4 (35-74) | 0.796 (0.013) | 0.715 (0.042) |
| Yang 2016 [38] | PCE women African American | 1992-1994 | NR/10 | China MUCA (1992) | China | Fatal or nonfatal CVD | 168/7558 (2.2%) | 46.6 (35-59) | 0.807 (0.016) | 0.794 (0.061) |
| Yang 2016 [38] | PCE women African American | 2007-2008 | NR/5 | CIMIC | China | Fatal or nonfatal CVD | 738/43966 (1.7%) | 53.9 (16-99) | 0.792 (0.007) | 0.699 (0.026)* |

* OE ratio extrapolated to 10 years

** OE ratio and corresponding SE extrapolated to 10 years

† Not included in analyses of c-statistic because model was validated more than once in the same cohort

‡ Not included in analyses of OE ratio because model was validated more than once in the same cohort

FU: follow-up, N: number, C: c-statistic, OE: observed/expected ratio, SE: standard error, NR: Not reported, CHD: coronary heart disease, CVD: cardiovascular disease.

A summary of the reported case-mix in the included validation studies

|  | **Wilson men** | **Wilson women** | **ATPIII men** | **ATPIII women** | **PCE men** | **PCE women** |
| --- | --- | --- | --- | --- | --- | --- |
| Total N | 23 | 15 | 7 | 6 | 30 | 31 |
| Eligibility age - comparable | 6 (26.1%) | 4 (26.7%) | 0 (0.0%) | 0 (0.0%) | 22 (73.3%) | 23 (74.2%) |
| Eligibility age - younger | 0 (0.0%) | 0 (0.0%) | 0 (0.0%) | 0 (0.0%) | 1 (3.3%) | 1 (3.2%) |
| Eligibility age - older | 2 (8.7%) | 1 (6.7%) | 0 (0.0%) | 0 (0.0%) | 0 (0.0%) | 0 (0.0%) |
| Eligibility age - narrower | 14 (60.9%) | 9 (60.0%) | 4 (57.1%) | 3 (50.0%) | 5 (16.7%) | 5 (16.1%) |
| Eligibility age - broader | 0 (0.0%) | 0 (0.0%) | 0 (0.0%) | 0 (0.0%) | 2 (6.7%) | 2 (6.5%) |
| Eligibility age - NR | 1 (4.3%) | 1 (6.7%) | 3 (42.9%) | 3 (50.0%) | 0 (0.0%) | 0 (0.0%) |
| Eligibility CHD - not excluded | 6 (26.1%) | 4 (26.7%) | 0 (0.0%) | 0 (0.0%) | 1 (3.3%) | 1 (3.2%) |
| Eligibility CHD - CHD excl | 9 (39.1%) | 6 (40.0%) | 0 (0.0%) | 0 (0.0%) | 1 (3.3%) | 1 (3.2%) |
| Eligibility CHD - CVD excl | 8 (34.8%) | 5 (33.3%) | 7 (100.0%) | 6 (100.0%) | 28 (93.3%) | 29 (93.5%) |
| Eligibility diabetes - not excl | 20 (87.0%) | 13 (86.7%) | 4 (57.1%) | 3 (50.0%) | 26 (86.7%) | 27 (87.1%) |
| Eligibility diabetes - excl | 3 (13.0%) | 2 (13.3%) | 3 (42.9%) | 3 (50.0%) | 4 (13.3%) | 4 (12.9%) |
| Treated individuals - not excl | 21 (91.3%) | 14 (93.3%) | 5 (71.4%) | 5 (83.3%) | 20 (66.7%) | 22 (71.0%) |
| Treated individuals - excl | 2 (8.7%) | 1 (6.7%) | 2 (28.6%) | 1 (16.7%) | 10 (33.3%) | 9 (29.0%) |
| Age mean | 58.0 (54.6-73.6), NR=4 | 57.6 (56.5-73.4), NR=2 | 72.7 (68.5-73.3), NR=2 | 71.7 (71.1-76.3), NR=1 | 58.7 (55.6-65.1), NR=10 | 56.0 (53.9-65.2), NR=10 |
| Age sd | 9.6 (7.4-13.5), NR=1 | 9.9 (7.4-13.5), NR=1 | 6.5 (5.6-7.1), NR=1 | 7.1 (5.9-9.6), NR=1 | 9.8 (9.4-11.9), NR=0 | 9.8 (9.0-11.9), NR=0 |
| Smoking | 43.9 (38.0-59.8), NR=4 | 25.0 (13.4-34.7), NR=2 | 30.0 (20.1-30.1), NR=2 | 16.7 (13.0-19.3), NR=1 | 50.2 (26.9-70.1), NR=10 | 19.4 (6.3-26.7), NR=11 |
| Diabetes | 14.5 (7.0-42.0), NR=4 | 14.6 (7.4-51.0), NR=2 | 10.0 (0.0-17.0), NR=2 | 12.0 (0.0-13.0), NR=1 | 12.2 (6.2-43.0), NR=11 | 7.8 (5.5-43.6), NR=11 |
| SBP mean | 135.2 (132.4-138.5), NR=11 | 135.0 (133.1-135.8), NR=6 | 139.9 (136.6-142.0), NR=3 | 140.3 (136.8-144.3), NR=2 | 136.8 (127.9-143.0), NR=10 | 130.3 (126.3-140.0), NR=10 |
| SBP sd | 18.6 (17.4-21.0), NR=11 | 19.6 (18.8-22.0), NR=6 | 21.1 (21.0-21.3), NR=3 | 21.9 (21.8-22.0), NR=2 | 19.4 (18.1-21.0), NR=10 | 20.8 (20.6-22.4), NR=10 |
| HDL mean | 49.5 (47.9-53.5), NR=4 | 58.0 (56.3-62.0), NR=2 | 49.8 (47.7-52.0), NR=3 | 58.4 (57.5-59.8), NR=2 | 50.4 (50.0-54.1), NR=11 | 59.7 (54.2-69.6), NR=11 |
| HDL sd | 13.9 (12.0-15.5), NR=4 | 15.7 (12.7-19.0), NR=2 | 13.1 (12.0-15.0), NR=3 | 16.3 (15.6-17.2), NR=2 | 14.4 (13.4-17.2), NR=11 | 16.2 (15.0-20.1), NR=11 |
| Total cholesterol mean | 226.9 (212.6-239.3), NR=4 | 234.0 (216.7-239.0), NR=2 | 225.4 (209.7-234.2), NR=3 | 242.2 (227.1-258.7), NR=2 | 217.0 (196.9-235.3), NR=10 | 224.3 (203.0-239.8), NR=10 |
| Total cholesterol sd | 40.2 (37.2-43.7), NR=4 | 42.0 (38.0-52.7), NR=2 | 37.7 (35.5-43.0), NR=3 | 39.3 (36.5-45.8), NR=2 | 37.4 (36.1-42.5), NR=10 | 40.1 (38.2-47.4), NR=10 |

Values indicate N (%), or median (IQR)

PCE: Pooled Cohort Equations, NR: not reported, CHD: coronary heart disease, CVD: cardiovascular disease, excl: excluded, sd: standard deviation, SBP: systolic blood pressure, HDL: high density lipoprotein cholesterol

**References**

1. Andersson C, Enserro D, Larson MG, Xanthakis V, Vasan RS. Implications of the US cholesterol guidelines on eligibility for statin therapy in the community: comparison of observed and predicted risks in the Framingham Heart Study Offspring Cohort. J Am Heart Assoc. 2015;4(4). doi: 10.1161/JAHA.115.001888.

2. Buitrago F, Calvo-Hueros JI, Canon-Barroso L, Pozuelos-Estrada G, Molina-Martinez L, Espigares-Arroyo M, et al. Original and REGICOR Framingham functions in a nondiabetic population of a Spanish health care center: a validation study. Ann Fam Med. 2011;9(5):431-8. doi: 10.1370/afm.1287.

3. Chia YC, Lim HM, Ching SM. Validation of the pooled cohort risk score in an Asian population - a retrospective cohort study. BMC Cardiovasc Disord. 2014;14:163. doi: 10.1186/1471-2261-14-163.

4. Comin E, Solanas P, Cabezas C, Subirana I, Ramos R, Gene-Badia J, et al. Estimating cardiovascular risk in Spain using different algorithms. Rev Esp Cardiol. 2007;60(7):693-702. doi: 10.1157/13108274.

5. Cook NR, Ridker PM. Further insight into the cardiovascular risk calculator: the roles of statins, revascularizations, and underascertainment in the Women's Health Study. JAMA Intern Med. 2014;174(12):1964-71. doi: 10.1001/jamainternmed.2014.5336.

6. Cooper JA, Miller GJ, Humphries SE. A comparison of the PROCAM and Framingham point-scoring systems for estimation of individual risk of coronary heart disease in the Second Northwick Park Heart Study. Atherosclerosis. 2005;181(1):93-100.

7. D'Agostino RB, Sr., Grundy S, Sullivan LM, Wilson P. Validation of the Framingham coronary heart disease prediction scores: results of a multiple ethnic groups investigation. JAMA. 2001;286(2):180-7.

8. DeFilippis AP, Young R, Carrubba CJ, McEvoy JW, Budoff MJ, Blumenthal RS, et al. An analysis of calibration and discrimination among multiple cardiovascular risk scores in a modern multiethnic cohort. Ann Intern Med. 2015;162(4):266-75. doi: 10.7326/M14-1281.

9. DeFilippis AP, Young R, McEvoy JW, Michos ED, Sandfort V, Kronmal RA, et al. Risk score overestimation: The impact of individual cardiovascular risk factors and preventive therapies on the performance of the American Heart Association-American College of Cardiology-Atherosclerotic Cardiovascular Disease risk score in a modern multi-ethnic cohort. Eur Heart J. 2017;38(8):598-608. doi: 10.1093/eurheartj/ehw301.

10. De Las Heras Gala T, Geisel MH, Peters A, Thorand B, Baumert J, Lehmann N, et al. Recalibration of the ACC/AHA risk score in two population-based German cohorts. PLoS One. 2016;11 (10)(e0164688):e0164688. doi: 10.1371/journal.pone.0164688.

11. Emdin CA, Khera AV, Natarajan P, Klarin D, Baber U, Mehran R, et al. Evaluation of the Pooled Cohort Equations for Prediction of Cardiovascular Risk in a Contemporary Prospective Cohort. Am J Cardiol. 2017;119(6):881-5. doi: 10.1016/j.amjcard.2016.11.042.

12. Empana JP, Ducimetiere P, Arveiler D, Ferrieres J, Evans A, Ruidavets JB, et al. Are the Framingham and PROCAM coronary heart disease risk functions applicable to different European populations? The PRIME Study. Eur Heart J. 2003;24(21):1903-11.

13. Ferrario M, Chiodini P, Chambless LE, Cesana G, Vanuzzo D, Panico S, et al. Prediction of coronary events in a low incidence population. Assessing accuracy of the CUORE Cohort Study prediction equation. Int J Epidemiol. 2005;34(2):413-21.

14. Goff DC, Jr., Lloyd-Jones DM, Bennett G, Coady S, D'Agostino RB, Gibbons R, et al. 2013 ACC/AHA guideline on the assessment of cardiovascular risk: a report of the American College of Cardiology/American Heart Association Task Force on Practice Guidelines. Circulation. 2014;129(25 Suppl 2):S49-73. doi: 10.1161/01.cir.0000437741.48606.98.

15. Jee SH, Jang Y, Oh DJ, Oh BH, Lee SH, Park SW, et al. A coronary heart disease prediction model: The Korean heart study. BMJ Open. 2014;4(5). doi: 10.1136/bmjopen-2014-005025.

16. Jung KJ, Jang Y, Oh DJ, Oh BH, Lee SH, Park SW, et al. The ACC/AHA 2013 pooled cohort equations compared to a Korean Risk Prediction Model for atherosclerotic cardiovascular disease. Atherosclerosis. 2015;242(1):367-75. doi: 10.1016/j.atherosclerosis.2015.07.033.

17. Kavousi M, Leening MJ, Nanchen D, Greenland P, Graham IM, Steyerberg EW, et al. Comparison of application of the ACC/AHA guidelines, Adult Treatment Panel III guidelines, and European Society of Cardiology guidelines for cardiovascular disease prevention in a European cohort. JAMA. 2014;311(14):1416-23. doi: 10.1001/jama.2014.2632.

18. Khalili D, Asgari S, Hadaegh F, Steyerberg EW, Rahimi K, Fahimfar N, et al. A new approach to test validity and clinical usefulness of the 2013 ACC/AHA guideline on statin therapy: A population-based study. Int J Cardiol. 2015;184(1):587-94. doi: 10.1016/j.ijcard.2015.03.067.

19. Koller MT, Steyerberg EW, Wolbers M, Stijnen T, Bucher HC, Hunink MGM, et al. Validity of the Framingham point scores in the elderly: results from the Rotterdam study. Am Heart J. 2007;154(1):87-93.

20. Koller MT, Leening MJG, Wolbers M, Steyerberg EW, Hunink MGM, Schoop R, et al. Development and validation of a coronary risk prediction model for older U.S. and European persons in the cardiovascular health study and the Rotterdam Study. Ann Intern Med. 2012;157(6):389-97. doi: 10.7326/0003-4819-157-6-201209180-00002.

21. Lee CH, Woo YC, Lam JKY, Fong CHY, Cheung BMY, Lam KSL, et al. Validation of the Pooled Cohort equations in a long-term cohort study of Hong Kong Chinese. J Clin Lipidol. 2015;9(5):640-6. doi: 10.1016/j.jacl.2015.06.005.

22. Lloyd-Jones DM, Wilson PWF, Larson MG, Beiser A, Leip EP, D'Agostino RB, et al. Framingham risk score and prediction of lifetime risk for coronary heart disease. Am J Cardiol. 2004;94(1):20-4.

23. Mainous AG, 3rd, Koopman RJ, Diaz VA, Everett CJ, Wilson PWF, Tilley BC. A coronary heart disease risk score based on patient-reported information. Am J Cardiol. 2007;99(9):1236-41.

24. Marrugat J, Subirana I, Comin E, Cabezas C, Vila J, Elosua R, et al. Validity of an adaptation of the Framingham cardiovascular risk function: the VERIFICA Study. J Epidemiol Community Health. 2007;61(1):40-7. doi: 10.1136/jech.2005.038505.

25. Mortensen MB, Afzal S, Nordestgaard BG, Falk E. Primary Prevention With Statins: ACC/AHA Risk-Based Approach Versus Trial-Based Approaches to Guide Statin Therapy. J Am Coll Cardiol. 2015;66(24):2699-709. doi: 10.1016/j.jacc.2015.09.089.

26. Mortensen MB, Nordestgaard BG, Afzal S, Falk E. ACC/AHA guidelines superior to ESC/EAS guidelines for primary prevention with statins in non-diabetic Europeans: the Copenhagen General Population Study. Eur Heart J. 2017;38(8):586-94. doi: 10.1093/eurheartj/ehw426.

27. Muntner P, Colantonio LD, Cushman M, Goff DC, Jr., Howard G, Howard VJ, et al. Validation of the atherosclerotic cardiovascular disease Pooled Cohort risk equations. JAMA. 2014;311(14):1406-15. doi: 10.1001/jama.2014.2630.

28. Pike MM, Decker PA, Larson NB, St Sauver JL, Takahashi PY, Roger VL, et al. Improvement in Cardiovascular Risk Prediction with Electronic Health Records. J Cardiovasc Transl Res. 2016;9(3):214-22. doi: 10.1007/s12265-016-9687-z.

29. Rana JS, Tabada GH, Solomon MD, Lo JC, Jaffe MG, Sung SH, et al. Accuracy of the Atherosclerotic Cardiovascular Risk Equation in a Large Contemporary, Multiethnic Population. J Am Coll Cardiol. 2016;67(18):2118-30. doi: 10.1016/j.jacc.2016.02.055.

30. Reissigova J, Zvarova J. The Framingham risk function underestimated absolute coronary heart disease risk in Czech men. Methods Inf Med. 2007;46(1):43-9.

31. Rodondi N, Locatelli I, Aujesky D, Butler J, Vittinghoff E, Simonsick E, et al. Framingham risk score and alternatives for prediction of coronary heart disease in older adults. PLoS One. 2012;7(3):e34287. doi: 10.1371/journal.pone.0034287.

32. Ryckman EM, Summers RM, Liu J, Munoz del Rio A, Pickhardt PJ. Visceral fat quantification in asymptomatic adults using abdominal CT: is it predictive of future cardiac events? Abdom Imaging. 2015;40(1):222-6.

33. Simmons RK, Sharp S, Boekholdt SM, Sargeant LA, Khaw K-T, Wareham NJ, et al. Evaluation of the Framingham risk score in the European Prospective Investigation of Cancer-Norfolk cohort: does adding glycated hemoglobin improve the prediction of coronary heart disease events? Arch Intern Med. 2008;168(11):1209-16. doi: 10.1001/archinte.168.11.1209.

34. Simons LA, Simons J, Friedlander Y, McCallum J, Palaniappan L. Risk functions for prediction of cardiovascular disease in elderly Australians: the Dubbo Study. Med J Aust. 2003;178(3):113-6.

35. Suka M, Sugimori H, Yoshida K. Application of the updated Framingham risk score to Japanese men. Hypertens Res. 2001;24(6):685-9.

36. Sussman JB, Wiitala WL, Zawistowski M, Hofer TP, Bentley D, Hayward RA. The Veterans Affairs Cardiac Risk Score: Recalibrating the Atherosclerotic Cardiovascular Disease Score for Applied Use. Med Care. 2017;55(9):864-70. doi: 10.1097/mlr.0000000000000781.

37. Vaidya D, Yanek LR, Moy TF, Pearson TA, Becker LC, Becker DM. Incidence of coronary artery disease in siblings of patients with premature coronary artery disease: 10 years of follow-up. Am J Cardiol. 2007;100(9):1410-5.

38. Yang X, Li J, Hu D, Chen J, Li Y, Huang J, et al. Predicting the 10-Year Risks of Atherosclerotic Cardiovascular Disease in Chinese Population: The China-PAR Project (Prediction for ASCVD Risk in China). Circulation. 2016;134(19):1430-40. doi: 10.1161/circulationaha.116.022367.
