# Supplementary material for: Performance of the Framingham risk models and pooled cohort equations for predicting 10-year risk of cardiovascular disease: a systematic review and meta-analysis
Source: BMC Med. 2019 Jun 13;17:109. doi: 10.1186/s12916-019-1340-7 (PMC6563379; doi:10.1186/s12916-019-1340-7)
Supplement: Supplementary file 11 — Summary calibration slope. Table with results from the pooled calibration slope. (DOCX 16 kb) [file 12916_2019_1340_MOESM11_ESM.docx]

Additional file 11. Summary calibration slope

| **Model** | **Calibration slope** | **95% CI** | **95% PI** |
| --- | --- | --- | --- |
| Wilson men | 1.01 | 0.95-1.07 | 0.95-1.07 |
| Wilson women | 0.97 | 0.71-1.22 | -0.06-2.00 |
| ATP III men | 1.29 | 0.97-1.82 | 0.14-2.45 |
| ATP III women | 0.95 | Not estimable | 0.87-1.03 |
| PCE men | 0.95 | 0.79-1.10 | -0.19-2.07 |
| PCE women | 0.82 | 0.77-0.86 | 0.28-1.35 |

CI: confidence interval, PI: prediction interval

Meta-analysis of stratified OE ratios indicated that miscalibration of the Framingham models was mostly related to heterogeneity in baseline risk, as the summary calibration slope is close to 1. A calibration slope between 0 and 1 indicates predictions are too extreme, e.g. too low for low-risk people and too high for high-risk people. A calibration slope >1 indicates there is not enough variability in predicted risks [1].

**References**

1. Debray TP, Vergouwe Y, Koffijberg H, Nieboer D, Steyerberg EW, Moons KG. A new framework to enhance the interpretation of external validation studies of clinical prediction models. J Clin Epidemiol. 2014. Epub 2014/09/03. doi: 10.1016/j.jclinepi.2014.06.018. PubMed PMID: 25179855.
