# Supplementary material for: Performance of the Framingham risk models and pooled cohort equations for predicting 10-year risk of cardiovascular disease: a systematic review and meta-analysis
Source: BMC Med. 2019 Jun 13;17:109. doi: 10.1186/s12916-019-1340-7 (PMC6563379; doi:10.1186/s12916-019-1340-7)
Supplement: Supplementary file 12 — Sensitivity analyses. Table with results from sensitivity analyses. (DOCX 18 kb) [file 12916_2019_1340_MOESM12_ESM.docx]

Additional file 12. Sensitivity analyses

|  |  | Wilson men |  | Wilson women |  | ATPIII men |  | ATPIII women |  | PCE men |  | PCE women |
| --- | --- | --- | --- | --- | --- | --- | --- | --- | --- | --- | --- | --- |
| **OE ratio** | N | OE (95%CI) | N | OE (95%CI) | N | OE (95%CI) | N | OE (95%CI) | N | OE (95%CI) | N | OE (95%CI) |
| All validations | 16 | 0.580 (0.434-0.726) | 10 | 0.685 (0.442-0.928) | 5 | 0.581 (0.368-0.793) | 4 | 0.785 (0.596-0.974) | 19 | 0.661 (0.591-0.731) | 20 | 0.763 (0.646-0.881) |
| Low risk of bias for all domains* | 1 | - | 1 | - | 4 | - | 1 | - | 2 | - | 3 | - |
| Low risk of bias for participant selection, predictors and outcome | 10 | 0.628 (0.440-0.817) | 6 | 0.720 (0.343-1.097) | 4 | 0.581 (0.335-0.827) | 4 | 0.785 (0.596-0.974) | 16 | 0.683 (0.607-0.760) | 17 | 0.797 (0.676-0.918) |
| Weighted by number of events | 16 | 0.580 (0.434-0.726) | 10 | 0.685 (0.442-0.928) | 5 | 0.557 (0.369-0.744) | 4 | 0.784 (0.595-0.974) | 19 | 0.660 (0.593-0.727) | 20 | 0.781 (0.656-0.905) |
| Bivariate analyses | 18 | 0.547 (0.384-0.384) | 10 | 0.594 (0.387-0.91) | 6 | 0.643 (0.44-0.94) | 5 | 0.723 (0.559-0.936) | 20 | 0.659 (0.596-0.728) | 21 | 0.753 (0.645-0.878) |
| Not extrapolated to 10 year | 16 | 0.575 (0.428-0.721) | 10 | 0.676 (0.429-0.923) | 5 | 0.581 (0.368-0.793) | 4 | 0.785 (0.596-0.974) | 19 | 0.657 (0.587-0.728) | 20 | 0.763 (0.646-0.880) |
|  | | | | | | | | | | | | |
| **C-statistic** | N | C (95%CI) | N | C (95%CI) | N | C (95%CI) | N | C (95%CI) | N | C (95%CI) | N | C (95%CI) |
| All validations | 18 | 0.676 (0.659-0.693) | 10 | 0.706 (0.657-0.756) | 5 | 0.636 (0.594-0.679) | 4 | 0.660 (0.648-0.673) | 20 | 0.701 (0.679-0.723) | 20 | 0.741 (0.719-0.763) |
| Low risk of bias for all domains* | 2 | - | 2 | - | 4 | - | 2 | - | 2 | - | 2 | - |
| Low risk of bias for participant selection, predictors and outcome | 12 | 0.680 (0.659-0.702) | 8 | 0.706 (0.647-0.766) | 4 | 0.642 (0.588-0.696) | 4 | 0.660 (0.648-0.673) | 16 | 0.711 (0.689-0.734) | 16 | 0.751 (0.729-0.774) |
| Weighted by number of events | 18 | 0.675 (0.657-0.694) | 10 | 0.690 (0.643-0.736) | 5 | 0.638 (0.595-0.68) | 4 | 0.658 (0.648-0.669) | 20 | 0.701 (0.679-0.724) | 20 | 0.742 (0.72-0.765) |
| Bivariate analyses | 18 | 0.676 (0.660-0.691) | 10 | 0.707 (0.655-0.754) | 6 | 0.629 (0.588-0.668) | 5 | 0.660 (0.640-0.680) | 20 | 0.701 (0.677-0.724) | 21 | 0.740 (0.718-0.761) |

*No summary statistics are reported because of the low number of validations. OE: observed expected.
