# Supplementary material for: Performance of the Framingham risk models and pooled cohort equations for predicting 10-year risk of cardiovascular disease: a systematic review and meta-analysis
Source: BMC Med. 2019 Jun 13;17:109. doi: 10.1186/s12916-019-1340-7 (PMC6563379; doi:10.1186/s12916-019-1340-7)
Supplement: Supplementary file 13 — Meta-regression analyses. Figures with results from meta-regression analyses. (DOCX 141 kb) [file 12916_2019_1340_MOESM13_ESM.docx]

Additional file 13. Metaregression analyses

OE ratio

A B

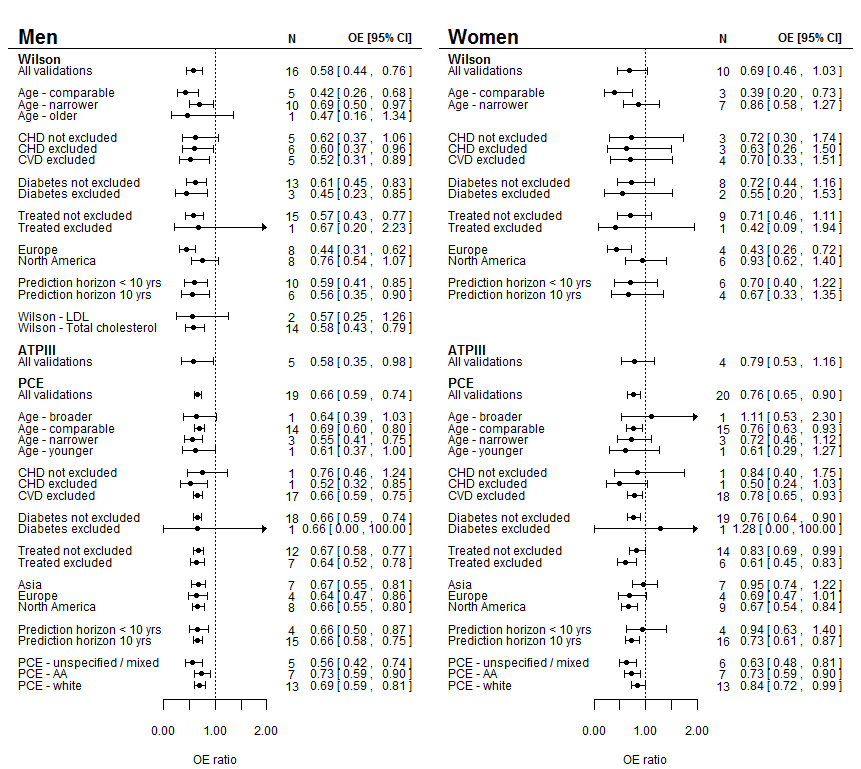


C
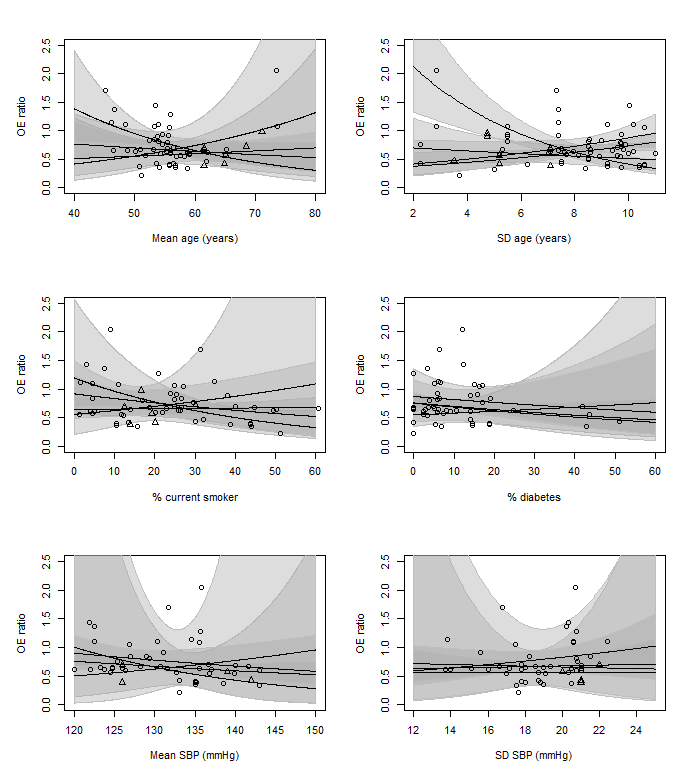

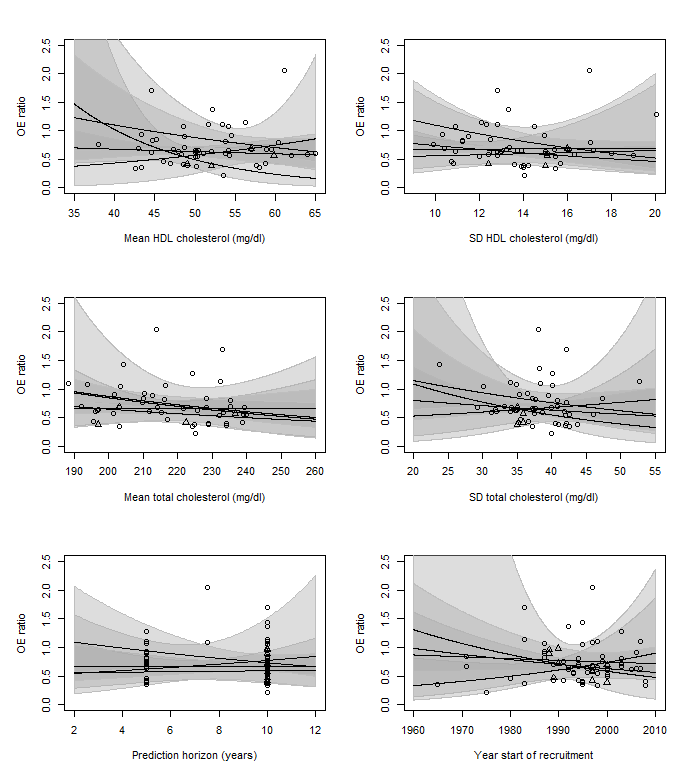
Figure: Results of meta-regression OE ratio for categorical variables (A and B) and continuous variables (C). For C, Every line represents one model: Wilson men, Wilson women, PCE men or PCE women. ATP III is not plotted because of the low number of external validations, but the triangles represent the individual validations for the ATP III models. The grey areas represent the confidence intervals around the lines, and the circles represent the individual external validations. CHD: coronary heart disease, CVD: cardiovascular disease, AA: African American, SD: standard deviation, SBP: systolic blood pressure, HDL: high-density lipoprotein.

C-statistic

A B


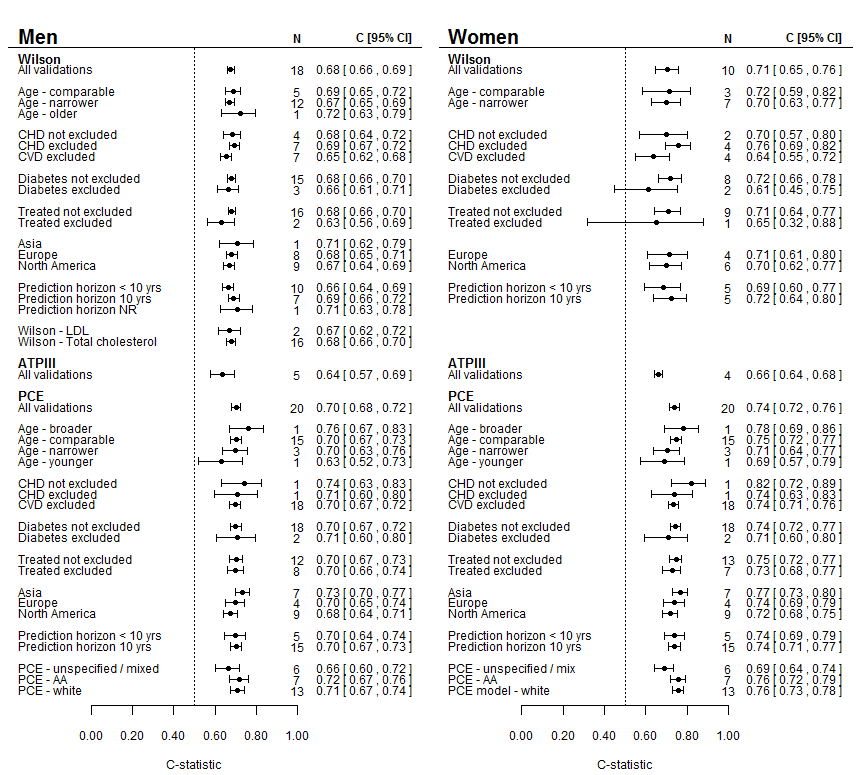


C
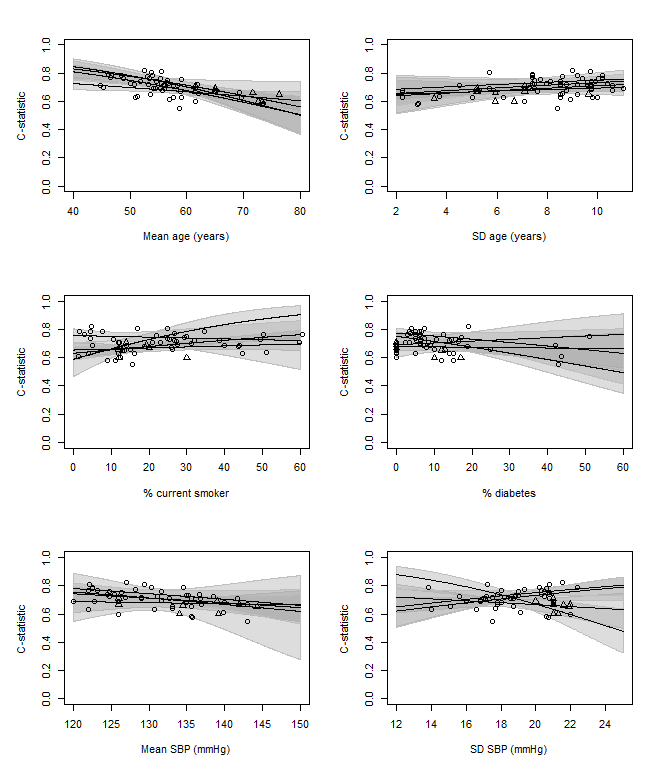

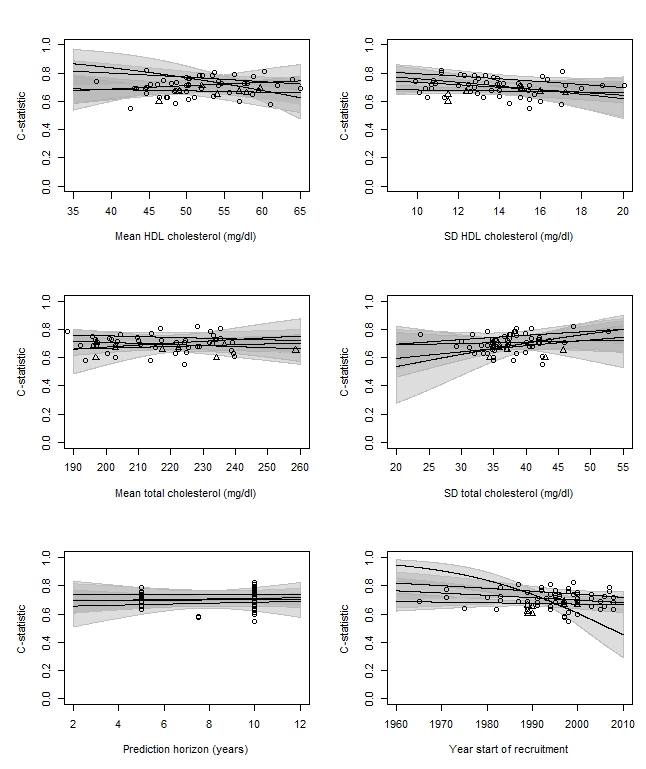
Figure: Results of meta-regression c-statistic for categorical variables (A and B) and continuous variables (C). For C, Every line represents one model: Wilson men, Wilson women, PCE men or PCE women. ATP III is not plotted because of the low number of external validations, but the triangles represent the individual validations for the ATP III models. The grey areas represent the confidence intervals around the lines, and the circles represent the individual external validations. CHD: coronary heart disease, CVD: cardiovascular disease, AA: African American, SD: standard deviation, SBP: systolic blood pressure, HDL: high-density lipoprotein.
